# Evolution of Gross Forelimb and Fine Digit Kinematics during Skilled Reaching Acquisition in Rats

**DOI:** 10.1523/ENEURO.0153-21.2021

**Published:** 2021-10-26

**Authors:** Alexandra Bova, Kenneth Ferris, Daniel K. Leventhal

**Affiliations:** 1 Neuroscience Graduate Program, University of Michigan, Ann Arbor, MI 48109; 2 Department of Neurology, University of Michigan, Ann Arbor, MI 48109; 3 Department of Biomedical Engineering, University of Michigan, Ann Arbor, MI 48109; 4 Parkinson Disease Foundation Research Center of Excellence, University of Michigan, Ann Arbor, MI 48109; 5 Department of Neurology, VA Ann Arbor Health System, Ann Arbor, MI 48109

**Keywords:** kinematics, motion tracking, motor learning, rat, skilled reaching

## Abstract

The ability to learn dexterous motor skills is a fundamental aspect of human behavior. However, the underlying neural circuit mechanisms for dexterous skill learning are unclear. Advancing our understanding of motor skill learning requires the integration of modern neuroscientific techniques with a rigorously characterized dexterous task. The development of automated rodent skilled reaching with paw tracking allows detailed analysis of how reach-to-grasp kinematics evolve during learning. We assessed how both “gross” forelimb and “fine” digit kinematics changed as rats learned skilled reaching. Rats whose success rates increased (learners) consistently reduced the variability in their reach trajectories. Refinement of fine digit control generally continued after consistency in gross hand transport to the pellet plateaued. Interestingly, most rats whose success rates did not increase (non-learners) also converged on consistent reach kinematics. Some non-learners, however, maintained substantial variability in hand and digit trajectories throughout training. These results suggest that gross and fine motor components of dexterous skill are, on average, learned over different timescales. Nonetheless, there is significant intersubject variability in learning rates as assessed by both reaching success and consistency of reach kinematics.

## Significance Statement

Humans depend heavily on the ability to learn novel, dexterous motor skills. The rodent skilled reaching task is commonly used to study dexterous skill learning. However, how reach-to-grasp kinematics evolve as rats learn skilled reaching has not been carefully characterized. By combining an automated rodent skilled reaching task with hand and digit tracking, we found significant variability in both success rates and the evolution of hand/digit kinematics. This interindividual variability is important for interpreting skilled reaching studies. Furthermore, understanding the neurobiologic basis of this variability may yield insights into improving human motor learning and rehabilitation.

## Introduction

Dexterous motor skills, or coordinated multijoint and digit movements, are acquired through practice and essential to normal daily function. However, the neural mechanisms of motor skill learning are poorly understood. Such knowledge is important for improving *de novo* skill acquisition, understanding disorders of motor learning (e.g., dystonia), and optimizing rehabilitation after neurologic injury (e.g., poststroke). To determine mechanisms of motor learning at the neuronal/circuit level, animal models of skill acquisition are needed. Such models allow the application of modern circuit dissection techniques, which are especially well developed in rodents.

Rodent skilled reaching is a common paradigm for studying dexterous skill learning. Rodent reach-to-grasp movements are strikingly similar to those of humans (Klein et al., 2012). This is true when comparing well-trained rats to healthy human subjects or rodent disease models (e.g., dopamine-depleted or cortically lesioned rats) to humans with motor system dysfunction (e.g., Parkinson’s disease or cortical strokes; Miklyaeva et al., 1994; Whishaw et al., 1994; Klein et al., 2011, 2012). Therefore, this task is well suited for studying the neural circuit mechanisms that regulate dexterous motor control under “normal” conditions and in disease states. Furthermore, rodents are not naturally adept at this task, but gradually improve over several training sessions (Whishaw et al., 2008). The rodent skilled reaching task is therefore useful for studying motor skill learning as well as performance. However, how reach-to-grasp movements evolve as rats learn this task has not been carefully characterized.

Learning in the skilled reaching task has typically been assessed by success rate (i.e., whether the pellet was grasped or not). Therefore, studies could describe if, but not how, reach-to-grasp movements changed. To address this, a semi-quantitative movement element rating system was developed, wherein each submovement of the reach is evaluated as “normal,” “abnormal,” or “absent” (Whishaw et al., 2008; Alaverdashvili and Whishaw, 2010). This system is useful for assessing how experimental manipulations (e.g., targeted lesions) affect reaching movements in well-trained animals, but is less informative for learning. Additionally, it still only provides information on if an individual submovement is abnormal or missing, not how the submovement changed. Finally, this scoring system is time-intensive and subjective, as it requires experimenters to evaluate twelve movement elements individually for each video.

Recent studies have leveraged automated skilled reaching systems and position tracking software to assess changes in reach-to-grasp kinematics over learning with greater detail. Rats’ forelimb trajectories became less variable and the duration of reaching movements decreased as success rate increased. However, success rate continued to improve even once these measures of “gross” forelimb kinematics had plateaued (Li et al., 2017; Lemke et al., 2019). Therefore, it appears that “fine” motor components (i.e., individual digit control) evolve on a different timescale than the “gross” motor components of reach-to-grasp movements. However, neither of these studies directly measured changes in fine digit control. It is, therefore, unknown exactly how individual digit control changes as rats learn to perform skilled reaching.

In this study, we assessed how “gross” and “fine” motor components of reach-to-grasp movements evolve as rats learn skilled reaching. By using DeepLabCut (Mathis et al., 2018; Nath et al., 2019) to track individual digit joints and hands, we measured changes in both forelimb and digit kinematics. Consistent with the above findings, we show that for rats whose success rates improve with training, the control of fine digit movements evolves on a longer timescale than that of gross forelimb movements. However, there was significant variability among rats in the timing and magnitude of changes in both gross hand movements and fine digit movements. These results suggest that gross and fine movements are refined over different timescales to improve performance.

## Materials and Methods

### Animals

All animal procedures were performed in accordance with the University of Michigan Institutional Animal Care and Use Committee regulations. Male (*n* = 9) and female (*n* = 5) tyrosine hydroxylase-Cre^+^ (*n* = 9) and wild-type (*n* = 5) rats were housed in groups of two to three on a reverse light/dark cycle. All behavioral training was conducted during the dark phase. Food restriction was imposed on all animals during the training period for no more than 6 d in a row such that rats’ weights were kept ∼85–90% of their free-feeding weight. Rats were only tested on food restricted days. Water was available *ad libitum* in their home cages. Data collected after seven of these rats were well trained have been reported previously. However, none of the “early learning” kinematics described here have been reported previously.

### Skilled reaching

#### Automated reaching system

**Figure 1.**
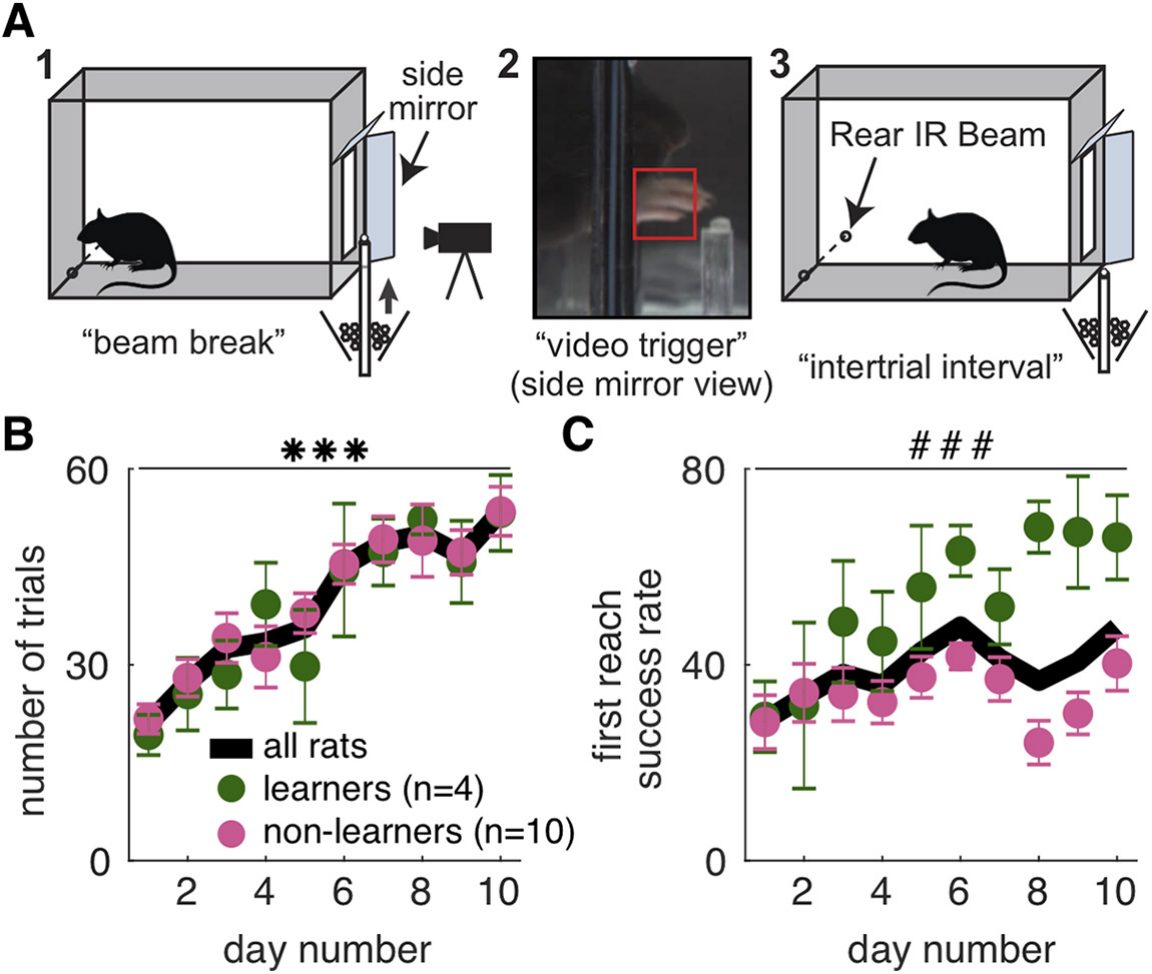
Skilled reaching performance improves with training. ***A***, A single skilled reaching trial. (1) Rat breaks IR beam at the back of the chamber to request a sugar pellet (“beam break”). (2) Real-time analysis detects the hand breaching the reaching slot to trigger 300-fps video from 1 s before to 3.33 s after the trigger event (“video trigger”); (3) 2 s after the trigger event, the pellet delivery rod resets and the rat can initiate a new trial (“intertrial interval”). ***B***, Average number of trials per day. Learners (green) and non-learners (pink) did not differ in the number of trials performed per day (linear mixed model: effect of group: *t*_(28)_ = 0.49, *p* = 0.63). However, both groups increased the number of trials performed over days (linear mixed model: effect of day: *t*_(124)_ = 3.58, *p* = 4.88 × 10^−4^; group × day interaction: *t*_(124)_ = −0.47, *p* = 0.64). Black line represents the average number of trials per day for both groups combined. ***C***, Average first attempt success rate per day. Although both groups had similar success rates on day 1, only learners increased their success rates over days (linear mixed model: effect of group: *t*_(25)_ = 0.44, *p* = 0.66; effect of day: *t*_(123)_ = 5.55, *p* = 1.70 × 10^−7^; group × day interaction: *t*_(123)_ = −4.78, *p* = 4.98 × 10^−6^. Error bars in ***B***, ***C*** represent SEM. Extended Data Figure 1-1 shows individual rat data for each group and additional performance measures (number of attempts per trial and breakdown of reach outcomes across days); ***p < 0.001 for the day term in the linear mixed model in ***B*** and ###*p* < 0.001 for the group × day interaction in the linear mixed model in ***C***. Data and code to generate this figure are contained in Extended Data 1, 2.

Training was conducted in custom-built skilled reaching chambers housed within soundproof, ventilated cabinets. Custom LabVIEW software controls the experiment. The chambers were rectangular prisms with a reaching slot (1.1 × 7 cm) cut in the front panel 3.5 cm from the floor and a photobeam (HoneyWell) at the back of the chamber (Fig. 1*A*). Before each session, a linear actuator (Creative Werks Inc) was positioned so that the pellet delivery rod was aligned with the right or left edge of the slot according to each rat’s hand preference 15 mm from the front of the reaching slot. Each training session began with the pellet delivery rod in the “ready” position, part of the way between the bottom of the reaching chamber and the reaching slot. Rats “requested” a pellet by breaking the photobeam, which caused the pellet delivery rod to rise to the bottom of the reaching slot. Mirrors angled at either side of the front of the reaching chamber allowed side views of the hand during reaches. When the reaching hand passed the front plane of the chamber into the “region of interest” in the side mirror view (Fig. 1*A*, red box) and surpassed the minimum threshold of pixel intensity, video acquisition was triggered, time-stamped, and labeled with the trial number. Two seconds after the video was triggered, the pellet delivery arm lowered into the pellet funnel to pick up a new pellet and then reset to the “ready” position, allowing the rat to initiate a new trial.

10.1523/ENEURO.0153-21.2021.f1-1 Extended Data Figure 1-1 Additional task performance measures. ***A***, Individual rat data for average number of trials performed per day for learners (left) and non-learners (right). Each colored line represents an individual rat. Colors represent the same rat across all individual rat data figures within groups. ***B***, Individual rat data for first reach success rate for learners (left) and non-learners (right). ***C***, Number of reach attempts per trial. Both learners (left) and non-learners (right) decreased the number of reach attempts performed per trial over days (linear mixed model: effect of group: *t*_(19)_ = 0.81, *p* = 0.43; effect of day: *t*_(124)_ = –4.12, *p* = 6.88 × 10^−5^; group × day interaction: *t*_(124)_ = 1.37, *p* = 0.17. Black line represents averaged data. Colored lines represent individual rats. ***D***, Breakdown of trial outcomes by day for learners (green) and non-learners (for definitions of outcomes, see Materials and Methods, Number of trials and success rate). The percentage of “first success” increased over days only for learners (linear mixed model: effect of day: learners: *t*_(35)_ = 4.96, *p* = 1.83 × 10^−5^; non-learners: *t*_(88)_ = 0.51, *p* = 0.61). “Multiple success” outcomes decreased over days for learners but not non-learners (linear mixed model: effect of day: learners: *t*_(35)_ = –2.53, *p* = 0.02; non-learners: *t*_(89)_ = –0.79, *p* = 0.43). Similarly, “pellet knocked off” outcomes decreased for learners but not non-learners over days (linear mixed model: effect of day: learners: *t*_(35)_ = –4.59, *p* = 5.46 × 10^−5^; non-learners: *t*_(89)_ = 0.49, *p* = 0.62). All other outcomes were consistent across days for both groups. Linear mixed model: effect of day (learners): no pellet: *t*_(35)_ = –0.11, *p* = 0.91; drop in box; *t*_(35)_ = 0.42, *p* = 0.68; tongue: *t*_(35)_ = 0; *p* = 1; trigger error: *t*_(38)_ = –0.52, *p* = 0.61; pellet remained: *t*_(38)_ = –0.78, *p* = 0.44; non-preferred hand: *t*_(38)_ = –0.87, *p* = 0.39; tongue and hand: *t*_(38)_ = 0, *p* = 1; hand through slot: *t*_(38)_ = –0.78, *p* = 0.44. Linear mixed model: effect of day (non-learners): no pellet: *t*_(98)_ = –0.62, *p* = 0.54; drop in box; *t*_(89)_ = 0.36, *p* = 0.72; tongue: *t*_(98)_ = 0; *p* = 1; trigger error: *t*_(98)_ = –1.37, *p* = 0.18; pellet remained: *t*_(98)_ = –0.95, *p* = 0.35; non-preferred hand: *t*_(98)_ = –0.87, *p* = 0.39; tongue and hand: *t*_(98)_ = 0, *p* = 1; hand through slot: *t*_(98)_ = 0.01, *p* = 0.99; ***p < 0.001 for the day term in the linear mixed model in ***C***, ***D***; *p < 0.05 for the day term in the linear mixed model in D. Data and code to generate this figure are contained in Extended Data 1, 2. Download Figure 1-1, TIF file.

10.1523/ENEURO.0153-21.2021.ed1 Extended Data 1 MATLAB and Python code for kinematic analyses and figure plotting. *Bova_Leventhal_code.zip* contains custom MATLAB software for reconstruction of 3D reach trajectories, processing reach-to-grasp kinematics, and creating the figures in this manuscript using data in the other Extended Data files. Download Extended Data 1, ZIP file.

10.1523/ENEURO.0153-21.2021.ed2 Extended Data 2 *eNeuro_summary_data.zip* contains .mat files used by the code in Bova_Leventhal_code.zip to create all main and Extended Data figures, except for Extended Data Figures 6-3–6-16*B–D*. Download Extended Data 2, ZIP file.

Videos were recorded at 300 frames/s and 2400 × 1024 pixels by a high-definition color camera (acA2000-340kc, Basler) mounted in front of the reaching slot. A camera-link field-programmable gate array (FPGA) frame-grabber card (PCIe 1473R, National Instruments) acquired the images, and an FPGA data acquisition (DAQ) task control card (NI PCIe 7841R) provided an interface with the behavior chamber. The real-time FPGA card detected pixel intensity changes within a “region of interest” in front of the reaching slot in the side mirror views (Fig. 1*A*), allowing videos of the reaching event (“video trigger”) to be captured. Video frames were stored in RAM in a rolling buffer. When the trigger event occurred, 300 frames before the trigger event and 1000 frames posttrigger were saved.

#### Pretraining

“Pretraining” consists of familiarizing the rats with the reaching chamber, evaluating them for hand preference, training them to reach for the linear actuator, and training them to request a pellet by moving to the back of the chamber. A week before pretraining, rats were placed on food restriction and introduced to the sucrose reward pellets in their home cages. On day 1 of pretraining, piles of five pellets each were placed in the front and rear of the skilled reaching chamber to encourage exploration of the entire chamber. Once rats ate these pellets, they were evaluated for hand preference.

Rats were allowed to eat three pellets (held in forceps through the reaching slot) with their tongues. The experimenter then began to pull the pellet away from the rat so that it could not be obtained by licking. Therefore, the rat was forced to reach with its hand to retrieve the pellet. Hand preference was assigned to the hand used for the majority of the first eleven reaches. Once hand preference was determined, animals were trained to reach for the pellet delivery rod. As the rat reached, the experimenter pulled the forceps back so that the rat’s hand would extend to a pellet on the delivery rod. Once rats reached for the delivery rod 10 times without being baited by the experimenter, they began training to request pellets.

Rats began training in the center of the chamber with the pellet delivery rod set to the “ready” position. The experimenter placed a pellet in the rear of the chamber to bait the rat to break the rear IR beam, causing the delivery rod to rise so that the rat could move to the front and reach for the pellet. This was repeated until the rat began to quickly move to the front of the chamber to reach for the pellet after breaking the IR beam. At this point, the experimenter would stop baiting the rat to the rear of the chamber. Pretraining was complete once the rat requested a pellet and then immediately moved to the front to reach for the pellet 10 times.

#### Training

Once pretrained, rats began 30-min training sessions with the automated system. Rats were trained for one session per day for 10 d, with 1 d off when rats were taken off food restriction. The day number during training when the break occurred was varied (e.g., not all rats had a break between days 5 and 6) to minimize effects of a day off on skilled reaching performance.

### Analysis of skilled reaching data

Analyses were performed using custom-written scripts and functions in MATLAB 2019a (MathWorks) on a MacBook Pro (2018) with macOS Mojave (version 10.14.6; Apple).

#### Performance outcomes

Reach outcome was scored by visual inspection as follows: 0, no pellet presented or other mechanical failure (“no pellet”); 1, obtained pellet on initial limb advance (“first success”); 2, obtained pellet, but not on first attempt (“multiple success”); 3, forelimb advanced, pellet was grasped then dropped in the box (“drop in box”); 4, forelimb advance, but the pellet was knocked off the shelf (“pellet knocked off”); 5, pellet was obtained using its tongue (“tongue”); 6, the rat approached the slot but retreated without advancing its forelimb or the video triggered without a reach (“trigger error”); 7, the rat reached, but the pellet remained on the shelf (“pellet remained”); 8, the rat used its non-preferred hand to reach (“non-preferred hand”); or 9, used preferred hand after obtaining or moving pellet with tongue (“tongue and hand”). An additional outcome evaluated by kinematic analysis was defined as videos which began with the rat’s hand through the slot (i.e., the video triggered late; “hand through slot”). Outcome percent was calculated by dividing the number of trials of each outcome by the total number of trials per training day (Extended Data Fig. 1-1*D*). For comparisons of kinematics between successful and failed reaches, successes were defined as “first success” (1) and failed reaches were defined as “pellet knocked off” (4) or “pellet remained” (7).

First reach success was calculated for each training day by dividing the total number of scores of 1 by the total number of trials (sum of scores of 1, 2, 3, 4, and 7; Fig. 1*C*; Extended Data Fig. 1-1*B*). Success rate was not calculated for individual days if <10 trials were performed (only one such instance occurred).

Rats were divided into learners and non-learners based on the change in their performance between the first two training days and last two training days. The proportion of first success reaches from days 1 and 2 combined were compared with the proportion of first success reaches from days 9 and 10 combined using a χ^2^ test. Rats with *p* < 0.05 whose success rate increased between days 1–2 and 9–10 were considered learners (*n* = 4); all others were considered non-learners (*n* = 10). All subsequent analyses of reach-to-grasp kinematics were performed with rats divided into learners and non-learners, with averages across all rats also shown.

**Figure 2.**
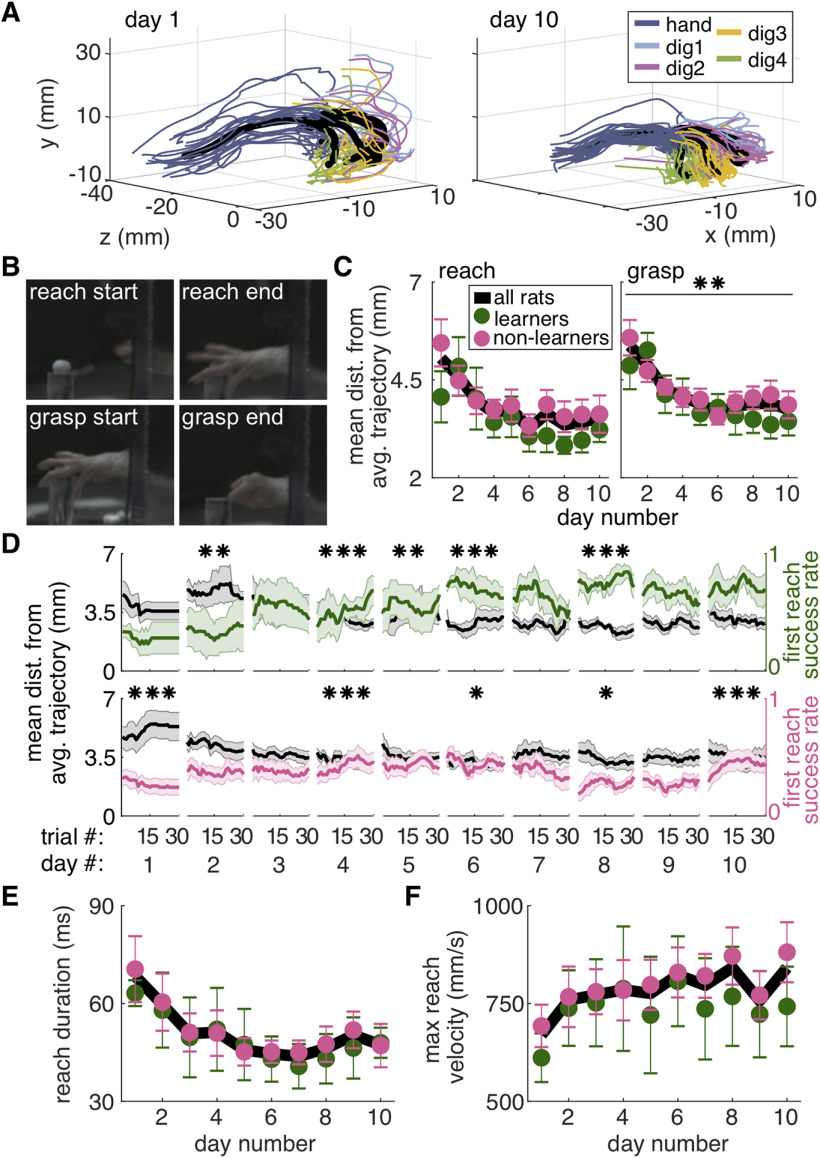
Refinement of “gross” forelimb movements. ***A***, All 3D reaching trajectories on days 1 and 10 from an exemplar rat. Colored lines represent individual trials and black lines represent average trajectories of the hand and digit tips. Sugar pellet (black dot) is at (0,0,0). ***B***, Hand trajectories are separated into “reach” and “grasp” components. The reach begins when the digits are first visible outside the reaching box and ends when the second digit begins to retract. The grasp begins when the second digit begins to flex and ends when the second digit is maximally flexed after grasp start. ***C***, Average hand trajectory variability for the reach and grasp components represented as the mean distance from the average trajectory (mm). Trajectory variability did not differ significantly between learners and non-learners for either component (linear mixed model: effect of group: reach: *t*_(23)_ = 0.53, *p* = 0.60; grasp: *t*_(26)_ = −0.08, *p* = 0.94; group × day interaction: reach: *t*_(124)_ = 0.37, *p* = 0.71; grasp: *t*_(124)_ = 1.08, *p* = 0.28). Trajectory variability of the grasp, but not reach, decreased significantly over days for both groups (linear mixed model: effect of day: reach: *t*_(124)_ = −1.85, *p* = 0.07; grasp: *t*_(124)_ = −2.75, *p* = 6.91 × 10^−3^). Individual rat data are shown in Extended Data Figure 2-1. ***D***, Moving average of hand trajectory variability for the reach component (black) and first attempt success rate (green learners and pink non-learners) within individual days. Days 2, 4, 5, 6, and 8 had significant negative correlations between trajectory variability and success rate for the learners group (day 2: *r* = −0.55, *p* = 1.70 × 10^−3^; day 4: *r* = −0.68, *p* = 4.25 × 10^−5^; day 5: *r* = −0.53, *p* = 2.50 × 10^−3^; day 6: *r* = −0.82, *p* = 2.16 × 10^−8^; day 8: *r* = −0.70, *p* = 1.51 × 10^−5^). Days 1, 4, 6, 8, and 10 had significant negative correlations between trajectory variability and success rate for the non-learners group (day 1: *r* = −0.88, *p* = 1.78 × 10^−10^; day 4: *r* = −0.67, *p* = 4.33 × 10^−5^; day 6: *r* = −0.38, *p* = 0.04; day 8: *r* = −0.42, *p* = 0.02; day 10: *r* = −0.83, *p* = 1.53 × 10^−8^). ***E***, Average reach duration for learners (green) and non-learners (pink). Linear mixed model: effect of group: *t*_(21)_ = 0.29, *p* = 0.78; effect of day: *t*_(124)_ = −1.03, *p* = 0.31; group × day interaction: *t*_(124)_ = −0.11, *p* = 0.91. Individual rat data are shown in Extended Data Figure 2-1. ***F***, Average maximum reach velocity (mm/s) for learners (green) and non-learners (pink). Linear mixed model: effect of group: *t*_(15)_ = 0.16, *p* = 0.87; effect of day: *t*_(124)_ = −0.07, *p* = 0.94; group × day interaction: *t*_(124)_ = 1.02, *p* = 0.31. Individual rat data are shown in Extended Data Figure 2-1. Error bars in ***C***, ***E***, ***F***, and shaded colored areas in ***D*** represent SEM; *p < 0.05, **p < 0.01, ***p < 0.001 for a negative correlation between success rate and trajectory variability in ***D***; **p < 0.05 for the day term in the linear mixed model in ***C***. Data and code to generate this figure are contained in Extended Data 1, 2.

To assess how success rate changed within individual sessions, a moving average was calculated as the fraction of “1” (first success) scores in a moving block of 10 reaches. For averages within a group, the last data point for each individual was carried forward to the maximum number of reaches for any rat on that training day. This avoided sudden changes in the average caused by dropout (Fig. 2*D*).

10.1523/ENEURO.0153-21.2021.f2-1 Extended Data Figure 2-1 Individual rat data for hand trajectory variability, reach duration, and reach velocity. ***A***, Average hand trajectory variability of the reach component for learners (left) and non-learners (right) represented as the mean distance from the average trajectory (mm). ***B***, Average hand trajectory variability of the grasp component for learners (left) and non-learners (right) represented as the mean distance from the average trajectory (mm). ***C***, Average reach duration (ms) for learners (left) and non-learners (right). ***D***, Maximum reach velocity (mm/s) for learners (left) and non-learners (right). Data and code to generate this figure are contained in Extended Data 1, 2. Download Figure 2-1, TIF file.

#### 3D reconstruction of reach trajectories

**Figure 3.**
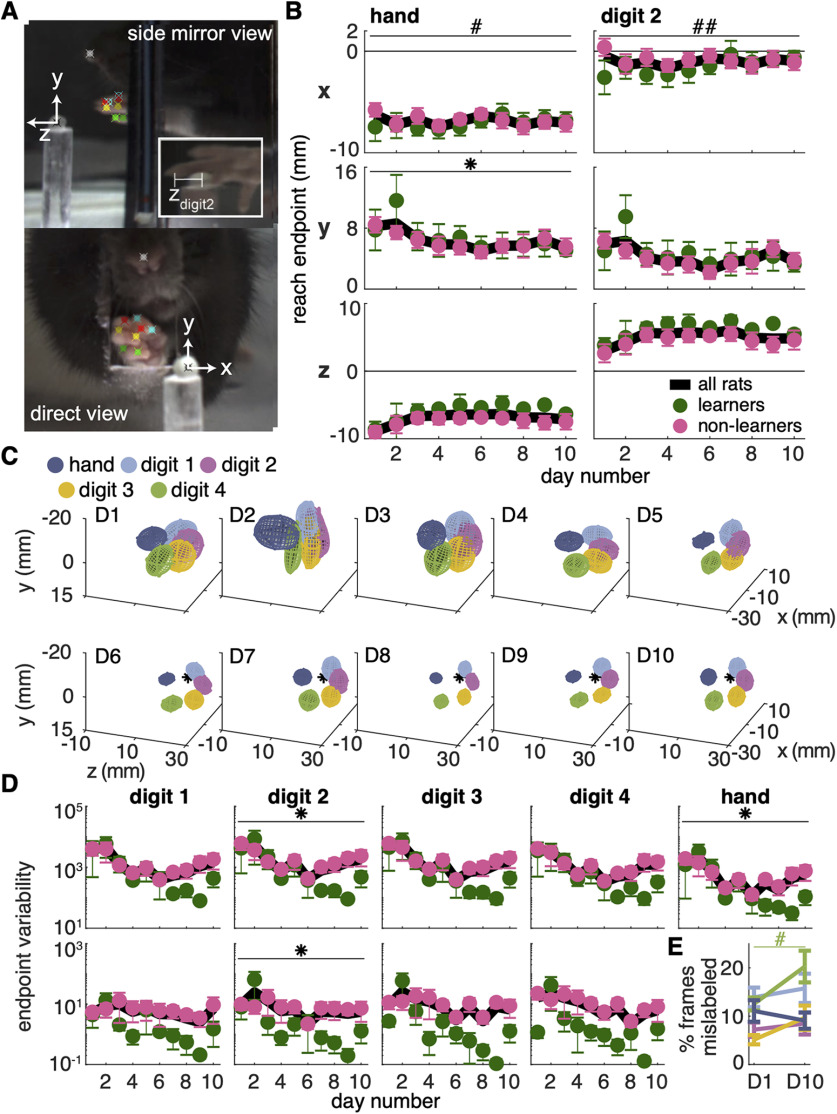
Reach endpoint accuracy improves with training. ***A***, *X*, *Y*, and *Z* reach endpoints are measured in reference to the pellet position. Top, Mirror view of the dorsal surface of the hand. Bottom, Direct view from the camera. The inset shows the mirror view of the palmar surface of the hand to demonstrate how reach endpoint of the second digit tip in the *Z* direction (z_digit2_) is measured. The end of the reach is defined as the moment z_digit2_ begins to decrease (the digit tip moves back toward the box). ***B***, Average reach endpoints of the hand (left) and digit 2 (right) in the *X*, *Y*, and *Z* directions. Pellet is at (0,0,0). Reach endpoint in the *X* direction increased over days for learners but not non-learners. Linear mixed model (hand): effect of group: *t*_(18)_ = 1.30, *p* = 0.21; effect of day: *t*_(124)_ = 2.13, *p* = 0.04; group × day interaction: *t*_(124)_ = −2.40, *p* = 0.02; digit 2: effect of group: *t*_(17)_ = 1.80, *p* = 0.09; effect of day: *t*_(124)_ = 3.12, *p*= 2.25 × 10^−3^; group × day interaction: *t*_(124)_ = −3.16, *p* = 1.97 × 10^−3^. Reach endpoint of the hand in the *Y* plane decreased with training for both groups. Linear mixed model (hand): effect of group: *t*_(18)_ = −0.94, *p* = 0.36; effect of day: *t*_(124)_ = −2.45, *p* = 0.02; group × day interaction: *t*_(124)_ = 1.45, *p* = 0.15; digit 2: effect of group: *t*_(19)_ = −0.90, *p* = 0.38; effect of day: *t*_(124)_ = −1.80, *p* = 0.07; group × day interaction: *t*_(124)_ = 1.16, *p* = 0.25. Reach endpoint in the *Z* plane did not change significantly with training for either group. Linear mixed model (hand): effect of group: *t*_(23)_ = −0.38, *p* = 0.71; effect of day: *t*_(124)_ = 1.73, *p* = 0.09; group × day interaction: *t*_(124)_ = −1.23, *p* = 0.22; digit 2: effect of group: *t*_(19)_ = −0.63, *p* = 0.53; effect of day: *t*_(124)_ = 0.99, *p* = 0.33; group × day interaction: *t*_(124)_ = −0.58; *p* = 0.57. Extended Data Figure 3-1 shows individual rat data. ***C***, 3D covariance of hand and digit reach endpoints across all days from an exemplar rat. Sugar pellet (*) is at (0,0,0). ***D***, Average determinant of the covariance matrix (generalized variance) of reach endpoints for the hand and digits. Top row shows endpoint variability of “raw” digit and hand position data (linear mixed model: effect of day: digit 1: *t*_(124)_ = −1.97, *p* = 0.05; digit 2: *t*_(124)_ = −2.25, *p* = 0.03; digit 3: *t*_(124)_ = −1.85, *p* = 0.07; digit 4: *t*_(124)_ = −1.78, *p* = 0.08; hand: *t*_(124)_ = −2.27, *p* = 0.02). Bottom row shows endpoint variability of digit positions subtracted from hand position (linear mixed model: effect of day: digit 1: *t*_(124)_ = −0.87, *p* = 0.39; digit 2: *t*_(124)_ = −2.23, *p* = 0.03; digit 3: *t*_(136)_ = −1.64, *p* = 0.10; digit 4: *t*_(124)_ = −0.67, *p* = 0.51). Extended Data Figure 3-2 shows individual rat data. ***E***, Average percentage of frames from reach start to grasp end that were mislabeled by DeepLabCut for the hand (dark blue) and digits 1–4 (light blue, pink, yellow, and green, respectively) on days 1 and 10. The percentage of mislabeled frames did not differ between parts (linear mixed model: effect of part: *t*_(123)_ = −0.05, *p* = 0.96) or days overall (linear mixed model: effect of day: *t*_(123)_ = 0.98, *p* = 0.33). The percentage of mislabeled frames increased significantly between day 1 and day 10 for digit 4 only (linear mixed model: part × day interaction: hand: *t*_(117)_ = −0.79, *p* = 0.43; digit 1: *t*_(117)_ = 1.07, *p* = 0.29; digit 2: *t*_(117)_ = 0.91, *p* = 0.37; digit 3: *t*_(117)_ = 1.82, *p* = 0.07; digit 4: *t*_(117)_ = 2.71, *p* = 7.74 × 10^−3^). Error bars in ***B***, ***D***, ***E***, represent SEM; *p < 0.05 for the day term, #*p* < 0.05, ##*p* < 0.01 for the group × day interaction in the linear mixed model in ***B***, ***D***; #*p* < 0.05 for the part × day interaction in ***E***. Data and code to generate this figure are contained in Extended Data 1, 2.

Bodyparts/objects identified in the direct and mirror views were triangulated to 3D points using custom MATLAB software. Before each training session, several images of a cube with checkerboards (4 × 4 mm squares) on its sides were taken so that the checkerboards were visible in the direct and mirror views. These images were used to determine the essential matrix relating the direct and mirror views, which was used to determine how the real camera and “virtual” camera behind the mirror were translated and rotated with respect to each other (Hartley and Zisserman, 2003). By assuming a 3D coordinate system centered at the camera lens with the *z*-axis perpendicular to the lens surface, camera matrices were derived for the real and virtual cameras. These matrices were used to triangulate matching points in the camera and mirror views using the MATLAB *triangulate* function in the Computer Vision toolbox. 3D points with large reprojection errors were excluded from the analysis, which could happen if an object was identified accurately in one view but misidentified in the other. The coordinate system was set with the pellet at the origin, positive x to the right of the pellet, positive y above the pellet, and positive z on the other side of the pellet from the reaching chamber (Fig. 3*A*).

10.1523/ENEURO.0153-21.2021.f3-1 Extended Data Figure 3-1 Individual rat data for reach endpoints. Average reach endpoints of the hand (left) and digit 2 (right) for learners (left columns) and non-learners (right columns) in the *X*, *Y*, and *Z* directions. Pellet is at (0,0,0). Data and code to generate this figure are contained in Extended Data 1, 2. Download Figure 3-1, TIF file.

10.1523/ENEURO.0153-21.2021.f3-2 Extended Data Figure 3-2 Individual rat data for endpoint variability. Average determinant of the covariance matrix (generalized variance) of reach endpoints for the hand and digits. For digits, left two columns show endpoint variability of “raw” digit positions and right two columns show endpoint variability of digit positions subtracted from the hand position. Data and code to generate this figure are contained in Extended Data 1, 2. Download Figure 3-2, TIF file.

#### Processing reach kinematics

To place reach kinematics in a common reference frame, the pellet location before reaching was identified and set as the origin. For left-handed reaches, x-coordinates were negated to allow direct comparison with right-handed reaches. The initial reach on each trial was identified by finding the first frame in which digits were visible outside the box (Fig. 2*B*), and then looking backwards in time until the hand started moving forward. The end of a reach was defined as the frame at which the tip of the second digit began to retract; multiple reaches could be counted in a single trial (though only first reaches are analyzed here). The start of the grasp was defined as the frame at which flexion of the second digit started to increase after reaching minimum flexion (i.e., maximum extension). Grasp end was defined as the first frame with maximum digit flexion after grasp start. The end of a reach and beginning of a grasp could overlap if rats started to flex their digits as the hand was still advancing. However, this was rare and the number of overlapping frames was small.

**Figure 4.**
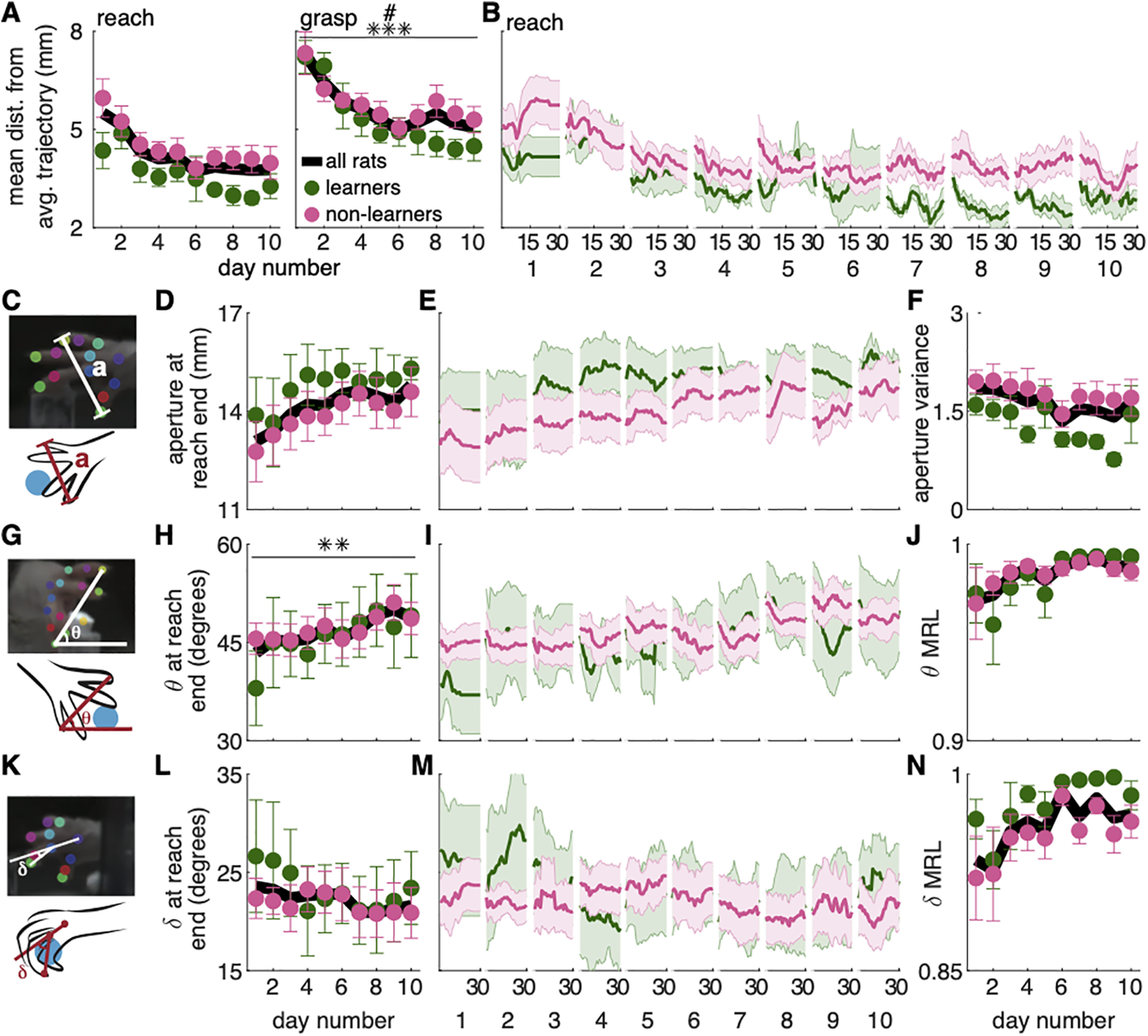
Refinement of “fine” digit movements. ***A***, Average digit 2 trajectory variability for the reach and grasp components represented as the mean distance from the average trajectory (mm). Trajectory variability significantly decreased over days during the grasp, but not reach, component (linear mixed model: effect of day: reach: *t*_(124)_ = −1.70, *p* = 0.09; grasp: *t*_(124)_ = −3.92, *p* = 1.46 × 10^−4^). Trajectory variability of the grasp was significantly lower for learners than non-learners in the later training days (group × day interaction: reach: *t*_(124)_ = −0.01, *p* = 0.99; grasp: *t*_(124)_ = 2.29, *p* = 0.02). Individual rat data are shown in Extended Data Figure 4-1. ***B***, Moving average of digit 2 trajectory variability for the reach component for learners (green) and non-learners (pink) within individual days. ***C***, Grasp aperture (*a*) is the Euclidian distance between the first and fourth digit tips. ***D***, Average grasp aperture at reach end for learners (green) and non-learners (pink). Linear mixed model: effect of group: *t*_(15)_ = −0.81, *p* = 0.43; effect of day: *t*_(124)_ = 0.97, *p* = 0.34; group × day interaction: *t*_(124)_ = 0.36, *p* = 0.72. Individual rat data are shown in Extended Data Figure 4-1. ***E***, Moving average of grasp aperture at reach end within days for learners (green) and non-learners (pink). ***F***, Average aperture variance at reach end. Linear mixed model: effect of group: *t*_(22)_ = 0.96, *p* = 0.35; effect of day: *t*_(124)_ = −1.53, *p* = 0.13; group × day interaction: *t*_(124)_ = 0.90, *p* = 0.37. Individual rat data are shown in Extended Data Figure 4-1. ***G***, Hand orientation is the angle (θ) between a line connecting the first and fourth digit tips and the floor. ***H***, Average hand orientation at reach end increased over days but did not differ between groups. Linear mixed model: effect of group: *t*_(15)_ = 0.78, *p* = 0.45; effect of day: *t*_(124)_ = 2.72, *p* = 7.42 × 10^−3^; group × day interaction: *t*_(124)_ = −1.54, *p* = 0.13. Individual rat data are shown in Extended Data Figure 4-1. ***I***, Moving average of hand orientation at reach end within individual days for learners (green) and non-learners (pink). ***J***, Average hand orientation MRL at reach end. Linear mixed model: effect of group: *t*_(61)_ = 0.80, *p* = 0.43; effect of day: *t*_(124)_ = 1.97, *p* = 0.05; group × day interaction: *t*_(124)_ = −0.98, *p* = 0.33. Individual rat data are shown in Extended Data Figure 4-1. ***K***, Digit flexion is the angle (δ) between a line joining the second MCP joint and hand dorsum; and a line joining the second MCP joint and the tip of digit 2. ***L***, Average digit flexion at reach end. Linear mixed model: effect of group: *t*_(16)_ = −0.68, *p* = 0.51; effect of day: *t*_(124)_ = −1.49, *p* = 0.14; group × day interaction: *t*_(124)_ = 0.98, *p* = 0.33. Individual rat data are shown in Extended Data Figure 4-1. ***M***, Moving average of digit flexion at reach end within individual days for learners (green) and non-learners (pink). ***N***, Average digit flexion MRL at reach end. Linear mixed model: effect of group: *t*_(26)_ = −1.00, *p* = 0.33; effect of day: *t*_(124)_ = 1.02, *p* = 0.31; group × day interaction: *t*_(124)_ = −0.01, *p* = 0.99. Individual rat data are shown in Extended Data Figure 4-1. Error bars in ***A***, ***D***, ***F***, ***H***, ***J***, ***L***, ***N*** and shaded areas in ***B***, ***E***, ***I***, ***M*** represent SEM; **p < 0.01 and ***p < 0.001 for the day term in the linear mixed model in ***A***, ***H***; #*p* < 0.05 for the group × day interaction in ***A***. Data and code to generate this figure are contained in Extended Data 1, 2.

“Digit flexion” was calculated as the angle between a line connecting the second metacarpophalangeal (MCP) joint and the second digit tip; and a line connecting the hand dorsum and the second MCP joint (Fig. 4*K*). “Aperture” was calculated as the Euclidean distance between the tips of the first and fourth digits (in frames for which both were visible or could be estimated based on epipolar geometry; Fig. 4*C*). “Orientation” was calculated as the angle between a line connecting the first and fourth digits and a horizontal line in the direct camera view (for left-handed rats, orientation was calculated using the negated x-values to compare with right-pawed rats; Fig. 4*G*). Reach velocity was calculated as the Euclidean distance between the dorsum of the reaching hand in consecutive frames divided by the interframe interval (1/300 s). Reach duration was calculated as the number of frames between when the hand first passes through the reaching slot and reach end (defined above), divided by 300 fps (video frame rate).

10.1523/ENEURO.0153-21.2021.f4-1 Extended Data Figure 4-1 Individual rat fine digit kinematics data. ***A***, Average digit 2 trajectory variability of the reach component for individual rats represented as the mean distance from the average trajectory. ***B***, Average digit 2 trajectory variability of the grasp component for individual rats represented as the mean distance from the average trajectory. ***C***, Average aperture at reach end (mm) for individual rats. ***D***, Average aperture variance at reach end for individual rats. ***E***, Average hand orientation (degrees) at reach end for individual rats. ***F***, Average hand orientation MRL at reach end for individual rats. ***G***, Average digit flexion (degrees) at reach end for individual rats. ***H***, Average digit flexion MRL at reach end for individual rats. Data and code to generate this figure are contained in Extended Data 1, 2. Download Figure 4-1, TIF file.

The 3D trajectories of the hand and digit tips were interpolated across 100 evenly spaced points from reach start to reach end or from grasp start to grasp end using the function *interparc* (version 1.3.0.0 from the MATLAB file exchange) with the piecewise cubic Hermite polynomials (pchip) method. This created evenly-spaced trajectories that could be analyzed independently of reach velocity. In other words, slow and fast reaches tracing the same trajectory have matched corresponding points in the interpolated trajectories. Variability of hand and digit trajectories within each session was calculated as the mean distance of each interpolated trial trajectory from the average interpolated session trajectory (Figs. 2*C*,*D*, 4*A*,*B*):

$$\bar{D}=\left[\sum^{}\frac{\sum_{i=1}^{100}(\left|X_{i}-\bar{X_{i}}\right|)}{100}\right]/n,$$

where $\bar{D}$ is the mean distance from the mean trajectory, the ***X_i_*** are the *ith* 3D points along each interpolated trial trajectory, the ${\bar{X}}_{i}$ are the *ith* 3D points in the interpolated session trajectory, and *n* is the number of trials.

To quantify hand and digit 3D endpoint position variability, we used the generalized variance, which provides a single measure of the dispersion of points in multiple dimensions (Figs. 3*D*). Covariance matrices were calculated for the 3D paw/digit endpoints from each trial in each session. The determinant of these covariance matrices defines the generalized variance for reach endpoints.

To determine whether differences in endpoint position variability between the hand and digit tips were because of inconsistencies in DeepLabCut labeling, we compared the number of frames from reach start to grasp end with mislabeled hand and digit tip points on training days 1 and 10 (Fig. 3*E*). Points were first evaluated as correctly or incorrectly labeled based on confidence values generated by DeepLabCut. Points assigned confidence values above 0.97 were deemed correctly labeled, while points assigned confidence values below 0.85 were deemed mislabeled. Points with confidence values between 0.85 and 0.97 were evaluated based on the distance they moved between consecutive frames. If the distance moved between frames surpassed 50 pixels, the point was deemed mislabeled. Importantly, these criteria were used throughout the kinematic analysis so that invalid points were not included in the reconstructed 3D trajectories.

#### Within-session kinematics

To assess how reach kinematics (i.e., aperture, paw orientation, and digit flexion) changed within individual sessions, moving averages were calculated across blocks of 10 trials. To average these data across rats, the last value calculated in each session was carried forward until sessions had the same number of trials. For example, if rat A performed 35 trials and rat B performed 40 trials, the last value calculated for rat A (the average of trials 26–35) would be carried forward as the average of “trials” 27–36, 28–37, … 31–40 so that rats A and B would have the same number of trials. This avoided sudden changes in the average caused by rats performing different numbers of trials (Fig. 4*E*,*I*,*M*). Within-session changes in trajectory variability were calculated as described above, (see Processing reach kinematics) but average trajectories in moving blocks of 10 trials were used as the reference, rather than the average of all trials in a session (Figs. 2*D*, 4*B*).

#### Analysis of reach-to-grasp coordination

**Figure 5.**
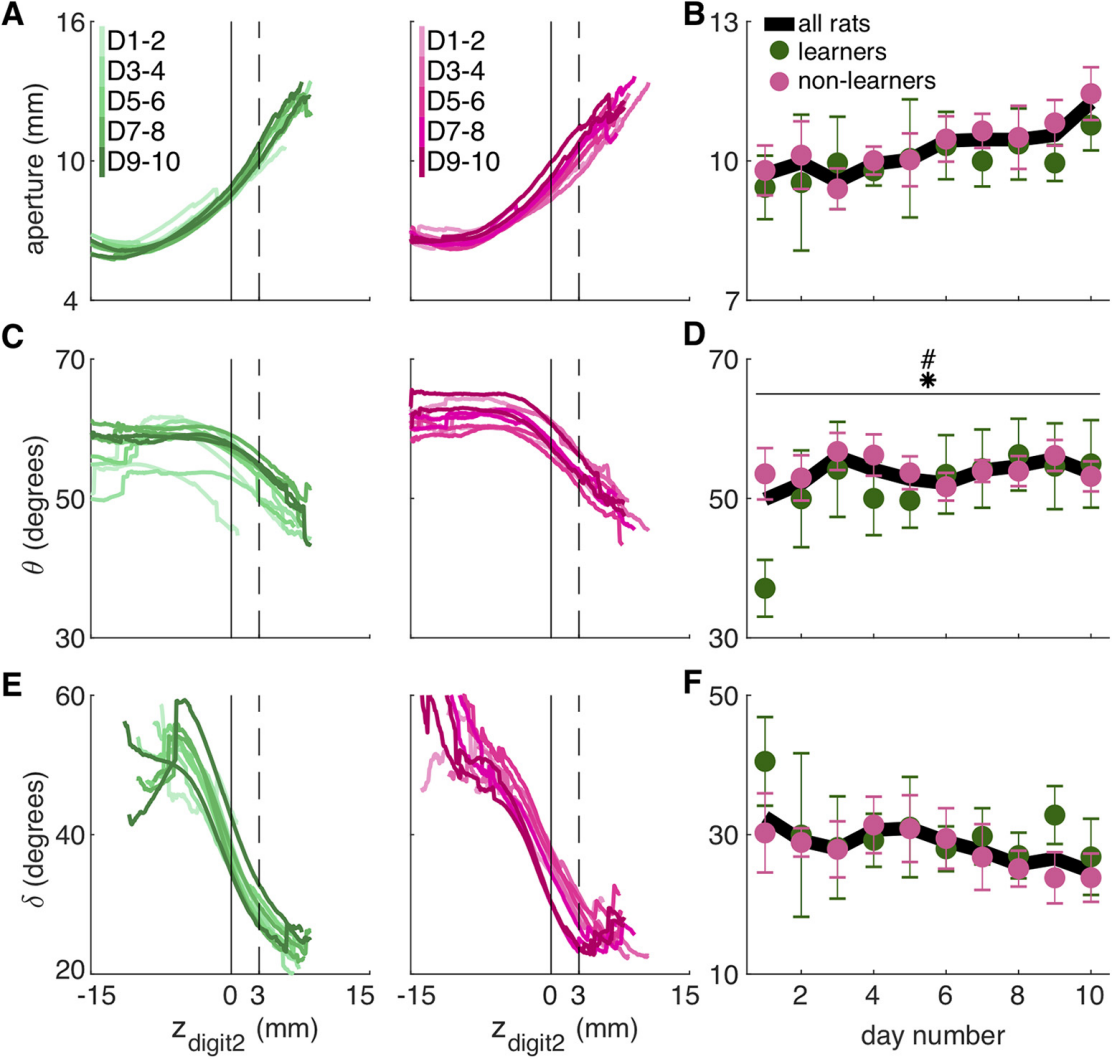
Coordination between digit movements and hand advancement improves with training. ***A***, Mean aperture as a function of hand advancement (z_digit2_, pellet at z_digit2_ = 0) across 10 training days for learners (green) and non-learners (pink). D1–2, D3–4, … represent days 1–2, days 3–4, etc. Dashed line indicates the z_digit2_ coordinate (+3 mm) where data are sampled in ***B***. ***B***, Average grasp aperture at the z_digit2_ coordinate (+3 mm) indicated by the dashed line in ***A*** as a function of day number. Linear mixed model: effect of group: *t*_(30)_ = −0.53, *p* = 0.60; effect of day: *t*_(111)_ = −0.17, *p* = 0.86; group × day interaction: *t*_(111)_ = 1.35, *p* = 0.18. Extended Data Figure 5-1 shows individual rat data. ***C***, Mean hand orientation (θ) as a function of hand advancement across all days for learners (green) and non-learners (pink). Dashed line indicates the z_digit2_ coordinate (+3 mm) where data are sampled in ***D***. ***D***, Average hand orientation (θ) at the z_digit2_ coordinate (+3 mm) indicated by the dashed lines in ***C*** across days. Linear mixed model: effect of group: *t*_(18)_ = 1.11, *p* = 0.28; effect of day: *t*_(111)_ = 2.20, *p* = 0.03; group × day interaction: *t*_(111)_ = −2.08, *p* = 0.04. Extended Data Figure 5-1 shows individual rat data. ***E***, Mean digit flexion (δ) as a function of hand advancement for learners (green) and non-learners (pink). Dashed line indicates the z_digit2_ coordinate (+3 mm) where data are sampled in ***F***. ***F***, Average digit flexion (δ) at the z_digit2_ coordinate (+3 mm) indicated by the dashed lines in ***E*** across days. Linear mixed model: effect of group: *t*_(19)_ = −0.18, *p* = 0.86; effect of day: *t*_(111)_ = −0.05, *p* = 0.96; group × day interaction: *t*_(111)_ = −0.52, *p* = 0.60. Extended Data Figure 5-1 shows individual rat data. Error bars in ***B***, ***D***, ***F*** represent SEM; *p < 0.05 for the day term and #*p* < 0.05 for the group × day interaction in the linear mixed model in ***D***. Data and code to generate this figure are contained in Extended Data 1, 2.

To monitor aperture, hand orientation, and digit flexion as a function of the *z*-coordinate of the tip of the second digit (z_digit2_; Fig. 5), the first reach of each trial was isolated. The 3D trajectory of each digit tip for the initial reach was interpolated using piecewise cubic Hermite polynomials (*pchip* in MATLAB) so that the 3D location of each digit was estimated for z_digit2_ = −20.0, −19.9, −19.8… +14.9, +15.0 mm from the pellet (positive numbers are past the pellet, negative numbers as the hand approaches the pellet). This allowed us to average aperture, orientation, and digit flexion as a function of hand advancement (assessed by z_digit2_). Points at z_digit2_ values missing from shorter reaches were excluded from the average. To compare the evolution of aperture, hand orientation, and digit flexion across the 10 training sessions, we compared digit aperture, paw orientation, and digit flexion at z_digit2_ = +3 mm (Fig. 5*B*,*D*,*F*).

10.1523/ENEURO.0153-21.2021.f5-1 Extended Data Figure 5-1 Digit and forelimb coordination individual rat data. ***A***, Average grasp aperture at the z_digit2_ coordinate (+3 mm) indicated by the dashed line in Figure 5A as a function of day number for individual rats (left learners; right non-learners). ***B***, Average hand orientation (degrees) at the z_digit2_ coordinate (+3 mm) indicated by the dashed line in Figure 5C as a function of day number for individual rats. ***C***, Average digit flexion (degrees) at the z_digit2_ coordinate (+3 mm) indicated by the dashed line in Figure 5*E* as a function of day number for individual rats. Data and code to generate this figure are contained in Extended Data 1, 2. Download Figure 5-1, TIF file.

### Statistics

**Figure 6.**
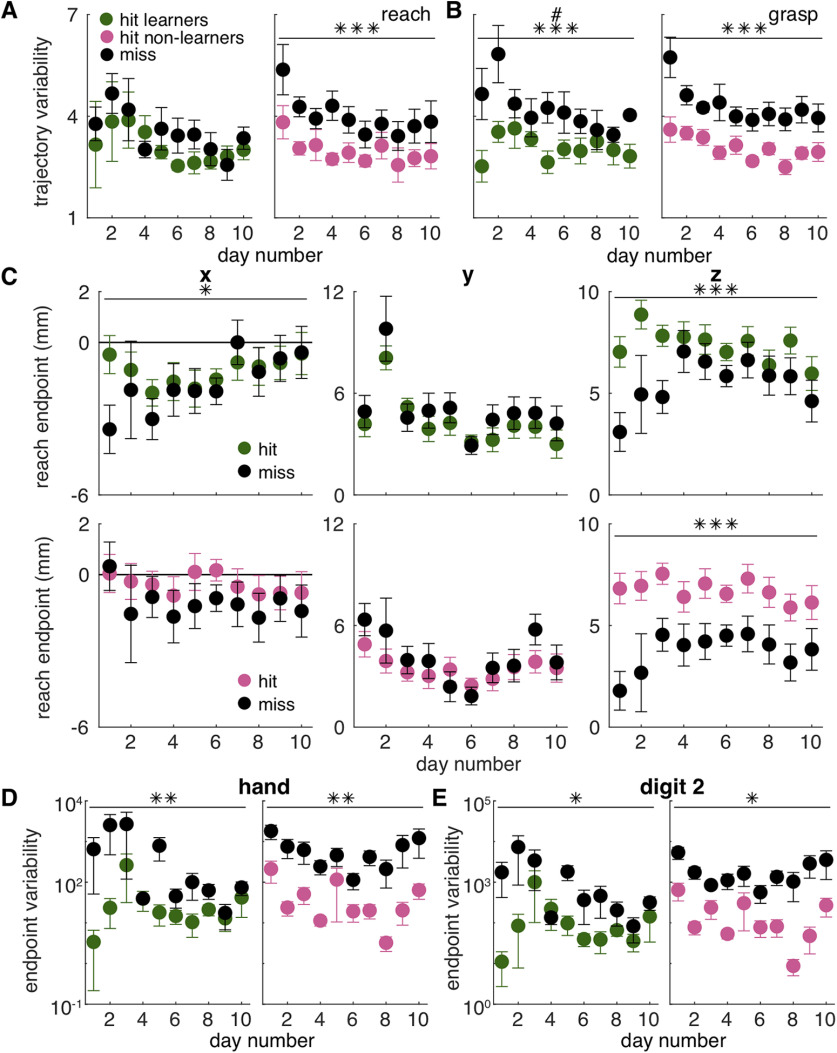
Kinematic measures separated by reach success or failure. ***A***, Average hand trajectory variability (mean distance from the average trajectory, mm) for the reach component in learners (green) and non-learners (pink). Trajectory variability was significantly higher for failed reaches (“miss,” black) than successful reaches (“hit,” green/pink) for non-learners, but not for learners. Linear mixed model (learners): effect of outcome: *t*_(72)_ = 1.26, *p* = 0.21; outcome × day interaction: *t*_(72)_ = −0.46, *p* = 0.64. Linear mixed model (non-learners): effect of outcome: *t*_(183)_ = 4.44, *p* = 1.56 × 10^−5^; outcome × day interaction: *t*_(183)_ = −1.25, *p* = 0.21. Individual rat data are shown in Extended Data Figure 6-1. ***B***, Average hand trajectory variability (mean distance from the average trajectory, mm) for the grasp component in learners (green) and non-learners (pink). Trajectory variability was significantly higher for failed reaches (“miss,” black) than successful reaches (“hit,” green/pink) for both groups. Linear mixed model (learners): effect of outcome: *t*_(72)_ = 4.77, *p* = 9.34 × 10^−6^; outcome × day interaction: *t*_(72)_ = −2.23, *p* = 0.03. Linear mixed model (non-learners): effect of outcome: *t*_(183)_ = 5.58, *p* = 8.47 × 10^−8^; outcome × day interaction: *t*_(183)_ = −1.02, *p* = 0.31. Individual rat data are shown in Extended Data Figure 6-1. ***C***, Average reach endpoint of digit 2 in the *X*, *Y*, and *Z* directions for learners (top) and non-learners (bottom). Reach endpoint in the *X* direction was significantly different for successful (green/pink) versus failed (black) reaches for learners but not non-learners. Linear mixed model (learners): effect of outcome: *t*_(72)_ = −2.31, *p* = 0.02; outcome × day interaction: *t*_(72)_ = 1.94, *p* = 0.06. Linear mixed model (non-learners): effect of outcome: *t*_(185)_ = −1.53, *p* = 0.13; outcome × day interaction: *t*_(185)_ = −0.03, *p* = 0.97. Reach endpoint in the *Y* direction did not differ between successful and failed reaches for either group. Linear mixed model (learners): effect of outcome: *t*_(72)_ = −0.95, *p* = 0.35; outcome × day interaction: *t*_(72)_ = 0.23, *p* = 0.82. Linear mixed model (non-learners): effect of outcome: *t*_(185)_ = −1.49, *p* = 0.14; outcome × day interaction: *t*_(185)_ = 0.77, *p* = 0.44. Failed reaches were significantly shorter (*Z* direction) than successful reaches for both groups, although the *Z* endpoint did not differ between successful and failed reaches in later training days for learners. Linear mixed model (learners): effect of outcome: *t*_(72)_ = 3.52, *p* = 7.48 × 10^−4^; outcome × day interaction: *t*_(72)_ = −1.86, *p* = 0.07. Linear mixed model (non-learners): effect of outcome: *t*_(185)_ = 4.94, *p* = 1.76 × 10^−6^; outcome × day interaction: *t*_(185)_ = −1.53, *p* = 0.13. Individual rat data are shown in Extended Data Figure 6-2. ***D***, Average determinant of the covariance matrix (generalized variance) of reach endpoints for the hand across days for learners (green) and non-learners (pink). There was significantly greater variability in reach endpoint for failed (black) reaches than successful (green/pink) reaches for both groups, although endpoint variability did not differ between successful and failed reaches in later training days for learners. Linear mixed model (learners): effect of outcome: *t*_(72)_ = 2.67, *p* = 9.26 × 10^−3^; outcome × day interaction: *t*_(72)_ = −1.95, *p* = 0.05; non-learners: effect of outcome: *t*_(185)_ = 2.63, *p* = 9.34 × 10^−3^; outcome × day interaction: *t*_(185)_ = −0.67, *p* = 0.50. Individual rat data are shown in Extended Data Figure 6-2. ***E***, Average determinant of the covariance matrix (generalized variance) of reach endpoints for digit 2 across days for learners (green) and non-learners (pink). There was significantly greater variability in reach endpoint for failed (black) reaches than successful (green/pink) reaches for both groups, although endpoint variability did not differ between successful and failed reaches in later training days for learners. Linear mixed model (learners): effect of outcome: *t*_(72)_ = 2.64, *p* = 0.01; outcome × day interaction: *t*_(72)_ = −1.92, *p* = 0.06; non-learners: effect of outcome: *t*_(185)_ = 2.60, *p* = 0.01; outcome × day interaction: *t*_(185)_ = −0.25, *p* = 0.80. Individual rat data are shown in Extended Data Figure 6-2. Error bars in ***A–E*** represent SEM; *p < 0.05 for the outcome term in the linear mixed model in ***C***, ***E***, **p < 0.01 for the outcome term in the linear mixed model in ***D***, ***p < 0.001 for the outcome term in the linear mixed model in ***A–C***; #*p* < 0.05 for the outcome × day interaction in the linear mixed model in ***B***. Summaries of key metrics for every trial performed by each rat, separated by trial outcomes, are shown in Extended Data Figures 6-3, 6-4, 6-5, 6-6, 6-7, 6-8, 6-9, 6-10, 6-11, 6-12, 6-13, 6-14, 6-15, 6-16. Data and code to generate this figure are contained in Extended Data 1, 2.

See Table 1 for a detailed description of statistical analyses. For Figures 1-5, linear mixed-effects models were used to evaluate whether performance outcomes and kinematics changed over training days and/or differed between learners and non-learners. We implemented linear mixed-effects models (using R *lmer*) with random intercepts/effects for each rat and fixed effects of training day and group (interaction). Linear mixed-effects models included averages for all 10 training days for all rats. To assess whether reach kinematics differed between successful and failed reaches, we implemented linear mixed-effects models (using R *lmer*) with random intercepts/effects for each rat and fixed interaction effects of training day and outcome. Separate linear mixed-effects models were run for each group (Fig. 6).

**Table 1:** Statistical table

Figure	Data structure	Type of test	Sample size	Statistical data
Fig. 1*B*	Effect of group on number of trials Effect of day on number of trials Group × day interaction	Linear mixed model	*n* = 14	*t *=* *0.49, df = 28.48, *p *=* *0.63 *t *=* *124, df = 3.58, *p *=* *4.88 × 10^−4^ *t *=* *124, df = −0.47, *p *=* *0.64
Fig. 1*C*	Effect of group on first reach success rate Effect of day on first reach success rate Group × day interaction	Linear mixed model	*n* = 14	*t *=* *0.442, df = 25, *p *=* *0.66 *t *=* *5.55, df = 123, *p *=* *1.70 × 10^−7^ *t* = −4.78, df = 123, *p *=* *4.98 × 10^−6^
Extended Data Fig. 1-1*C*	Effect of group on number of reach attempts Effect of day on number of reach attempts Group × day interaction	Linear mixed model	*n* = 14	*t *=* *0.81, df = 19, *p *=* *0.43 *t* = −4.12, df = 124, *p *=* *6.88 × 10^−5^ *t *=* *1.37, df = 124, *p *=* *0.17
Extended Data Fig. 1-1*D*	Effect of day on performance outcomes, learners Effect of day on performance outcomes, non-learners	Linear mixed model	*n* = 4	No pellet: *t* = −0.11, df = 35, *p *=* *0.91 First success: *t *=* *4.96, df = 35, *p *=* *1.83 × 10^−5^ Multiple success: *t* = −2.53, df = 35, *p *=* *0.02 Drop in box: *t *=* *0.42, df = 35, *p *=* *0.68 Pellet knock off: *t* = −4.59, df = 35, *p* =5.46 × 10^−5^ Tongue: *t *=* *0, df = 35, *p *=* *1 Trigger error: *t* = −0.52, df = 38, *p *=* *0.61 Pellet remained: *t* = −0.78, df = 38, *p *=* *0.44 Non-preferred hand: *t* = −0.87, df = 38, *p *=* *0.39 Tongue and hand: *t *=* *0, df = 38, *p *=* *1 Hand through slot: *t* = −0.78, df = 38, *p *=* *0.39 No pellet: *t* = −0.62, df = 98, *p *=* *0.54 First success: *t *=* *0.51, df = 88, *p *=* *0.61 Multiple success: *t* = −0.79, df = 89, *p *=* *0.43 Drop in box: *t *=* *0.36, df = 89, *p *=* *0.72 Pellet knock off: *t *=* *0.49, df = 89, *p *=* *0.62 Tongue: *t *=* *0, df = 98, *p *=* *1 Trigger error: *t* = −1.37, df = 98, *p *=* *0.18 Pellet remained: *t* = −0.95, df = 98, *p *=* *0.35 Non-preferred hand: *t* = −0.87, df = 98, *p *=* *0.39 Tongue and hand: *t *=* *0, df = 98, *p *=* *1 Hand through slot: *t *=* *0.01, df = 98, *p *=* *0.99
Fig. 2*C*	Effect of group on paw trajectory variability Effect of day on paw trajectory variability Group × day interaction	Linear mixed model	*n* = 14	Reach: *t *=* *0.53, df = 23, *p *=* *0.60 Grasp: *t* = −0.08, df = 26, *p *=* *0.94 Reach: *t* = −1.85, df = 124, *p *=* *0.07 Grasp: *t* = −2.75, df = 124, *p *=* *6.91 × 10^−3^ Reach: *t *=* *0.37, df = 124, *p *=* *0.71 Grasp: *t *=* *1.08, df = 124, *p *=* *0.28
Fig. 2*D*	Negative correlation between trajectory variability and success rate, learners Negative correlation between trajectory variability and success rate, non-learners	Linear correlation	*n* = 4 *n* = 10	Session 1: *r *=* *0.73, *p *=* *5.33 × 10^−6^ Session 2: *r* = −0.55, *p *=* *1.70 × 10^3^ Session 3: *r* = −0.03, *p *=* *0.86 Session 4: *r* = −0.68, *p *=* *4.25 × 10^−5^ Session 5: *r* = −0.53, *p *=* *2.50 × 10^−3^ Session 6: *r* = −0.82, *p *=* *2.16 × 10^−8^ Session 7: *r *=* *0.15, *p *=* *0.44 Session 8: *r* = −0.70, *p *=* *1.51 × 10^−5^ Session 9: *r *=* *0.39, *p *=* *0.03 Session 10: *r* = −0.24, *p *=* *0.20 Session 1: *r* = −0.88, *p *=* *1.78 × 10^−10^ Session 2: *r* = −0.02, *p *=* *0.91 Session 3: *r *=* *0.02, *p *=* *0.91 Session 4: *r* = −0.67, *p *=* *4.33 × 10^−5^ Session 5: *r* = −0.33, *p *=* *0.08 Session 6: *r* = −0.38, *p *=* *0.04 Session 7: *r *=* *0.67, *p *=* *4.33 × 10^−5^ Session 8: *r* = −0.42, *p *=* *0.02 Session 9: *r* = −0.07, *p *=* *0.73 Session 10: *r* = −0.83, *p *=* *1.53 × 10^−8^
Fig. 2*E*	Effect of group on reach duration Effect of day on reach duration Group × day interaction	Linear mixed model	*n* = 14	*t *=* *0.29, df = 21, *p *=* *0.78 *t* = −1.03, df = 124, *p *=* *0.31 *t* = −0.11, df = 124, *p *=* *0.91
Fig. 2*F*	Effect of group on reach velocity Effect of day on reach duration Group × day interaction	Linear mixed model	*n* = 14	*t *=* *0.16, df = 15, *p *=* *0.87 *t* = −0.07, df = 124, *p *=* *0.94 *t *=* *1.02, df = 124, *p *=* *0.31
Fig. 3*B*	Effect of group on reach endpoint Effect of day on reach endpoint Group × day interaction	Linear mixed model	*n* = 14	Paw *x*: *t* =1.30, df = 18, *p *=* *0.21 Paw *y*: *t* = −0.94, df = 18, *p *=* *0.36 Paw *z*: *t* = −0.38, df = 124, *p *=* *0.71 Digit 2 ×: *t *=* *1.80, df = 17, *p *=* *0.09 Digit 2 year: *t* = −0.90, df = 19, *p *=* *0.38 Digit 2 *z*: *t* = −0.63, df = 19, *p *=* *0.53 Paw *x*: *t *=* *2.13, df = 124, *p *=* *0.04 Paw *y*: *t* = −2.45, df = 124, *p *=* *0.02 Paw *z*: *t *=* *1.73, df = 124, *p *=* *0.09 Digit 2 ×: *t *=* *3.12, df = 124, *p *=* *2.25 × 10^3^ Digit 2 year: *t* = −1.80, df = 124, *p *=* *0.07 Digit 2 *z*: *t *=* *0.99, df = 124, *p *=* *0.33 Paw *x*: *t* = −2.40, df = 124, *p *=* *0.02 Paw *y*: *t *=* *1.45, df = 124, *p *=* *0.15 Paw *z*: *t* = −1.23, df = 124, *p *=* *0.22 Digit 2 ×: *t* = −3.16, df = 124, *p *=* *1.97 × 10^3^ Digit 2 year: *t *=* *1.16, df = 124, *p *=* *0.25 Digit 2 *z*: *t* = −0.58, df = 124, *p *=* *0.57
Fig. 3*D*	Effect of group on endpoint variability, raw data Effect of day on endpoint variability, raw data Group × day interaction, raw data Effect of group on endpoint variability, subtracted position Effect of day on endpoint variability, subtracted position Group × day interaction, subtracted position	Linear mixed model	*n* = 14	Hand: *t* = −0.95, df = 51, *p *=* *0.35 Digit 1: *t* = −0.71, df = 56, *p *=* *0.48 Digit 2: *t* = −0.86, df = 45, *p *=* *0.39 Digit 3: *t* = −0.42, df = 49, *p *=* *0.67 Digit 4: *t* = −0.29, df = 48, *p *=* *0.78 Hand: *t* = −2.27, df = 124, *p *=* *0.02 Digit 1: *t* = −1.97, df = 124, *p *=* *0.05 Digit 2: *t* = −2.25, df = 124, *p *=* *0.03 Digit 3: *t* = −1.85, df = 124, *p *=* *0.07 Digit 4: *t* = −1.78, df = 124, *p *=* *0.08 Hand: *t *=* *1.39, df = 124, *p *=* *0.17 Digit 1: *t *=* *1.15, df = 124, *p *=* *0.25 Digit 2: *t *=* *1.42, df = 124, *p *=* *0.16 Digit 3: *t *=* *0.90, df = 124, *p *=* *0.37 Digit 4: *t *=* *0.89, df = 124, *p *=* *0.37 Digit 1: *t *=* *0.13, df = 78, *p *=* *0.89 Digit 2: *t* = −1.61, df = 112, *p *=* *0.11 Digit 3: *t* = −0.75, df = 136, *p *=* *0.45 Digit 4: *t *=* *0.62, df = 107, *p *=* *0.54 Digit 1: *t* = −0.87, df = 124, *p *=* *0.39 Digit 2: *t* = −2.23, df = 124, *p *=* *0.03 Digit 3: *t* = −1.64, df = 136, *p *=* *0.10 Digit 4: *t* = −0.67, df = 124, *p *=* *0.51 Digit 1: *t *=* *0.64, df = 124, *p *=* *0.52 Digit 2: *t *=* *1.79, df = 124, *p *=* *0.08 Digit 3: *t *=* *1.21, df = 136, *p *=* *0.23 Digit 4: *t *=* *0.03, df = 124, *p *=* *0.98
Fig. 3*E*	Effect of part on % mislabeled frames Effect of day on % mislabeled frames Part × day interaction	Linear mixed model	*n* = 14	*t* = −0.05, df = 123, *p *=* *0.96 *t *=* *0.98, df = 123, *p *=* *0.33 Hand: *t* = −0.79, df = 117, *p *=* *0.43 Digit 1: *t *=* *1.07, df = 117, *p *=* *0.29 Digit 2: *t *=* *0.91, df = 117, *p *=* *0.37 Digit 3: *t *=* *1.82, df = 117, *p *=* *0.07 Digit 4: *t *=* *2.71, df = 117, *p *=* *7.74 × 10^−3^
Fig. 4*A*	Effect of group on digit 2 trajectory variability Effect of day on digit 2 trajectory variability Group × day interaction	Linear mixed model	*n* = 14	Reach: *t *=* *1.39, df = 23, *p *=* *0.18 Grasp: *t* = −0.60, df = 27, *p *=* *0.55 Reach: *t* = −1.69, df = 124, *p *=* *0.09 Grasp: *t* = −3.92, df = 124, *p *=* *1.46 × 10^−4^ Reach: *t* = −0.01, df = 124, *p *=* *0.99 Grasp: *t *=* *2.29, df = 124, *p *=* *0.02
Fig. 4*D*	Effect of group on aperture Effect of day on aperture Group × day interaction	Linear mixed model	*n* = 14	*t* = −0.81, df = 15, *p *=* *0.43 *t *=* *0.97, df = 124, *p *=* *0.34 *t *=* *0.36, df = 124, *p *=* *0.72
Fig. 4*F*	Effect of group on aperture variance Effect of day on aperture variance Group × day interaction	Linear mixed model	*n* = 14	*t *=* *0.96, df = 22, *p *=* *0.35 *t* = −1.53, df = 124, *p *=* *0.13 *t *=* *0.90, df = 124, *p *=* *0.37
Fig. 4*H*	Effect of group on hand orientation Effect of day on hand orientation Group × day interaction	Linear mixed model	*n* = 14	*t *=* *0.78, df = 15, *p *=* *0.45 *t *=* *2.72, df = 124, *p *=* *7.42 × 10^−3^ *t* = −1.54, df = 124, *p *=* *0.13
Fig. 4*J*	Effect of group on orientation variance Effect of day on orientation variance Group × day interaction	Linear mixed model	*n* = 14	*t *=* *0.80, df = 61, *p *=* *0.43 *t *=* *1.97, df = 124, *p *=* *0.05 *t* = −0.98, df = 124, *p *=* *0.33
Fig. 4*L*	Effect of group on digit flexion Effect of day on digit flexion Group × day interaction	Linear mixed model	*n* = 14	*t* −0.68, df = 16, *p *=* *0.51 *t* = −1.49, df = 124, *p *=* *0.14 *t *=* *0.98, df = 124, *p *=* *0.33
Fig. 4*N*	Effect of group on flexion variance Effect of day on flexion variance Group × day interaction	Linear mixed model	*n* = 14	*t* = −1.00, df = 26, *p *=* *0.33 *t *=* *1.02, df = 124, *p *=* *0.31 *t* = −0.01, df = 124, *p *=* *0.99
Fig. 5*B*	Effect of group on aperture Effect of day on aperture Group × day interaction	Linear mixed model	*n* = 14	*t* = −0.53, df = 30, *p *=* *0.60 *t* = −0.17, df = 111, *p *=* *0.86 *t *=* *1.35, df = 111, *p *=* *0.18
Fig. 5*D*	Effect of group on orientation Effect of day on orientation Group × day interaction	Linear mixed model	*n* = 14	*t *=* *1.11, df = 18, *p *=* *0.28 *t *=* *2.20, df = 111, *p *=* *0.03 *t* = −2.08, df = 111, *p *=* *0.04
Fig. 5*F*	Effect of group on flexion Effect of day on flexion Group × day interaction	Linear mixed model	*n* = 14	*t* = −0.184, df = 19, *p *=* *0.86 *t* = −0.05, df = 111, *p *=* *0.96 *t* = −0.52, df = 111, *p *=* *0.60
Fig. 6*A*	Effect of outcome on reach trajectory variability Effect of day on reach trajectory variability Outcome × day interaction	Linear mixed model	*n* = 4 *n* = 10 *n* = 4 *n* = 10 *n* = 4 *n* = 10	Learners: *t *=* *1.26, df = 72, *p *=* *0.21 Non-learners: *t *=* *4.44, df = 183, *p *=* *1.56 × 10^−5^ Learners: *t* = −1.84, df = 72, *p *=* *0.07 Non-learners: *t* = −2.01, df = 183, *p *=* *0.046 Learners: *t* = −0.46, df = 72, *p *=* *0.64 Non-learners: *t* = −1.25, df = 183, *p *=* *0.21
Fig. 6*B*	Effect of outcome on grasp trajectory variability Effect of day on grasp trajectory variability Outcome × day interaction	Linear mixed model	*n* = 4 *n* = 10 *n* = 4 *n* = 10 *n* = 4 *n* = 10	Learners: *t *=* *4.77, df = 72, *p *=* *9.34 × 10^−6^ Non-learners: *t *=* *5.58, df = 183, *p *=* *8.47 × 10^−8^ Learners: *t* = −0.30, df = 72, *p *=* *0.77 Non-learners: *t* = −2.88, df = 183, *p *=* *4.42 × 10^3^ Learners: *t* = −2.23, df = 72, *p *=* *0.03 Non-learners: *t* = −1.02, df = 183, *p *=* *0.31
Fig. 6*C*	Effect of outcome on reach endpoint Effect of day on reach endpoint Outcome × day interaction	Linear mixed model	*n* = 4 *n* = 10 *n* = 4 *n* = 10 *n* = 4	Learners *X*: *t* = −2.31, df = 72, *p *=* *0.02 Learners *Y*: *t* = −0.95, df = 72, *p *=* *0.35 Learners *Z*: *t *=* *3.52, df = 72, *p* =7.48 × 10^−4^ Non-learners *X*: *t* = −1.53, df = 185, *p *=* *0.13 Non-learners *Y*: *t* = −1.49, df = 185, *p *=* *0.14 Non-learners *Z*: *t *=* *4.94, df = 185, *p *=* *1.76 × 10^−6^ Learners *X*: *t *=* *0.95, df = 72, *p *=* *0.34 Learners *Y*: *t *=* *1.54, df = 72, *p *=* *0.13 Learners *Z*: *t *=* *1.31, df = 72, *p *=* *0.20 Non-learners *X*: *t* = −1.46, df = 185, *p *=* *0.15 Non-learners *Y*: *t *=* *0.59, df = 185, *p *=* *0.56 Non-learners *Z*: *t *=* *0.76, df = 185, *p *=* *0.45 Learners *X*: *t *=* *1.94, df = 72, *p *=* *0.06 Learners *Y*: *t *=* *0.23, df = 72, *p *=* *0.82 Learners *Z*: *t* = −1.86, df = 72, *p *=* *0.07 Non-learners *X*: *t* = −0.03, df = 185, *p *=* *0.97 Non-learners *Y*: *t *=* *0.77, df = 185, *p *=* *0.44 Non-learners *Z*: *t* = −1.53, df = 185, *p *=* *0.13
Fig. 6*D*	Effect of outcome on hand endpoint variability Effect of day on hand endpoint variability Outcome × day interaction	Linear mixed model	*n* = 4 *n* = 10 *n* = 4 *n* = 10 *n* = 4 *n* = 10	Learners: *t *=* *2.67, df = 72, *p *=* *9.26 × 10^−3^ Non-learners: *t *=* *2.63, df = 185, *p *=* *9.34 × 10^−3^ Learners: *t* = −0.06, df = 72, *p *=* *0.96 Non-learners: *t* = −0.26, df = 185, *p *=* *0.80 Learners: *t* = −1.95, df = 72, *p *=* *0.05 Non-learners: *t* = −0.67, df = 185, *p *=* *0.50
Fig. 6*E*	Effect of outcome on digit 2 endpoint variability Effect of day on digit 2 endpoint variability Outcome × day interaction	Linear mixed model	*n* = 4 *n* = 10 *n* = 4 *n* = 10 *n* = 4 *n* = 10	Learners: *t *=* *2.64, df = 72, *p *=* *0.01 Non-learners: *t *=* *2.60, df = 185, *p *=* *0.01 Learners: *t* = −0.14, df = 72, *p *=* *0.89 Non-learners: *t* = −0.22, df = 185, *p *=* *0.83 Learners: *t* = −1.92, df = 72, *p *=* *0.06 Non-learners: *t* = −0.25, df = 185, *p *=* *0.80

10.1523/ENEURO.0153-21.2021.f6-1 Extended Data Figure 6-1 Trajectory variability by outcome for individual rats. ***A***, Average hand trajectory variability of the reach component for successful reaches (“hits,” left column) and unsuccessful reaches (“misses,” right column) for individual learner (left) and non-learner (right) rats. ***B***, Average hand trajectory variability of the grasp component for successful reaches (“hits,” left column) and unsuccessful reaches (“misses,” right column) for individual learner (left) and non-learner (right) rats. Data and code to generate this figure are contained in Extended Data 1, 2. Download Figure 6-1, TIF file.

10.1523/ENEURO.0153-21.2021.f6-2 Extended Data Figure 6-2 Reach endpoint by outcome for individual rats. ***A***, Average reach endpoint (*X* direction) for successful reaches (“hits”, left column) and unsuccessful reaches (“misses”, right column) for individual learner (left) and non-learner (right) rats. ***B***, Average reach endpoint (*Y* direction) for successful reaches (“hits”, left column) and unsuccessful reaches (“misses”, right column) for individual learner (left) and non-learner (right) rats. ***C***, Average reach endpoint (*Z* direction) for successful reaches (“hits”, left column) and unsuccessful reaches (“misses”, right column) for individual learner (left) and non-learner (right) rats. ***D***, Average reach endpoint variability of the hand for successful reaches (“hits”, left column) and unsuccessful reaches (“misses”, right column) for individual learner (left) and non-learner (right) rats. ***E***, Average reach endpoint variability of digit 2 for successful reaches (“hits,” left column) and unsuccessful reaches (“misses,” right column) for individual learner (left) and non-learner (right) rats. Data and code to generate this figure are contained in Extended Data 1, 2. Download Figure 6-2, TIF file.

10.1523/ENEURO.0153-21.2021.f6-3 Extended Data Figure 6-3 Kinematics summary sheet for a nonlearner rat. ***A***, Success rate across days. Pink lines indicate first reach success, black lines indicate success on any reach attempt for a single trial. ***B***, Shared control plots illustrating measures of hand shaping at reach end (all data for a single rat that went into Fig. 4*C–N*). The top axes are swarm plots showing aperture at reach end for every trial. Pink dots indicate first-reach success trials, black dots indicate first reach failed trials (i.e., pellet remained, pellet knocked off, or multiple reach success), and gray dots indicate all other trials (e.g., no pellet delivered). Bottom plots show the difference between the mean value on each day and the mean value on day 1. Distributions show the results of a bootstrap resampling procedure with 95% confidence intervals indicated by the solid lines at the left of each distribution. ***C***, Same as ***B*** for the mean distance from the average reach trajectory for each day (top panel shows all hand location data for a single rat that went into Fig. 2*C*, left panel; bottom panel shows all digit 2 location data that went into Fig. 4*A*, left panel). ***D***, Same as ***B***, ***C*** for the reach endpoint analyses. Left column shows reach endpoints in x, y, and z for the hand location (all data for a single rat that went into Fig. 3*B*, left panels). Right column shows reach endpoints for digit 2 with respect to the hand location (all data for a single rat that went into Fig. 3*B*, right panels, except here the hand location was subtracted out). Data and code to generate this figure are contained in Extended Data 1, 2, 3. Download Figure 6-3, TIF file.

10.1523/ENEURO.0153-21.2021.f6-4 Extended Data Figure 6-4 Kinematics summary sheet for a learner rat. ***A***, Success rate across days. Green lines indicate first reach success, black lines indicate success on any reach attempt for a single trial. ***B***, Shared control plots illustrating measures of hand shaping at reach end (all data for a single rat that went into Fig. 4*C–N*). The top axes are swarm plots showing aperture at reach end for every trial. Green dots indicate first-reach success trials, black dots indicate first reach failed trials (i.e., pellet remained, pellet knocked off, or multiple reach success), and gray dots indicate all other trials (e.g., no pellet delivered). Bottom plots show the difference between the mean value on each day and the mean value on day 1. Distributions show the results of a bootstrap resampling procedure with 95% confidence intervals indicated by the solid lines at the left of each distribution. ***C***, Same as ***B*** for the mean distance from the average reach trajectory for each day (top panel shows all hand location data for a single rat that went into Fig. 2*C*, left panel; bottom panel shows all digit 2 location data that went into Fig. 4*A*, left panel). ***D***, Same as ***B***, ***C*** for the reach endpoint analyses. Left column shows reach endpoints in *x*, *y*, and *z* for the hand location (all data for a single rat that went into Fig. 3*B*, left panels). Right column shows reach endpoints for digit 2 with respect to the hand location (all data for a single rat that went into Fig. 3*B*, right panels, except here the hand location was subtracted out). Data and code to generate this figure are contained in Extended Data 1, 2, 3. Download Figure 6-4, TIF file.

10.1523/ENEURO.0153-21.2021.f6-5 Extended Data Figure 6-5 Kinematics summary sheet for a nonlearner rat. ***A***, Success rate across days. Pink lines indicate first reach success, black lines indicate success on any reach attempt for a single trial. ***B***, Shared control plots illustrating measures of hand shaping at reach end (all data for a single rat that went into Fig. 4*C–N*). The top axes are swarm plots showing aperture at reach end for every trial. Pink dots indicate first-reach success trials, black dots indicate first reach failed trials (i.e., pellet remained, pellet knocked off, or multiple reach success), and gray dots indicate all other trials (e.g., no pellet delivered). Bottom plots show the difference between the mean value on each day and the mean value on day 1. Distributions show the results of a bootstrap resampling procedure with 95% confidence intervals indicated by the solid lines at the left of each distribution. ***C***, Same as ***B*** for the mean distance from the average reach trajectory for each day (top panel shows all hand location data for a single rat that went into Fig. 2*C*, left panel; bottom panel shows all digit 2 location data that went into Fig. 4*A*, left panel). ***D***, Same as ***B***, ***C*** for the reach endpoint analyses. Left column shows reach endpoints in *x*, *y*, and *z* for the hand location (all data for a single rat that went into Fig. 3*B*, left panels). Right column shows reach endpoints for digit 2 with respect to the hand location (all data for a single rat that went into Fig. 3*B*, right panels, except here the hand location was subtracted out). Data and code to generate this figure are contained in Extended Data 1, 2, 4. Download Figure 6-5, TIF file.

10.1523/ENEURO.0153-21.2021.f6-6 Extended Data Figure 6-6 Kinematics summary sheet for a nonlearner rat. ***A***, Success rate across days. Pink lines indicate first reach success, black lines indicate success on any reach attempt for a single trial. ***B***, Shared control plots illustrating measures of hand shaping at reach end (all data for a single rat that went into Fig. 4*C–N*). The top axes are swarm plots showing aperture at reach end for every trial. Pink dots indicate first-reach success trials, black dots indicate first reach failed trials (i.e., pellet remained, pellet knocked off, or multiple reach success), and gray dots indicate all other trials (e.g., no pellet delivered). Bottom plots show the difference between the mean value on each day and the mean value on day 1. Distributions show the results of a bootstrap resampling procedure with 95% confidence intervals indicated by the solid lines at the left of each distribution. ***C***, Same as ***B*** for the mean distance from the average reach trajectory for each day (top panel shows all hand location data for a single rat that went into Fig. 2*C*, left panel; bottom panel shows all digit 2 location data that went into Fig. 4*A*, left panel). ***D***, Same as ***B***, ***C*** for the reach endpoint analyses. Left column shows reach endpoints in x, y, and z for the hand location (all data for a single rat that went into Fig. 3*B*, left panels). Right column shows reach endpoints for digit 2 with respect to the hand location (all data for a single rat that went into Fig. 3*B*, right panels, except here the hand location was subtracted out). Data and code to generate this figure are contained in Extended Data 1, 2, 4. Download Figure 6-6, TIF file.

10.1523/ENEURO.0153-21.2021.f6-7 Extended Data Figure 6-7 Kinematics summary sheet for a nonlearner rat. ***A***, Success rate across days. Pink lines indicate first reach success, black lines indicate success on any reach attempt for a single trial. ***B***, Shared control plots illustrating measures of hand shaping at reach end (all data for a single rat that went into Fig. 4*C–N*). The top axes are swarm plots showing aperture at reach end for every trial. Pink dots indicate first-reach success trials, black dots indicate first reach failed trials (i.e., pellet remained, pellet knocked off, or multiple reach success), and gray dots indicate all other trials (e.g., no pellet delivered). Bottom plots show the difference between the mean value on each day and the mean value on day 1. Distributions show the results of a bootstrap resampling procedure with 95% confidence intervals indicated by the solid lines at the left of each distribution. ***C***, Same as ***B*** for the mean distance from the average reach trajectory for each day (top panel shows all hand location data for a single rat that went into Fig. 2*C*, left panel; bottom panel shows all digit 2 location data that went into Fig. 4*A*, left panel). ***D***, Same as ***B***, ***C*** for the reach endpoint analyses. Left column shows reach endpoints in x, y, and z for the hand location (all data for a single rat that went into Fig. 3*B*, left panels). Right column shows reach endpoints for digit 2 with respect to the hand location (all data for a single rat that went into Fig. 3*B*, right panels, except here the hand location was subtracted out). Data and code to generate this figure are contained in Extended Data 1, 2, 5. Download Figure 6-7, TIF file.

10.1523/ENEURO.0153-21.2021.f6-8 Extended Data Figure 6-8 Kinematics summary sheet for a learner rat. ***A***, Success rate across days. Green lines indicate first reach success, black lines indicate success on any reach attempt for a single trial. ***B***, Shared control plots illustrating measures of hand shaping at reach end (all data for a single rat that went into Fig. 4*C–N*). The top axes are swarm plots showing aperture at reach end for every trial. Green dots indicate first-reach success trials, black dots indicate first reach failed trials (i.e., pellet remained, pellet knocked off, or multiple reach success), and gray dots indicate all other trials (e.g., no pellet delivered). Bottom plots show the difference between the mean value on each day and the mean value on day 1. Distributions show the results of a bootstrap resampling procedure with 95% confidence intervals indicated by the solid lines at the left of each distribution. ***C***, Same as ***B*** for the mean distance from the average reach trajectory for each day (top panel shows all hand location data for a single rat that went into Fig. 2*C*, left panel; bottom panel shows all digit 2 location data that went into Fig. 4*A*, left panel). ***D***, Same as ***B***, ***C*** for the reach endpoint analyses. Left column shows reach endpoints in *x*, *y*, and *z* for the hand location (all data for a single rat that went into Fig. 3*B*, left panels). Right column shows reach endpoints for digit 2 with respect to the hand location (all data for a single rat that went into Fig. 3*B*, right panels, except here the hand location was subtracted out). Data and code to generate this figure are contained in Extended Data 1, 2, 5. Download Figure 6-8, TIF file.

10.1523/ENEURO.0153-21.2021.f6-9 Extended Data Figure 6-9 Kinematics summary sheet for a nonlearner rat. ***A***, Success rate across days. Pink lines indicate first reach success, black lines indicate success on any reach attempt for a single trial. ***B***, Shared control plots illustrating measures of hand shaping at reach end (all data for a single rat that went into Fig. 4*C–N*). The top axes are swarm plots showing aperture at reach end for every trial. Pink dots indicate first-reach success trials, black dots indicate first reach failed trials (i.e., pellet remained, pellet knocked off, or multiple reach success), and gray dots indicate all other trials (e.g., no pellet delivered). Bottom plots show the difference between the mean value on each day and the mean value on day 1. Distributions show the results of a bootstrap resampling procedure with 95% confidence intervals indicated by the solid lines at the left of each distribution. ***C***, Same as ***B*** for the mean distance from the average reach trajectory for each day (top panel shows all hand location data for a single rat that went into Fig. 2*C*, left panel; bottom panel shows all digit 2 location data that went into Fig. 4*A*, left panel). ***D***, Same as ***B***, ***C*** for the reach endpoint analyses. Left column shows reach endpoints in *x*, *y*, and *z* for the hand location (all data for a single rat that went into Fig. 3*B*, left panels). Right column shows reach endpoints for digit 2 with respect to the hand location (all data for a single rat that went into Fig. 3*B*, right panels, except here the hand location was subtracted out). Data and code to generate this figure are contained in Extended Data 1, 2, 6. Download Figure 6-9, TIF file.

10.1523/ENEURO.0153-21.2021.f6-10 Extended Data Figure 6-10 Kinematics summary sheet for a nonlearner rat. ***A***, Success rate across days. Pink lines indicate first reach success, black lines indicate success on any reach attempt for a single trial. ***B***, Shared control plots illustrating measures of hand shaping at reach end (all data for a single rat that went into Fig. 4*C–N*). The top axes are swarm plots showing aperture at reach end for every trial. Pink dots indicate first-reach success trials, black dots indicate first reach failed trials (i.e., pellet remained, pellet knocked off, or multiple reach success), and gray dots indicate all other trials (e.g., no pellet delivered). Bottom plots show the difference between the mean value on each day and the mean value on day 1. Distributions show the results of a bootstrap resampling procedure with 95% confidence intervals indicated by the solid lines at the left of each distribution. ***C***, Same as ***B*** for the mean distance from the average reach trajectory for each day (top panel shows all hand location data for a single rat that went into Fig. 2*C*, left panel; bottom panel shows all digit 2 location data that went into Fig. 4*A*, left panel). ***D***, Same as ***B***, ***C*** for the reach endpoint analyses. Left column shows reach endpoints in *x*, *y*, and *z* for the hand location (all data for a single rat that went into Fig. 3*B*, left panels). Right column shows reach endpoints for digit 2 with respect to the hand location (all data for a single rat that went into Fig. 3*B*, right panels, except here the hand location was subtracted out). Data and code to generate this figure are contained in Extended Data 1, 2, 7. Download Figure 6-10, TIF file.

10.1523/ENEURO.0153-21.2021.f6-11 Extended Data Figure 6-11 Kinematics summary sheet for a learner rat. ***A***, Success rate across days. Green lines indicate first reach success, black lines indicate success on any reach attempt for a single trial. ***B***, Shared control plots illustrating measures of hand shaping at reach end (all data for a single rat that went into Fig. 4*C–N*). The top axes are swarm plots showing aperture at reach end for every trial. Green dots indicate first-reach success trials, black dots indicate first reach failed trials (i.e., pellet remained, pellet knocked off, or multiple reach success), and gray dots indicate all other trials (e.g., no pellet delivered). Bottom plots show the difference between the mean value on each day and the mean value on day 1. Distributions show the results of a bootstrap resampling procedure with 95% confidence intervals indicated by the solid lines at the left of each distribution. ***C***, Same as ***B*** for the mean distance from the average reach trajectory for each day (top panel shows all hand location data for a single rat that went into Fig. 2*C*, left panel; bottom panel shows all digit 2 location data that went into Fig. 4*A*, left panel). ***D***, Same as ***B***, ***C*** for the reach endpoint analyses. Left column shows reach endpoints in *x*, *y*, and *z* for the hand location (all data for a single rat that went into Fig. 3*B*, left panels). Right column shows reach endpoints for digit 2 with respect to the hand location (all data for a single rat that went into Fig. 3*B*, right panels, except here the hand location was subtracted out). Data and code to generate this figure are contained in Extended Data 1, 2, 8. Download Figure 6-11, TIF file.

10.1523/ENEURO.0153-21.2021.f6-12 Extended Data Figure 6-12 Kinematics summary sheet for a nonlearner rat. ***A***, Success rate across days. Pink lines indicate first reach success, black lines indicate success on any reach attempt for a single trial. ***A***, Shared control plots illustrating measures of hand shaping at reach end (all data for a single rat that went into Fig. 4*C–N*). The top axes are swarm plots showing aperture at reach end for every trial. Pink dots indicate first-reach success trials, black dots indicate first reach failed trials (i.e., pellet remained, pellet knocked off, or multiple reach success), and gray dots indicate all other trials (e.g., no pellet delivered). Bottom plots show the difference between the mean value on each day and the mean value on day 1. Distributions show the results of a bootstrap resampling procedure with 95% confidence intervals indicated by the solid lines at the left of each distribution. ***C***, Same as ***B*** for the mean distance from the average reach trajectory for each day (top panel shows all hand location data for a single rat that went into Fig. 2*C*, left panel; bottom panel shows all digit 2 location data that went into Fig. 4*A*, left panel). ***D***, Same as ***B***, ***C*** for the reach endpoint analyses. Left column shows reach endpoints in *x*, *y*, and *z* for the hand location (all data for a single rat that went into Fig. 3*B*, left panels). Right column shows reach endpoints for digit 2 with respect to the hand location (all data for a single rat that went into Fig. 3*B*, right panels, except here the hand location was subtracted out). Data and code to generate this figure are contained in Extended Data 1, 2, 9. Download Figure 6-12, TIF file.

10.1523/ENEURO.0153-21.2021.f6-13 Extended Data Figure 6-13 Kinematics summary sheet for a learner rat. ***A***, Success rate across days. Green lines indicate first reach success, black lines indicate success on any reach attempt for a single trial. ***B***, Shared control plots illustrating measures of hand shaping at reach end (all data for a single rat that went into Fig. 4*C–N*). The top axes are swarm plots showing aperture at reach end for every trial. Green dots indicate first-reach success trials, black dots indicate first reach failed trials (i.e., pellet remained, pellet knocked off, or multiple reach success), and gray dots indicate all other trials (e.g., no pellet delivered). Bottom plots show the difference between the mean value on each day and the mean value on day 1. Distributions show the results of a bootstrap resampling procedure with 95% confidence intervals indicated by the solid lines at the left of each distribution. ***C***, Same as ***B*** for the mean distance from the average reach trajectory for each day (top panel shows all hand location data for a single rat that went into Fig. 2*C*, left panel; bottom panel shows all digit 2 location data that went into Fig. 4*A*, left panel). ***D***, Same as ***B***, ***C*** for the reach endpoint analyses. Left column shows reach endpoints in *x*, *y*, and *z* for the hand location (all data for a single rat that went into Fig. 3*B*, left panels). Right column shows reach endpoints for digit 2 with respect to the hand location (all data for a single rat that went into Fig. 3*B*, right panels, except here the hand location was subtracted out). Data and code to generate this figure are contained in Extended Data 1, 2, 10. Download Figure 6-13, TIF file.

10.1523/ENEURO.0153-21.2021.f6-14 Extended Data Figure 6-14 Kinematics summary sheet for a nonlearner rat. ***A***, Success rate across days. Pink lines indicate first reach success, black lines indicate success on any reach attempt for a single trial. ***B***, Shared control plots illustrating measures of hand shaping at reach end (all data for a single rat that went into Fig. 4*C–N*). The top axes are swarm plots showing aperture at reach end for every trial. Pink dots indicate first-reach success trials, black dots indicate first reach failed trials (i.e., pellet remained, pellet knocked off, or multiple reach success), and gray dots indicate all other trials (e.g., no pellet delivered). Bottom plots show the difference between the mean value on each day and the mean value on day 1. Distributions show the results of a bootstrap resampling procedure with 95% confidence intervals indicated by the solid lines at the left of each distribution. ***C***, Same as ***B*** for the mean distance from the average reach trajectory for each day (top panel shows all hand location data for a single rat that went into Fig. 2*C*, left panel; bottom panel shows all digit 2 location data that went into Fig. 4*A*, left panel). ***D***, Same as ***B***, ***C*** for the reach endpoint analyses. Left column shows reach endpoints in *x*, *y*, and *z* for the hand location (all data for a single rat that went into Fig. 3*B*, left panels). Right column shows reach endpoints for digit 2 with respect to the hand location (all data for a single rat that went into Fig. 3*B*, right panels, except here the hand location was subtracted out). Data and code to generate this figure are contained in Extended Data 1, 2, 11. Download Figure 6-14, TIF file.

10.1523/ENEURO.0153-21.2021.f6-15 Extended Data Figure 6-15 Kinematics summary sheet for a nonlearner rat. ***A***, Success rate across days. Pink lines indicate first reach success, black lines indicate success on any reach attempt for a single trial. ***B***, Shared control plots illustrating measures of hand shaping at reach end (all data for a single rat that went into Fig. 4*C–N*). The top axes are swarm plots showing aperture at reach end for every trial. Pink dots indicate first-reach success trials, black dots indicate first reach failed trials (i.e., pellet remained, pellet knocked off, or multiple reach success), and gray dots indicate all other trials (e.g., no pellet delivered). Bottom plots show the difference between the mean value on each day and the mean value on day 1. Distributions show the results of a bootstrap resampling procedure with 95% confidence intervals indicated by the solid lines at the left of each distribution. ***C***, Same as ***B*** for the mean distance from the average reach trajectory for each day (top panel shows all hand location data for a single rat that went into Fig. 2*C*, left panel; bottom panel shows all digit 2 location data that went into Fig. 4*A*, left panel). ***D***, Same as ***B***, ***C*** for the reach endpoint analyses. Left column shows reach endpoints in *x*, *y*, and *z* for the hand location (all data for a single rat that went into Fig. 3*B*, left panels). Right column shows reach endpoints for digit 2 with respect to the hand location (all data for a single rat that went into Fig. 3*B*, right panels, except here the hand location was subtracted out). Data and code to generate this figure are contained in Extended Data 1, 2, 12. Download Figure 6-15, TIF file.

10.1523/ENEURO.0153-21.2021.f6-16 Extended Data Figure 6-16 Kinematics summary sheet for a nonlearner rat. ***A***, Success rate across days. Pink lines indicate first reach success, black lines indicate success on any reach attempt for a single trial. ***B***, Shared control plots illustrating measures of hand shaping at reach end (all data for a single rat that went into Fig. 4*C–N*). The top axes are swarm plots showing aperture at reach end for every trial. Pink dots indicate first-reach success trials, black dots indicate first reach failed trials (i.e., pellet remained, pellet knocked off, or multiple reach success), and gray dots indicate all other trials (e.g., no pellet delivered). Bottom plots show the difference between the mean value on each day and the mean value on day 1. Distributions show the results of a bootstrap resampling procedure with 95% confidence intervals indicated by the solid lines at the left of each distribution. ***C***, Same as ***B*** for the mean distance from the average reach trajectory for each day (top panel shows all hand location data for a single rat that went into Fig. 2*C*, left panel; bottom panel shows all digit 2 location data that went into Fig. 4*A*, left panel). ***D***, Same as ***B***, ***C*** for the reach endpoint analyses. Left column shows reach endpoints in *x*, *y*, and *z* for the hand location (all data for a single rat that went into Fig. 3*B*, left panels). Right column shows reach endpoints for digit 2 with respect to the hand location (all data for a single rat that went into Fig. 3*B*, right panels, except here the hand location was subtracted out). Data and code to generate this figure are contained in Extended Data 1, 2, 13. Download Figure 6-16, TIF file.

Shared control Cumming plots were generated to show individual trial-level data for each rat, separated by successful and failed trials (Extended Data Figs. 6-3, 6-4, 6-5, 6-6, 6-7, 6-8, 6-9, 6-10, 6-11, 6-12, 6-13, 6-14, 6-15,6-16, data for these plots are contained in Extended Data 3, 4, 5, 6, 7, 8, 9, 10, 11, 12, 13). These plots were generated using the Data Analysis with Bootstrap-coupled ESTimation (DABEST) package for Python, version 0.3.1 (Ho et al., 2019). Each plot used 5000 bootstrapped resamples to estimate the difference in the mean between each day and day 1.

10.1523/ENEURO.0153-21.2021.ed3 Extended Data 3 *eNeuro_session_summaries_01*.*zip* contains .mat files needed to create Extended Data Figures 6-3, 6-4*B–D*. Download Extended Data 3, ZIP file.

10.1523/ENEURO.0153-21.2021.ed4 Extended Data 4 *eNeuro_session_summaries_02*.*zip* contains .mat files needed to create Extended Data Figures 6-5, 6-6*B–D*. Download Extended Data 4, ZIP file.

10.1523/ENEURO.0153-21.2021.ed5 Extended Data 5 *eNeuro_session_summaries_03*.*zip* contains .mat files needed to create Extended Data Figures 6-7, 6-8*B–D*. Download Extended Data 5, ZIP file.

10.1523/ENEURO.0153-21.2021.ed6 Extended Data 6 *eNeuro_session_summaries_04*.*zip* contains .mat files needed to create Extended Data Figure 6-9*B–D*. Download Extended Data 6, ZIP file.

10.1523/ENEURO.0153-21.2021.ed7 Extended Data 7 *eNeuro_session_summaries_05*.*zip* contains .mat files needed to create Extended Data Figure 6-10*B–D*. Download Extended Data 7, ZIP file.

10.1523/ENEURO.0153-21.2021.ed8 Extended Data 8 *eNeuro_session_summaries_06*.*zip* contains .mat files needed to create Extended Data Figure 6-11*B–D*. Download Extended Data 8, ZIP file.

10.1523/ENEURO.0153-21.2021.ed9 Extended Data 9 *eNeuro_session_summaries_07*.*zip* contains .mat files needed to create Extended Data Figure 6-12*B–D*. Download Extended Data 9, ZIP file.

10.1523/ENEURO.0153-21.2021.ed10 Extended Data 10 *eNeuro_session_summaries_08*.*zip* contains .mat files needed to create Extended Data Figure 6-13*B–D*. Download Extended Data 10, ZIP file.

10.1523/ENEURO.0153-21.2021.ed11 Extended Data 11 *eNeuro_session_summaries_09*.*zip* contains .mat files needed to create Extended Data Figure 6-14*B–D*. Download Extended Data 11, ZIP file.

10.1523/ENEURO.0153-21.2021.ed12 Extended Data 12 *eNeuro_session_summaries_10*.*zip* contains .mat files needed to create Extended Data Figure 6-15*B–D*. Download Extended Data 12, ZIP file.

10.1523/ENEURO.0153-21.2021.ed13 Extended Data 13 *eNeuro_session_summaries_11*.*zip* contains .mat files needed to create Extended Data Figure 6-16*B–D*. Download Extended Data 13, ZIP file.

### Code accessibility

The code described in this paper is freely available online at https://github.com/LeventhalLab/Bova_etal_eNeuro_2021.git. The code is available as Extended Data 1.

## Results

Rats were trained for 10 d of 30 min sessions in an automated skilled reaching task in which high-definition, high-frame rate video is recorded from multiple angles. Trials started with rats breaking a photobeam at the back of the chamber, which caused a pellet to be delivered in front of the reaching slot (Fig. 1*A*). Rats could make multiple reaches until the pellet delivery arm descended 2 s after the video trigger event. Rats showed significant improvement in skilled reaching performance, as first reach success rate gradually increased (Fig. 1*C*). However, there was a high level of variability in learning rates. Some rats reached asymptotic performance quickly, while others did not improve at all (Extended Data Fig. 1-1*B*). Therefore, we divided rats into learners and non-learners based on whether their performance improved significantly from days 1 to 2 to 9 to 10 (see Materials and Methods, Performance outcomes; Table 2). Although success rate was similar on training day 1, performance improved significantly over days for learners, but not non-learners. Learners showed a significant reduction of trials in which the pellet was knocked off of the pellet delivery rod over training days, whereas this outcome did not change significantly over days for non-learners (Extended Data Fig. 1-1*D*). Of the other trial outcomes (e.g., dropped pellet in box, pellet remained on pedestal; see Materials and Methods, Performance outcomes), only multiple success outcomes, where rats did not successfully retrieve the pellet on the first reach attempt but did on a subsequent attempt, decreased significantly for learners. Learners seemed to convert “multiple reach successes” and “pellet knocked off” trials into “first reach successes.”

**Table 2:** χ^2^ test results to determine learners versus non-learners

Rat	Sex	h	*p*	χ^2^ stat	df	Positive change?	Learner/non-learner
A	M	0	0.89	0.02	1	Yes	Non-learner
B	M	1	0.04	4.45	1	Yes	Learner
C	M	0	0.28	1.15	1	Yes	Non-learner
D	M	0	0.26	1.28	1	Yes	Non-learner
E	F	0	0.21	1.57	1	Yes	Non-learner
F	M	1	4.58e-11	43.35	1	Yes	Learner
G	M	0	0.63	0.24	1	Yes	Non-learner
H	F	0	0.22	1.54	1	Yes	Non-learner
I	F	1	0.02	5.52	1	Yes	Learner
J	F	1	4.98e-4	12.13	1	Yes	Learner
K	F	0	0.46	0.54	1	No	Non-learner
L	M	1	0.02	5.38	1	No	Non-learner
M	M	0	0.87	0.03	1	Yes	Non-learner
N	M	0	0.91	0.01	1	No	Non-learner

Importantly, rats in the two groups performed similar numbers of trials over the 10 training days, indicating that the difference between learners and non-learners was not in practice or motivation (Fig. 1*B*; Extended Data Fig. 1-1*A*).

### Refinement of gross forelimb movements

To determine how reach-to-grasp kinematics evolved over the first 10 d of learning, we used DeepLabCut to track individual digits, the hand, and the pellet (Mathis et al., 2018). Skilled reaching requires refinement of both “gross” forelimb movements to accurately guide the hand to the pellet, and “fine” digit movements to grasp the pellet. Forelimb movements became more consistent over 10 d of training for both learners and non-learners (Fig. 2*A–D*). This was true for both “reach” and “grasp” components, which were distinguished by when the digits began to close (Fig. 2*B*; see Materials and Methods, Processing reach kinematics). The variability of the hand trajectory decreased significantly over the first six training sessions and then remained stable in the last four sessions (Fig. 2*C*). This stabilization occurred approximately when first reach success rate peaked for learners (Fig. 1*C*). However, there was considerable variability between rats in how quickly trajectory variability changed (Extended Data Fig. 2-1*A*,*B*). Changes in hand trajectory variability and first reach success rate within individual sessions often occurred in tandem, such that when success rate increased, trajectory variability decreased and vice versa (Fig. 2*D*). Reaches also became faster with more training in both groups. The average reach duration decreased (Fig. 2*E*), while maximum reach velocity increased (Fig. 2*F*).

As hand trajectories stabilized, rats also improved their reach endpoint accuracy. In the first two training days, reaches terminated at positions above and short of the pellet for learners and non-learners (Fig. 3*A*,*B*). However, by the third session, reaches became lower and longer, with reach endpoints in the *Y* and *Z* directions stabilizing for the remaining sessions.

Reach endpoint variability of the hand and digits decreased considerably for both groups (Fig. 3*C*,*D*; Extended Data Fig. 3-2). One potential explanation for the decrease in digit endpoint variability is that they are attached to the hand. To determine whether digit endpoint variability decreased more than expected from the decrease in hand endpoint variability, we calculated digit location with respect to the hand (i.e., 3D hand location was subtracted from the digit position; Fig. 3*D*, bottom row). As expected, digit endpoint variability was much smaller when corrected for hand position. While only changes in digit 2 endpoint variability achieved significance, there was a consistent decrease in digit endpoint variability out to day 10 for the learner group. Therefore, variability in digit endpoint location is largely accounted for by variability in hand trajectory, but additional refinement of digit movement may account for some of the differences in reach success across rats.

Learners had consistently more stable reach trajectories compared with non-learners, but these differences rarely achieved statistical significance. This may be explained by examining data at the level of individual rats (Extended Data Figs. 2-1, 3-1, 6-3, 6-4, 6-5, 6-6, 6-7, 6-8, 6-9, 6-10, 6-11, 6-12, 6-13, 6-14, 6-15, 6-16). Learners tended to converge on similar kinematics, regardless of the metric. While the kinematics for many non-learners showed a high degree of variability, other non-learners had kinematic patterns very similar to learners. This suggests that the term non-learner may be inappropriate for some of these rats, which adapted their reach kinematics without the payoff of increased reach success.

To evaluate whether decreases in endpoint variability could be artifacts of inconsistencies in DeepLabCut labeling, we calculated the percentage of frames from reach start to reach grasp that were mislabeled for each part on days 1 and 10 (Fig. 3*E*; see Materials and Methods, Processing reach kinematics). More mislabeled frames early in training would suggest that the apparent decrease in variability across sessions is really because of improved DeepLabCut reliability. For the most part, the number of mislabeled frames was consistent between days 1 and 10. There were significantly more mislabeled frames on day 10 compared with day 1 for digit 4, but if anything, this should artificially increase endpoint variability on day 10.

### Refinement of fine digit movements

It is suggested that fine digit control continues to be refined once gross forelimb trajectories have stabilized (Lemke et al., 2019). We therefore examined changes in forelimb-digit coordination as assessed by digit location, digit aperture, hand orientation, and digit flexion (for definitions of these terms, see Fig. 4*C*,*G*,*K*). Trajectory variability of the second digit plateaued during the same session as the paw trajectory (compare Fig. 4*A*,*B*, Extended Data Fig. 4-1*A*,*B* and Fig. 2*C*), consistent with the finding that most variability in digit position is accounted for by variability in hand position (Fig. 3*D*). Grasp aperture (*a* in Fig. 4*C*) at reach end appeared to gradually increase on average (Fig. 4*D*,*E*). However, some rats increased grasp aperture over training days while other rats decreased grasp aperture (Extended Data Figs. 4-1*C*, 6-3, 6-4, 6-5, 6-6, 6-7, 6-8, 6-9, 6-10, 6-11, 6-12, 6-13, 6-14, 6-15, 6-16). Despite this, the variance of grasp aperture at reach end decreased consistently across rats (Fig. 4*F*; Extended Data Fig. 4-1*D*). However, these changes were not significant for either group.

The orientation of the paw at reach end (θ in Fig. 4*G*) also changed over the 10 training sessions, such that the paw became less pronated (Fig. 4*H*,*I*). Accordingly, the variability of the paw angle at reach end [measured by mean resultant length (MRL)] decreased over learning (Fig. 4*J*). Finally, the degree of digit flexion decreased over learning (δ in Fig. 4*K*), so that the digits were more extended at the end of the reach in later sessions (Fig. 4*L*,*M*). Again, the variability (MRL) in digit flexion across trials also decreased with training. Thus, learning to shape the paw and digits in preparation for grasping appears to require more practice than learning to efficiently guide the paw toward the pellet.

None of these measurements differed significantly between learners and non-learners, though in general variability in digit trajectory/hand shape was lower for learners (Fig. 4*A*, days 7–10; Fig. 4*F*,*N*). As in the gross trajectory analysis, many of the non-learners adapted their fine digit movements in ways similar to learners (Extended Data Figs. 4–1, 6-3, 6-5, 6-6, 6-7, 6-8, 6-9, 6-10, 6-11, 6-12, 6-13, 6-14, 6-15, 6-16), while others did not. Furthermore, different rats seemed to adapt different kinematic measures at different rates. For example, rat I (Extended Data Fig. 6-11) adjusted hand orientation after digit aperture stabilized, while rat N (Extended Data Fig. 6-16) adjusted aperture after orientation had stabilized. This again emphasizes the variability in motor control/learning across rats, and that a lack of improvement in reach success does not necessarily mean that rats are not adapting their reaching strategies.

The reach-to-grasp movement not only requires rats to spread and extend their digits, orient their paw, and grasp, but that these digit movements be properly timed with respect to paw advancement. Therefore, we evaluated how the coordinated execution of paw and digit shaping changed by plotting digit aperture, paw orientation, and digit flexion as a function of paw advancement toward the pellet. To quantify changes in forelimb-digit coordination, we tested differences in these quantities when the tip of digit 2 was 3 mm past the pellet. Overall, there was a suggestion that rats in both groups spread their digits (Fig. 5*A*,*B*), pronated their paws (Fig. 5*C*,*D*), and extended their digits (Fig. 5*E*,*F*) earlier along the reach trajectory with training. However, only the changes in hand orientation achieved statistical significance in the linear mixed model.

### Kinematic differences between successful and failed reaches

Skilled reaching success increased for some but not all rats. Even for learners, however, rats still missed or knocked the pellet off the pedestal frequently in the later sessions. This could be because of persistent variability in reach/grasp kinematics or because even optimal kinematics have a significant failure rate (i.e., the task is inherently difficult). Therefore, we next asked whether reach-to-grasp kinematics differed between successful and unsuccessful trials, and whether those differences changed with learning. Trajectory variability for both successful and unsuccessful reaches decreased over 10 d of training. Especially in early sessions, trajectory variability was larger for failed than successful trials. For learners, trajectory variability for successful and failed reaches converged with training so that there was little (if any) difference by session 10 (Fig. 6; Extended Data Figs. 6-1, 6-3, 6-4, 6-5, 6-6, 6-7, 6-8, 6-9, 6-10, 6-11, 6-12, 6-13, 6-14, 6-15, 6-16). For non-learners, however, differences between successful and failed reaches persisted despite improved consistency in reach kinematics overall. Thus, a key difference between learners and non-learners was persistence of failed reach variability with training.

## Discussion

Differences in skilled reaching success across rats are partially, but not completely, explained by changes in forelimb and digit kinematics. Rats that improved their reaching performance as assessed by increased success rate (learners) converged on consistent gross forelimb kinematics, after which they continued to refine fine digit movements. However, individual rats may refine different aspects of reaching in different orders. Most non-learners also adapted their gross reach kinematics at a rate similar to learners, though some maintained a high degree of gross kinematic variability throughout training. This further emphasizes the importance of measuring forelimb kinematics, and not just success rates, when assessing the effects of interventions on skilled reaching. We found two subtle, but important, differences between learners and non-learners. First, learners showed continued decreases in digit endpoint variability (even accounting for variability in hand position). Second, there was more persistent variability in failed reach trajectories for non-learners.

In general, learning curves were shallower and success rates lower for our rats compared with similar tasks (Alaverdashvili and Whishaw, 2008; Wong et al., 2015; Lemke et al., 2019). This could be because of relatively low trial counts in early sessions, possibly related to the transition from manual pretraining to the automated task. Even after well over 100 trials, however, success rates did not increase for 10/14 rats despite significant changes in limb kinematics. This task is inherently difficult, as slight perturbations to the pellet cause it to fall off the narrow pedestal. This contrasts with tasks in which the pellet is on a tray or shelf, allowing the rat to slide the pellet before securely grasping it. We may have found a higher proportion of learners if the task were more forgiving.

There are several potential explanations for why many non-learners did not improve their success rates despite changes in reach kinematics similar to learners. One possibility is that learners perform more trials per session, which accelerates learning rates (Wong et al., 2015). However, learners and non-learners performed similar numbers of trials (Fig. 1*B*; Extended Data Fig. 1-1*A*), and kinematics changed on roughly the same timescale for both groups. It therefore seems unlikely that differences in motivation or practice account for differences in success rates. Biomechanical differences between rats could also limit success. For example, rats with small hands may not be capable of achieving reach apertures sufficient to improve success rates. Differences in sensory acuity, both in directing the initial reach (rats rely heavily on olfaction to direct reaches; Whishaw and Tomie, 1989), or somatosensation to refine their grasp, could affect success rates and kinematic adaptation. Another possibility is that these rats would have increased their success rates with additional training, as was observed in an “extended training” group trained for several weeks in a previous study (Lemke et al., 2019). Finally, and perhaps most interesting, is the possibility that there are innate differences in neural circuits/plasticity between learners, non-learners that refine their reach kinematics, and non-learners in which trajectory variability persists. The possible neural substrates for such variability are broad. One candidate is differences in dopamine signaling/receptor profiles, as dopamine plays an important role in motor adaptation (Panigrahi et al., 2015; Bova et al., 2020). Other possibilities include differences in cortical plasticity (Li et al., 2017; Hyland et al., 2019) and corticostriatal coherence (Lemke et al., 2019).

Regardless of mechanism, our data suggest that non-learners did not make final, subtle adjustments in digit kinematics necessary to firmly grasp and retrieve sugar pellets. This is consistent with previous work suggesting that gross forelimb movements for guiding the hand to the pellet and fine digit movements for grasping the pellet are learned as separate “reach” and “grasp” modules (Li et al., 2017; Lemke et al., 2019). However, in these studies, refinement of fine motor control was inferred from increases in success rate, rather than direct measures of digit kinematics. We show that fine digit movements to prepare for grasping (i.e., precisely adjusting final digit location, spreading and extending digits, pronating the hand) are refined after gross movement kinematics (i.e., forelimb trajectory variability, reach velocity) have stabilized, at least in learners. Therefore, learners first learn to accurately and efficiently guide their hands toward the pellet, then to appropriately shape their digits and orient their hands at the correct moments to grasp the pellet. This timing may be reflected in corticostriatal coherence at low frequencies (3–6 Hz), which are phase-locked to grasp onset in an almost identical task (Lemke et al., 2019).

That gross and fine motor components of reaching are learned sequentially, rather than concurrently, suggests that distinct neural mechanisms regulate and implement these aspects of motor control. This concept of modular circuits for reaching and grasping is supported by several physiologic experiments. Reach and grasp movements are elicited by microstimulation in distinct areas of cortex (caudal and rostral forelimb areas, respectively; Brown and Teskey, 2014). Furthermore, perturbations at different downstream loci of cerebral motor circuits selectively disrupt either reaching or grasping movements. Chemogenetic silencing of spinal interneurons that receive inputs from either motor or sensory cortex interfered with reaching or grasping, respectively (Ueno et al., 2018). This suggests that distinct circuits for reaching and grasping are maintained at all levels of motor circuitry.

However, reach-to-grasp movements require that the digits shape in preparation for grasping as the paw advances toward the pellet. Therefore, improving skilled reaching performance requires not only refinement of independent gross and fine motor components, but also their temporal and spatial integration. We found that coordinated execution of digit movements (e.g., spreading and extending the digits) with paw advancement continued to change across all 10 d of training. This suggests that fine digit movements are not regulated by circuits entirely distinct from those used to refine gross movements, or at least that communication between “reach” and “grasp” modules also evolves with practice.

Temporospatial integration of fine and gross motor control could be implemented in cortico-basal ganglia pathways. As rats learned skilled reaching, coordinated activity between dorsolateral striatum and motor cortex emerged, and inactivation of dorsolateral striatum disrupted movement-related cortical activity (Lemke et al., 2019). This suggested that coordinated corticostriatal activity is essential for refining reach-to-grasp movements. However, this coordinated activity only appeared to correlate with changes in gross movement kinematics and not with success rate (i.e., coordinated activity did not differ between successful and failed reaches), and it was concluded that striatum regulates gross, not fine, motor control. However, the present findings show that fine digit movements continue to change even after success rate (and presumably corticostriatal coherence) had stabilized in individual rats (Figs. 3, 4; Extended Data Figs. 6-4, 6-8, 6-11, 6-13). Thus, coordinated corticostriatal activity could be correlated with changes in fine digit kinematics not evident from success rate. Indeed, previous work found that optogenetic manipulations of substantia nigra pars compacta dopamine neurons during skilled reaching disrupted the coordinated execution of digit movements with gross forelimb movements (Bova et al., 2020). Therefore, basal ganglia circuitry does regulate some aspects of fine digit control, or at least its timing with respect to gross forelimb movements. This may be accomplished through basal ganglia regulation of motor cortex activity.

Fine digit movements continued to be refined after success rate had stabilized in learners, suggesting that aspects of motor control that do not affect task outcome are optimized over a longer period of time. One possibility is that movements were refined further to maximize efficiency in terms of either time or energy consumption. In this view, the execution of reach-to-grasp movements requires balancing the desire to obtain as many rewards as possible with minimizing effort and maximizing accuracy. Humans and primates increase movement vigor when those movements are paired with reward, suggesting that reward mitigates the cost of effort (Takikawa et al., 2002; Shadmehr et al., 2010; Manohar et al., 2015; Summerside et al., 2018). However, movements often have a speed-accuracy trade-off, as movements that are faster or larger become less accurate (Shmuelof et al., 2012). Reach duration decreased and reach velocity increased over the first few training sessions, but then plateaued, suggesting that movement speed reached a threshold at which maximizing reward was balanced with maintaining accuracy. However, it is possible that once the speed of reaching movements was optimized, rats then attempted to reduce energy cost by refining digit movements. One way of minimizing effort is to efficiently distribute work across multiple muscles or joints (Diedrichsen et al., 2010). Improving digit shaping before grasping may reduce energetic costs by minimizing the number of corrective movements.

Despite overall improvements in performance, there was still significant variability in reach-to-grasp movements after success rate stabilized, even in learners. Because the extrinsic features of the task do not change (e.g., the pellet is always in the same position, shape and size of pellet are consistent, etc.), it is likely that performance variability in late training sessions arises from changes in internal states. There are several potential sources of this internal variability. Manohar et al. (2015) found that increased reward enhanced saccade accuracy, and that saccade accuracy in Parkinson Disease patients was insensitive to reward. This suggests that increased motivation reduces motor noise to improve movement accuracy, possibly through dopaminergic mechanisms (Manohar et al., 2015). Similarly, male song in zebra finches is more stereotyped when performing for a female during courtship than when practicing alone (Kao et al., 2005). Furthermore, female-directed song is associated with enhanced extracellular dopamine levels in Area X (Ihle et al., 2015). Thus, variability in rodent skilled reaching may arise from varying motivation during a session. Another potential source of variability is the degree to which stimuli unrelated to the current task are attended (Roberts et al., 2021). While ignoring irrelevant information may improve task performance, ignoring potentially salient information carries a cost. For example, sensory information could warn of impending danger or signal existence of a better opportunity for reward. Therefore, even in a controlled laboratory environment, the brain may not be conditioned to persistently filter competing distractors for extended periods of time. Finally, movement variability may persist so that motor circuits can adapt if task rules change. Variability during early motor skill acquisition contributes to learning by allowing exploration of optimal motor patterns (Dhawale et al., 2017). Although extrinsic features of our skilled reaching task do not change, some level of variability could be maintained to allow fast adaptation if they did.

Whether reaching kinematics remain variable or become more stereotyped could depend on tonic dopamine levels in striatum, which is thought to control the trade-off between exploration (i.e., try different strategies) and exploitation (i.e., continue to use the same strategy; Humphries et al., 2012). Performance improvement during skilled reaching learning was prevented by ventral tegmental area dopamine lesions (Hosp et al., 2011). However, it is not clear whether learning was impaired because low dopamine levels prevented exploration for optimal reaching strategies, prevented rats from learning to exploit the optimal reaching strategy, or for other reasons. Experiments that directly assess changes in reach kinematics with dopamine manipulations in different brain regions during learning could address these questions. Similarly, it will be important to determine how dopamine signaling changes during skill acquisition across relevant brain regions (e.g., motor cortex and striatum), and how such changes correlate with task performance. Finally, it is not known whether differences in motor learning are correlated with differences in other domains (e.g., instrumental or Pavlovian conditioning).

Future studies will need to address potential explanations for continued refinement of fine digit kinematics and variability after the stabilization of performance outcomes. However, our results provide a foundational understanding of how skilled reach-to-grasp kinematics in rats evolve during learning, and illustrate the importance of intersubject variability. These findings are essential for interpreting results of studies applying neural circuit manipulations during learning or performance of skilled reaching, and are a crucial step toward understanding how neurologic disorders disrupt dexterous motor skill learning and performance.

## Synthesis

Reviewing Editor: Mark Laubach, American University Decisions are customarily a result of the Reviewing Editor and the peer reviewers coming together and discussing their recommendations until a consensus is reached. When revisions are invited, a fact-based synthesis statement explaining their decision and outlining what is needed to prepare a revision will be listed below. The following reviewer(s) agreed to reveal their identity: Karunesh Ganguly, Gergely Silasi.

Both reviewers found the study interesting and support publication provided that some essential revisions are done to the manuscript. Please respond to each point raised by both reviewers in your rebuttal and please note specific changes in your manuscript (page and line numbers) for each revision. Thank you.

Also, I would like to say that I very much appreciated your very clear figures and reporting of all statistical measures. You should consider using estimation statistics (confidence intervals and permutation tests) for this study, given the sample sizes. There is a very easy to use Python package for these measures, called DABEST. https://www.estimationstats.com/ https://acclab.github.io/DABEST-python-docs/index.html

Reviewer #1

This manuscript is a nice contribution to the literature. The rodent reach-to-grasp task is widely used in innumerable studies of normal motor learning as well as in pre-clinical studies. The vast majority of studies rely on gross measures of learning; this study uses new advances in computer vision to track in detail the kinematics of the arm and hand/grasping over the course of learning. Overall, these results will be of interest to many in the field.

My main concern is that the rates of learning are not as great at the population level (e.g. Fig 1). There is only a modest gain in performance over the many sessions. Moreover, while the use of mixed effects statistics is great, it is very sensitive and it seems that the effects sizes are small, at least at the population level. Perhaps one way forward is to spilt animals into ‘learners’ vs ‘non-learners.’ That some animals do not learn is not surprising and reported in the literature.

This might also help emphasize the effects of learning and presumed skill formation, vs poor learning and maintained states of exploration.

Major.

1. Effects of learning are not clear. For example, Fig 1c shows very modest changes in performance. Perhaps have a criteria per animal for learning and separate into two groups. Also, would help to show trends per animals (hard to tell in Fig 1c). It might be interesting to look at regulation of variability with learning vs no learning.

2. How many trials per session were there? Are sessions = days? It would be best to mark days (and number of trials).

3. For finger variability, I don’t believe that the paw position was subtracted. For example, if the finger never moved and the paw position was quite variable, it might look as if the finger was variable. But, subtraction of the paw position would show a stable finger. This is most relevant for Fig 3 (e.g. 3C), where the two seem quite similar at times.

4. Fig 2D: How was the entire kinematic trace converted to a distance? Average of all points?

5. Fig 6. How was each kinematic trace converted to a single distance?

6. Fig 6. This would be good to look at for learners vs non-leaners. It is more likely that learners become habitual/automatic and less sensitive to outcomes.

Minor.

1. Not clear how many animals are actually used? e.g. fig 1, put n=xx

2. Fig 2A. Hard to tell the finger vs paw traces; bolder lines and labels

3. It is not clear what the determinant of the variance is? Please provide additional details.

4. Fig 4C/G/K. Please show a cartoon and then actual image. It is hard to tell what metric is being used from the actual image.

5. Discussion: Please note that the Lemke et al paper found an effect of grasp timing and striatal activity for reach-to-grasp coordination. More specifically, with learning, there was an increase in inter-trial coherence of grasp onset timing to striatal theta (Fig 2d). This is likely important for reach-to-grasp coordination and timing of grasping.

Reviewer #2

The paper applies newly developed methods to carrying out a detailed analysis of the fine hand movements acquired during skilled reaching in rats. The methods used in the current experiment have been previously applied to automated testing of rats on skilled reaching, and the current study provides additional details about this approach. The ability to track 3D trajectories for not only the hand, but individual digits in an automated reaching task is an extremely valuable contribution to the field, and the current study provides novel measures of the fine kinematic details during skill acquisition. The study is in general clearly written, and supported by informative figures. The main criticism of the study is that perhaps because of the low number of subjects included, the variability in performance makes one question whether some of the interpretations of the data are accurate. Specifically, the notion that digit kinematics continue to be refined after the gross motor skill of reaching is stabilized is not completely apparent from the data. Specific comments include:

- Methods on P5 indicate video recording started when paw was in ROI? Where is this ROI relative to the paw passing through the front slot? Is some of the movement not being captured as it happens before recording trigger?

- From the description of within session kinematics, it appears that a rat that performed 35 trials would contribute the same amount of data as a rat that performed 40 trials. This seems inherently incorrect, even when data are averaged over 10 consecutive trials as the authors indicate. Do the results hold if the authors take the more conservative approach and analyze only 35 trials from all subjects?

- Authors refer to the paw of the rat, however Whishaw has clearly outlined reasons why rats and mice have hands, not paws. The authors should probably call it a hand consistently throughout the manuscript and figures.

- Fig 2A: Which trials are plotted? All successful reaches from all rats?

- Fig 2D: difficult to discern that x axis labels trial numbers 15 and 30? First read as 1530...

o Related to previous point, did all rats in this graph perform at least 30 trials, or was data extrapolated as described in methods?

- Images in Fig 3A could be labeled with “Side (mirror) view” and “front view” for clarity

- Data in Figure 3 suggest there is decrease in digit variability after initial few training sessions, and data also show that end-point variability of hand position is lower than digit position. Given that these conclusions are based on data from DLC one may wonder if the accuracy and consistency of the markerless tracking is different between the hand and the digits. This could explain some of the effects. In addition, increased labeling variability may also be introduced if the posture of the hand is different from what the network was trained on ie. the network may have been optimized for accurately detecting the hand in latter training sessions, and therefore performs more variably during initial testing sessions. It may be that the rats have a different (but equally consistent) hand posture early in training. Could the authors compare confidence values and the number of mislabelled frames from early vs. latter training to see if the networks performs inference equally well at these different timepoints? Similar analysis of labelling accuracy should be compared for hand vs. digit labels.

- The main interpretation of the data, that refinement of digit movements continues beyond when success rate and trajectory have stabilized is not fully supported by the data that’s shown. For example, fig 2C shows that distance from average trajectory decreases, while success rate increases but these changes occur essentially at the same time. Perhaps the most direct effect to support the authors claim is in figure 4, where the angle of the digits (panel g-j) changed around the 6th day of training, while the authors claim that the increase in reaching performance had stabilized by that point. However the reaching accuracy does indeed fluctuate between sessions 6-10, and the changes in hand kinematics are partially overlapping with this time-course. Either toning back the claims on the sequential development of gross and fine motor skill or perhaps providing additional evidence to support these statements would be required.

## Author Response

REVIEWER COMMENT:

Synthesis Statement for Author (Required):

Both reviewers found the study interesting and support publication provided that some essential revisions are done to the manuscript. Please respond to each point raised by both reviewers in your rebuttal and please note specific changes in your manuscript (page and line numbers) for each revision. Thank you.

Also, I would like to say that I very much appreciated your very clear figures and reporting of all statistical measures. You should consider using estimation statistics (confidence intervals and permutation tests) for this study, given the sample sizes. There is a very easy to use Python package for these measures, called DABEST. https://www.estimationstats.com/ https://acclab.github.io/DABEST-python-docs/index.html

RESPONSE:

We would like to thank Dr. Laubach and the reviewers for their favorable impression of our manuscript, and suggestions that we found very helpful in refining our thoughts.

We read Dr. Bernard’s editorial on estimation statistics and associated commentary by Calin-Jageman and Cumming (2019) with interest. We agree that this makes a lot of sense. Thank you for pointing out the DABEST package, which provides nice tools for elegant plots. However, we could not find appropriate statistics/plots to account for the structure of our data (repeated measures across multiple sessions - this is acknowledged in the DABEST Github repository - https://github.com/ACCLAB/DABEST-python#about).

That being said, Reviewer #1’s suggestion to split the rats into “learners” and “non-learners” highlighted the individual differences across rats, and we found the shared control plot in DABEST a useful way to highlight these differences in the new supplemental “summary sheets” for each rat (Extended Data 6-3 to 6-16). The “contrast axes” (bottom axes in each shared control plot) make it easier to see when changes are stabilizing across days. We further discuss these plots in our response to Reviewer #2’s last point.

REVIEWER COMMENT:

Reviewer #1

This manuscript is a nice contribution to the literature. The rodent reach-to-grasp task is widely used in innumerable studies of normal motor learning as well as in pre-clinical studies. The vast majority of studies rely on gross measures of learning; this study uses new advances in computer vision to track in detail the kinematics of the arm and hand/grasping over the course of learning. Overall, these results will be of interest to many in the field.

My main concern is that the rates of learning are not as great at the population level (e.g. Fig 1). There is only a modest gain in performance over the many sessions. Moreover, while the use of mixed effects statistics is great, it is very sensitive and it seems that the effects sizes are small, at least at the population level. Perhaps one way forward is to spilt animals into ’learners’ vs ’non-learners.' That some animals do not learn is not surprising and reported in the literature.

This might also help emphasize the effects of learning and presumed skill formation, vs poor learning and maintained states of exploration.

Major.

1. Effects of learning are not clear. For example, Fig 1c shows very modest changes in performance. Perhaps have a criteria per animal for learning and separate into two groups. Also, would help to show trends per animals (hard to tell in Fig 1c). It might be interesting to look at regulation of variability with learning vs no learning.

RESPONSE:

We thank the reviewer for this suggestion - it helped highlight the variability in learning across rats, and also provided insight into how this skill is refined even in the absence of improvements in success rate. It has dramatically changed the way we framed and interpreted our data.

We split animals into “learners” and “non-learners” based on performance (first reach success rate) in the first two and last two training days. The differences in performance between these groups are shown clearly in Figure 1C. All other analyses are shown for both learners and non-learners, but we also include averages for all rats in each figure. What stands out is how similar the learners and non-learners are in terms of kinematic changes with a few key differences.

First, aperture was consistently wider in the learners (though the difference was not significant). Second, learners had fairly consistent kinematics whether or not they were successful; non-learners exhibited significantly different kinematics on failed compared to successful trials (see response to point 6). Finally, “learners” refined their digit movements in late sessions, independently of changes in hand location (see response to point 3).

The Extended Data Figures now show individual animal data separately for learners and non-learners so that trends per animal are clearer. We also included summary sheets for each rat, taking advantage of the DABEST package suggested by Dr. Laubach, to better illustrate per-animal trends and highlight the fact that many “non-learners” refined their reaching strategy in a way very similar to learners.

REVIEWER COMMENT:

2. How many trials per session were there? Are sessions = days? It would be best to mark days (and number of trials).

RESPONSE:

The average number of trials per session is shown in Figure 1B (individual rat data are in Extended Data Figure 1-1). Training sessions lasted 30 minutes, and rats could perform as many trials as they could in those 30 minutes. We changed “sessions” to “days” where appropriate in the figures and text to make it clearer that training sessions occurred on consecutive days. We also clarified in the Methods that one session was performed per day (under Materials and Methods-->Skilled reaching-->Training).

REVIEWER COMMENT:

3. For finger variability, I don't believe that the paw position was subtracted. For example, if the finger never moved and the paw position was quite variable, it might look as if the finger was variable. But, subtraction of the paw position would show a stable finger. This is most relevant for Fig 3 (e.g. 3C), where the two seem quite similar at times.

RESPONSE:

This is a good point. In Figure 3D, we now show endpoint variability of the “raw” digit position data (top row) and the subtracted digit position data (bottom row). Our original interpretation was that digit endpoint variability was larger than hand endpoint variability, suggesting that there was some refinement of digit position independent of hand position. The new analysis confirmed this interpretation, but only for “learners.” We also plotted Figure 3D on a logarithmic scale because generalized variance can take on a wide range of values, which made these differences clearer. We changed our interpretation to indicate that the variability in digit position is largely accounted for by variability in hand position, but that learners continue to refine their fine digit movements (third paragraph in Results-->Refinement of gross forelimb movements).

REVIEWER COMMENT:

4. Fig 2D: How was the entire kinematic trace converted to a distance? Average of all points?

RESPONSE:

Calculating trajectory variability presented several problems. The first was how to define variability, for which we decided to use the mean distance from the mean trajectory. This immediately presented a second problem - how to define the mean trajectory. We considered averaging corresponding points across trajectories - that is, the ith point in the average would be the sum of the ith point from each trajectory divided by the number of trajectories. However, we realized that fast or slow trajectories could distort the mean trajectory. For example, on one reach the nth point might be during the grasp phase, while for another the paw may just be coming through the slot, even though the two trajectories could overlap perfectly in space.

To deal with this problem, each trajectory was interpolated so that it was made up of 100 evenly spaced points (described in the last paragraph of “Processing Reach Kinematics” in the Methods). That way, the interpolated points should correspond to similar points along each trajectory. Each interpolated point was then averaged to create an average 100-point trajectory. To quantify trajectory variability, the mean distance from each trial's interpolated trajectory to the average trajectory was calculated on a point-by-point basis (i.e., |X1-▁X1|, |X2-▁X2|,...|X100-▁X100|. These distances from the mean trajectory were averaged to generate session-wide measure of trajectory variability. These values are plotted in Figures 2C, D; 4A, B; and 6A. We rewrote the relevant Methods section (third paragraph in Materials and Methods-->Processing Reach Kinematics), including an equation which hopefully clarifies how the calculations were performed.

REVIEWER COMMENT:

- Related to previous point, did all rats in this graph perform at least 30 trials, or was data extrapolated as described in methods?

RESPONSE:

As can be seen in Extended Data Figure 1-1A, not all rats performed at least 30 trials on all training days. Therefore, data was extrapolated as described in the methods. However, after the first few days, almost all rats performed more than 30 trials.

REVIEWER COMMENT:

5. Fig 6. How was each kinematic trace converted to a single distance?

RESPONSE:

See our response to point 4 - the same calculation was used for Figures 2, 4, and 6.

REVIEWER COMMENT:

6. Fig 6. This would be good to look at for learners vs non-leaners. It is more likely that learners become habitual/automatic and less sensitive to outcomes.

RESPONSE:

We now include the analysis for 'learners' vs 'non-learners' in Figure 6. This is one of the more interesting findings to come out of the re-analysis suggested by Reviewer 1. While learners have fairly consistent kinematics regardless of reach outcome, non-learners seem to have 2 types of reaches with fairly distinct kinematics. This is also evident in the individual rat summary sheets (Extended data 6-3 to 6-16).

REVIEWER COMMENT:

Minor.

1. Not clear how many animals are actually used? e.g. fig 1, put n=xx

RESPONSE:

We added numbers of animals per group (n=xx) in Figure 1B.

REVIEWER COMMENT:

2. Fig 2A. Hard to tell the finger vs paw traces; bolder lines and labels

RESPONSE:

We made the lines and labels thicker and bolder. We hope the traces are easier to differentiate.

REVIEWER COMMENT:

3. It is not clear what the determinant of the variance is? Please provide additional details.

RESPONSE:

We are interested in the scatter of reach endpoints without assuming that the “interesting” dispersion occurs along any particular direction (x, y, z, or any linear combination of them). We could have calculated the variance along the x, y, and z directions and summed them. However, this would not account for correlations between movement along different directions (e.g., if movement in the x direction is correlated with movement along the y axis). Generalized variance provides a measure of the dispersion of a volume of points - the volume of the ellipsoid defined by the covariance matrix is proportional to the square root of the generalized variance. We rewrote the relevant section in Methods (fourth paragraph in Materials and Methods-->Processing Reach Kinematics) to clarify how the generalized variance was calculated and its meaning.

REVIEWER COMMENT:

4. Fig 4C/G/K. Please show a cartoon and then actual image. It is hard to tell what metric is being used from the actual image.

RESPONSE:

We have included both cartoon and actual images to demonstrate how each kinematic metric is measured.

REVIEWER COMMENT:

5. Discussion: Please note that the Lemke et al paper found an effect of grasp timing and striatal activity for reach-to-grasp coordination. More specifically, with learning, there was an increase in inter-trial coherence of grasp onset timing to striatal theta (Fig 2d). This is likely important for reach-to-grasp coordination and timing of grasping.

RESPONSE:

We added a statement to the end of the second paragraph of the Discussion where we speculate on sources on inter-rat variability in skilled reaching, and the end of the third paragraph in the Discussion where we consider how the timing of transport and grasp phases are regulated.

REVIEWER COMMENT:

Reviewer #2

The paper applies newly developed methods to carrying out a detailed analysis of the fine hand movements acquired during skilled reaching in rats. The methods used in the current experiment have been previously applied to automated testing of rats on skilled reaching, and the current study provides additional details about this approach. The ability to track 3D trajectories for not only the hand, but individual digits in an automated reaching task is an extremely valuable contribution to the field, and the current study provides novel measures of the fine kinematic details during skill acquisition. The study is in general clearly written, and supported by informative figures. The main criticism of the study is that perhaps because of the low number of subjects included, the variability in performance makes one question whether some of the interpretations of the data are accurate. Specifically, the notion that digit kinematics continue to be refined after the gross motor skill of reaching is stabilized is not completely apparent from the data. Specific comments include:

- Methods on P5 indicate video recording started when paw was in ROI? Where is this ROI relative to the paw passing through the front slot? Is some of the movement not being captured as it happens before recording trigger?

RESPONSE:

The video is triggered by the paw passing through the front slot, however, we acquire video frames using a rolling buffer so that 300 frames pre-trigger and 1000 frames post-trigger are saved in the video. This allows the entire reaching movement to be captured (from the lift of the paw through pellet consumption) on the majority of trials. We revised the Methods section to clarify that video frames prior to the trigger event could be captured (second paragraph in Materials and Methods-->Skilled reaching-->Automated reaching system). Additionally, we added a reference to Figure 1A (first paragraph in Materials and Methods-->Skilled reaching-->Automated reaching system) which shows where the ROI is located in the side mirror view.

REVIEWER COMMENT:

- From the description of within session kinematics, it appears that a rat that performed 35 trials would contribute the same amount of data as a rat that performed 40 trials. This seems inherently incorrect, even when data are averaged over 10 consecutive trials as the authors indicate. Do the results hold if the authors take the more conservative approach and analyze only 35 trials from all subjects?

RESPONSE:

We understand the reviewer's concerns, however this analysis is difficult to do as the number of trials performed per session is variable among rats. Therefore, the analysis would be limited by the rat who performed the fewest number of trials (sometimes as few as 10), and the number of trials would change significantly between sessions and groups (learners vs non-learners). Because of these issues we felt that the best approach was to present the data as originally submitted.

The logic behind this approach may be seen most clearly by considering Figure 4E (within-session changes in aperture). Suppose rat A has a consistently small hand aperture and rat B has a consistently large hand aperture. If rat A performs 35 trials and rat B performs 40, there will be an artificial jump in mean paw aperture when rat A drops out, obscuring a group trend. However, it seems wrong to exclude the rest of rat B's data. More importantly, the analysis would be limited by the rat performing the fewest number of trials - all data past that point from other rats would have to be excluded. On the other hand, it seems wrong (and bias-inducing) to entirely exclude the rat performing the fewest number of trials. We believe the best compromise is to carry the last data point forward as rats drop out of the analysis.

REVIEWER COMMENT:

- Authors refer to the paw of the rat, however Whishaw has clearly outlined reasons why rats and mice have hands, not paws. The authors should probably call it a hand consistently throughout the manuscript and figures.

RESPONSE:

We now refer to the paw of the rat as a hand throughout the manuscript and figures.

REVIEWER COMMENT:

- Fig 2A: Which trials are plotted? All successful reaches from all rats?

RESPONSE:

These are all reaches, whether successful or not. This has been clarified in the caption.

REVIEWER COMMENT:

- Fig 2D: difficult to discern that x axis labels trial numbers 15 and 30? First read as 1530...

o Related to previous point, did all rats in this graph perform at least 30 trials, or was data extrapolated as described in methods?

RESPONSE:

We have spread out the x axis labels “15” and “30” so that they are clearly separated. In Figure 4 panels E, I, and M (which are slightly compressed compared to similar panels elsewhere), the “15” has been removed entirely. These data were extrapolated as described in the methods.

REVIEWER COMMENT:

- Images in Fig 3A could be labeled with “Side (mirror) view” and “front view” for clarity

RESPONSE:

We added the labels “side mirror view” and “direct view” to the images in Figure 3A.

REVIEWER COMMENT:

- Data in Figure 3 suggest there is decrease in digit variability after initial few training sessions, and data also show that end-point variability of hand position is lower than digit position. Given that these conclusions are based on data from DLC one may wonder if the accuracy and consistency of the markerless tracking is different between the hand and the digits. This could explain some of the effects. In addition, increased labeling variability may also be introduced if the posture of the hand is different from what the network was trained on ie. the network may have been optimized for accurately detecting the hand in latter training sessions, and therefore performs more variably during initial testing sessions. It may be that the rats have a different (but equally consistent) hand posture early in training. Could the authors compare confidence values and the number of mislabelled frames from early vs. latter training to see if the networks performs inference equally well at these different timepoints? Similar analysis of labelling accuracy should be compared for hand vs. digit labels.

RESPONSE:

This is a good point. To address this, we calculated the average percentage of mislabeled frames for the hand and four digit tips on Days 1 and 10 (Figure 3E). With the exception of digit 4, the fraction of mislabeled frames was stable between days 1 and 10. However, the variability in digit 4 position decreased across sessions; if mislabeling caused a problem, we would expect digit 4 variability to increase. Furthermore, the number of mislabeled frames was comparable between the hand and digits for both sessions.

Mislabeled frames were determined using custom MATLAB code (see the last paragraph in Materials and Methods→Processing Reach Kinematics). Briefly, marked points were deemed “mislabeled” if below a threshold confidence value of 0.85 (produced by DLC). If the confidence value was above 0.97 the point was considered valid. If the confidence value was between 0.85 and 0.97, we then looked for points that made large 'spatial jumps' between frames (i.e., we expect a point can only move so far between consecutive frames so if it moves above a threshold we can assume at least one of the frames has been mislabeled).

REVIEWER COMMENT:

- The main interpretation of the data, that refinement of digit movements continues beyond when success rate and trajectory have stabilized is not fully supported by the data that's shown. For example, fig 2C shows that distance from average trajectory decreases, while success rate increases but these changes occur essentially at the same time. Perhaps the most direct effect to support the authors claim is in figure 4, where the angle of the digits (panel g-j) changed around the 6th day of training, while the authors claim that the increase in reaching performance had stabilized by that point. However the reaching accuracy does indeed fluctuate between sessions 6-10, and the changes in hand kinematics are partially overlapping with this time-course. Either toning back the claims on the sequential development of gross and fine motor skill or perhaps providing additional evidence to support these statements would be required.

RESPONSE:

Reviewing the aggregated data, we see Reviewer #2's point that changes in gross trajectories, fine digit movements, and reach success are not as robustly related as we suggested in the original text. We had been analyzing individual-level data in detail, where it is clearer that changes in gross and fine motor control occur at different times. To help the reader visualize this, we now include a “summary sheet” for each rat as Extended Data Figures 6-3 to 6-16. To see these differences in “gross” and “fine” movement stabilization, compare panel C (gross kinematics) to panels B and the right column of panel D.

While this is a lot of data to digest, we believe these plots help explain why the aggregated data do not clearly show sequential movement refinement as suggested by prior studies. First, success rates and kinematic measures do not necessarily change monotonically at the level of individual rats, making it difficult to identify the point at which any specific measure stabilizes. Even for rats in which success rates/kinematic measures change smoothly across days, the sessions in which they stabilize are different for each rat. Finally, each kinematic measure may not stabilize in the same session for each rat. For example, aperture might stabilize before paw orientation in one rat (e.g., rat I), but orientation stabilizes before aperture in another (e.g., rat N).

Reviewer #1's suggestion to divide rats into learners and non-learners also significantly altered our interpretations. As reviewer #1 suggested might be the case, it became clear to us that learners drive much of the changes in reach kinematics that we saw in the aggregated data. For example, rat “B” gross trajectories stabilized by day 3, while digit flexion/aperture, and digit endpoints with respect to the hand changed much later. For each learner, there was some feature of fine kinematics (aperture, orientation, flexion, or digit position with respect to the hand) that continued to change after success rate stabilized (but not always the same parameter). Additionally, most non-learners adapted their reach kinematics, though some did not adjust their kinematics much at all (e.g., rat L, for whom kinematics variability increased, if anything).

Collectively, these findings reinforce our interpretation that, for the most part, rats refine fine digit movements (reflected in hand shaping and digit endpoints with respect to the hand) after gross paw transport. Different rats may adapt different kinematic features in different sequences, however.
