# Supplementary figures and images for: Evolution of Gross Forelimb and Fine Digit Kinematics during Skilled Reaching Acquisition in Rats
Source: eNeuro. 2021 Oct 26;8(5):ENEURO.0153-21.2021. doi: 10.1523/ENEURO.0153-21.2021 (PMC8555885; doi:10.1523/ENEURO.0153-21.2021)

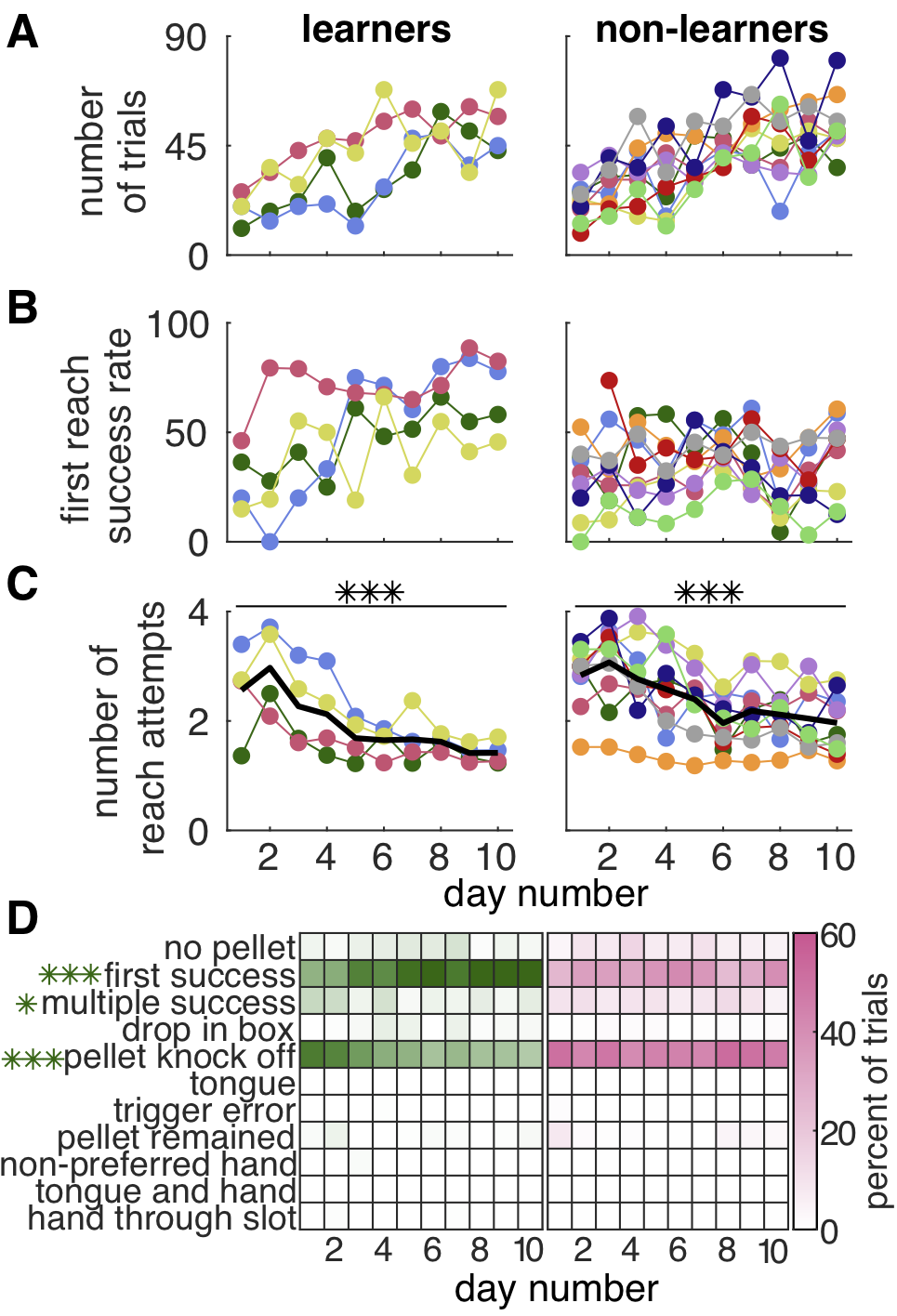

Supplement: Extended Data Figure 1-1 — Additional task performance measures. A, Individual rat data for average number of trials performed per day for learners (left) and non-learners (right). Each colored line represents an individual rat. Colors represent the same rat across all individual rat data figures within groups. B, Individual rat data for first reach success rate for learners (left) and non-learners (right). C, Number of reach attempts per trial. Both learners (left) and non-learners (right) decreased the number of reach attempts performed per trial over days (linear mixed model: effect of group: t(19) = 0.81, p = 0.43; effect of day: t(124) = –4.12, p = 6.88 × 10^−5^; group × day interaction: t(124) = 1.37, p = 0.17. Black line represents averaged data. Colored lines represent individual rats. D, Breakdown of trial outcomes by day for learners (green) and non-learners (for definitions of outcomes, see Materials and Methods, Number of trials and success rate). The percentage of “first success” increased over days only for learners (linear mixed model: effect of day: learners: t(35) = 4.96, p = 1.83 × 10^−5^; non-learners: t(88) = 0.51, p = 0.61). “Multiple success” outcomes decreased over days for learners but not non-learners (linear mixed model: effect of day: learners: t(35) = –2.53, p = 0.02; non-learners: t(89) = –0.79, p = 0.43). Similarly, “pellet knocked off” outcomes decreased for learners but not non-learners over days (linear mixed model: effect of day: learners: t(35) = –4.59, p = 5.46 × 10^−5^; non-learners: t(89) = 0.49, p = 0.62). All other outcomes were consistent across days for both groups. Linear mixed model: effect of day (learners): no pellet: t(35) = –0.11, p = 0.91; drop in box; t(35) = 0.42, p = 0.68; tongue: t(35) = 0; p = 1; trigger error: t(38) = –0.52, p = 0.61; pellet remained: t(38) = –0.78, p = 0.44; non-preferred hand: t(38) = –0.87, p = 0.39; tongue and hand: t(38) = 0, p = 1; hand through slot: t(38) = –0.78, p = 0.44. Linear mixed model: effect of day (non-learners [file enu-eN-NWR-0153-21-s02.tif]

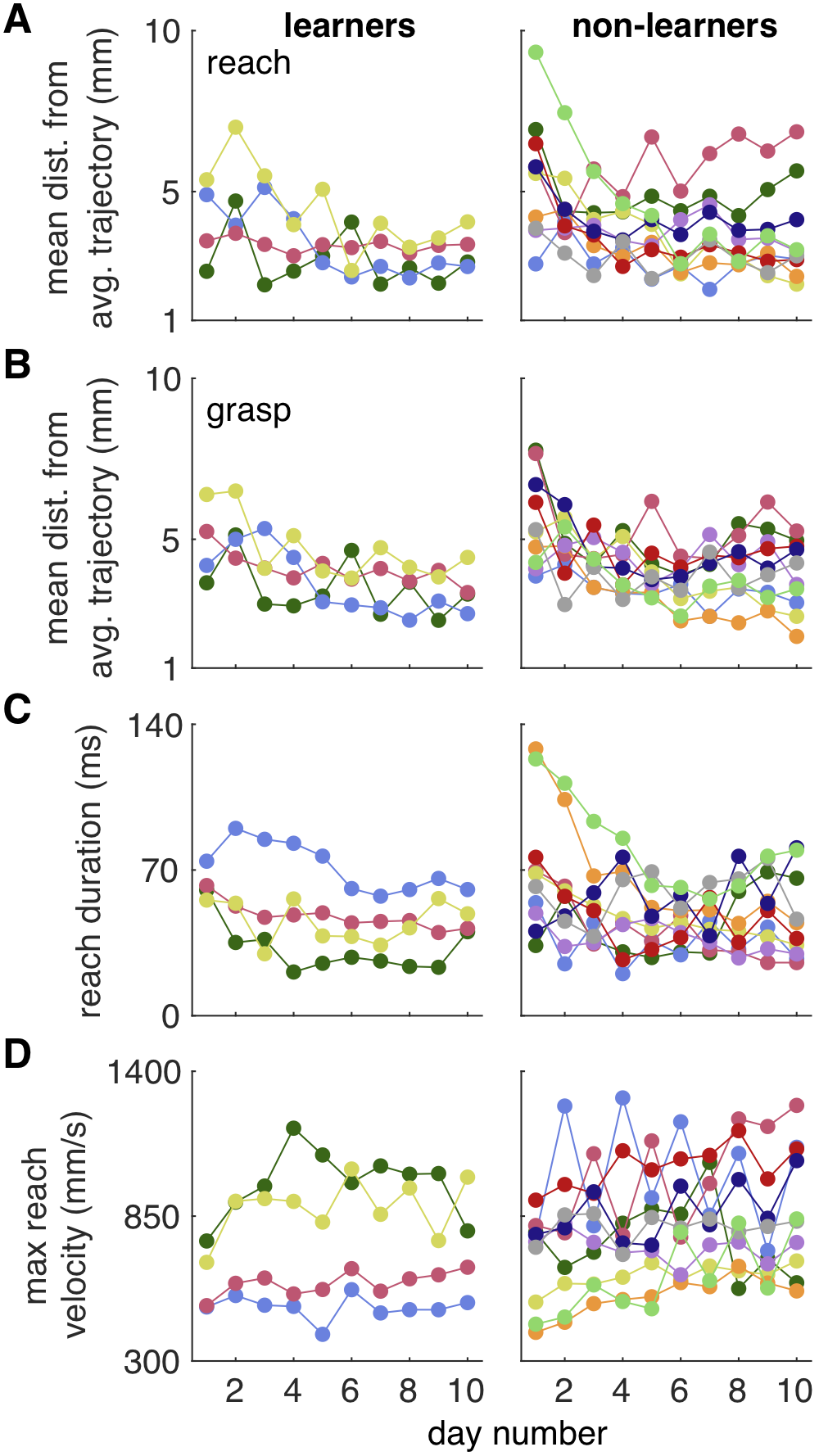

Supplement: Extended Data Figure 2-1 — Individual rat data for hand trajectory variability, reach duration, and reach velocity. A, Average hand trajectory variability of the reach component for learners (left) and non-learners (right) represented as the mean distance from the average trajectory (mm). B, Average hand trajectory variability of the grasp component for learners (left) and non-learners (right) represented as the mean distance from the average trajectory (mm). C, Average reach duration (ms) for learners (left) and non-learners (right). D, Maximum reach velocity (mm/s) for learners (left) and non-learners (right). Data and code to generate this figure are contained in Extended Data 1, 2. Download Figure 2-1, TIF file. [file enu-eN-NWR-0153-21-s03.tif]

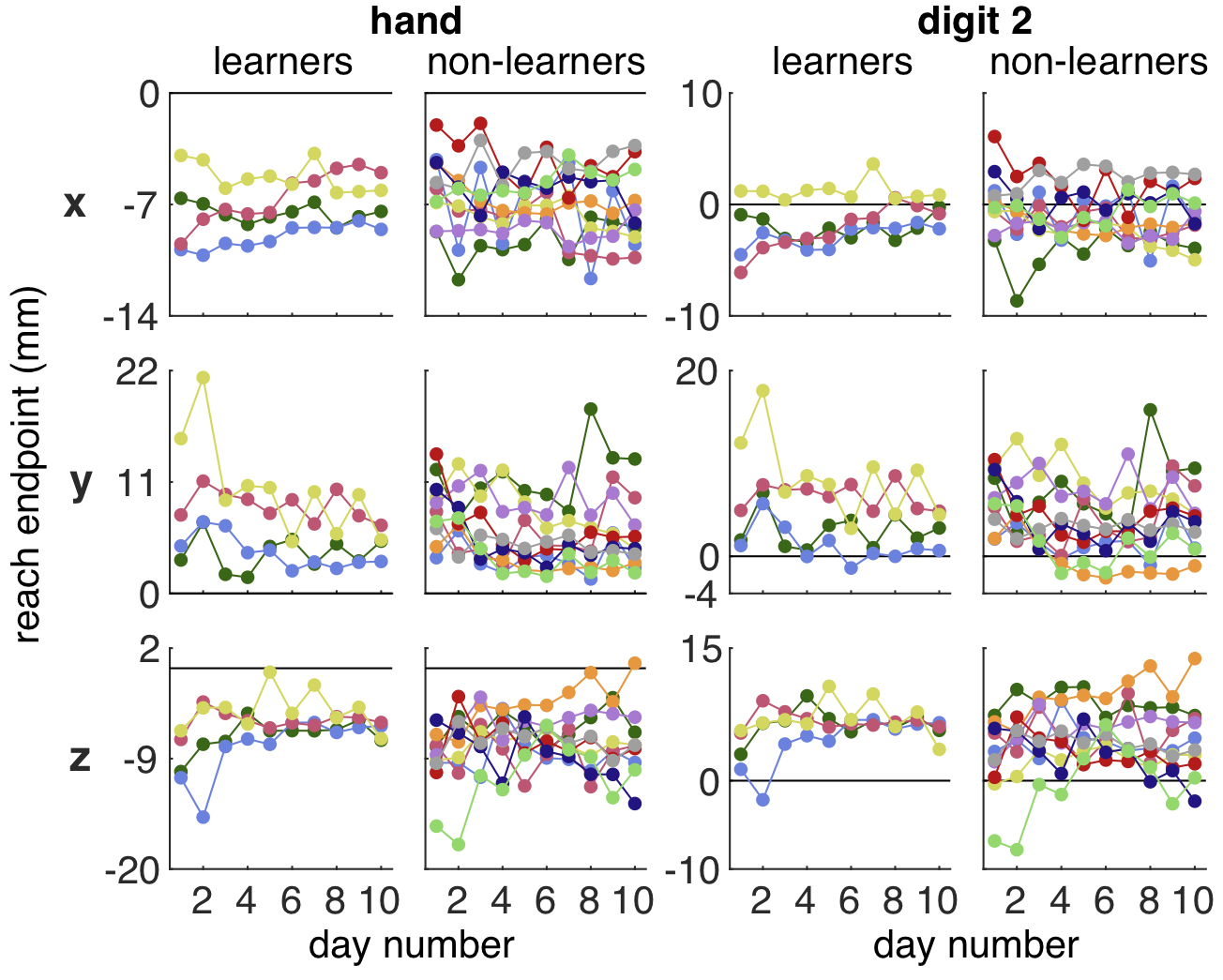

Supplement: Extended Data Figure 3-1 — Individual rat data for reach endpoints. Average reach endpoints of the hand (left) and digit 2 (right) for learners (left columns) and non-learners (right columns) in the X, Y, and Z directions. Pellet is at (0,0,0). Data and code to generate this figure are contained in Extended Data 1, 2. Download Figure 3-1, TIF file. [file enu-eN-NWR-0153-21-s04.tif]

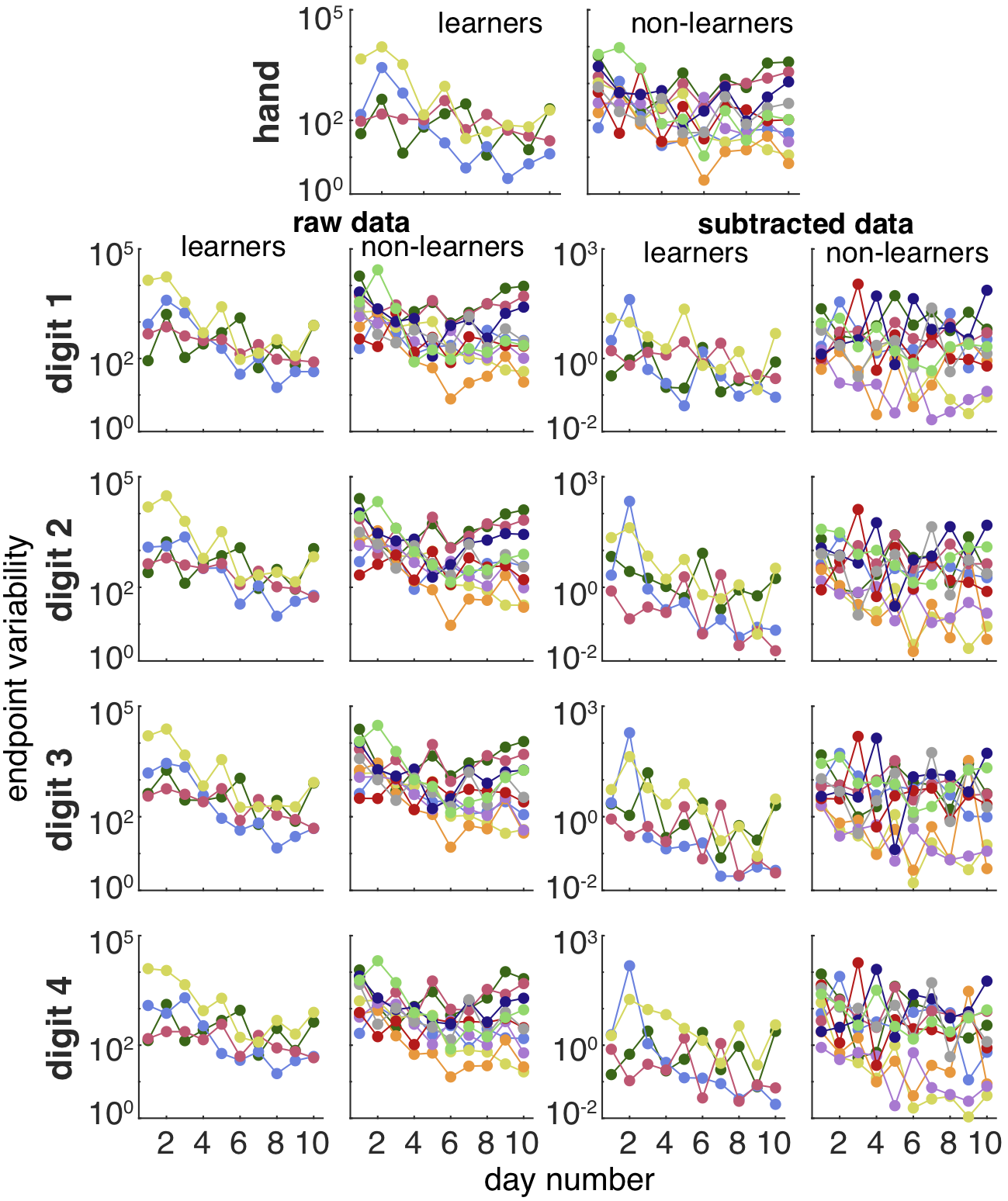

Supplement: Extended Data Figure 3-2 — Individual rat data for endpoint variability. Average determinant of the covariance matrix (generalized variance) of reach endpoints for the hand and digits. For digits, left two columns show endpoint variability of “raw” digit positions and right two columns show endpoint variability of digit positions subtracted from the hand position. Data and code to generate this figure are contained in Extended Data 1, 2. Download Figure 3-2, TIF file. [file enu-eN-NWR-0153-21-s05.tif]

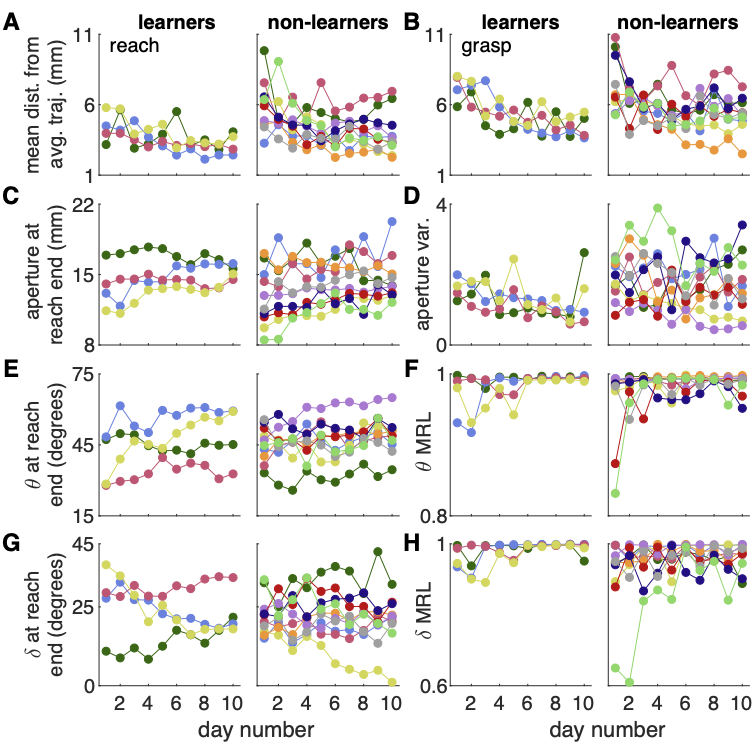

Supplement: Extended Data Figure 4-1 — Individual rat fine digit kinematics data. A, Average digit 2 trajectory variability of the reach component for individual rats represented as the mean distance from the average trajectory. B, Average digit 2 trajectory variability of the grasp component for individual rats represented as the mean distance from the average trajectory. C, Average aperture at reach end (mm) for individual rats. D, Average aperture variance at reach end for individual rats. E, Average hand orientation (degrees) at reach end for individual rats. F, Average hand orientation MRL at reach end for individual rats. G, Average digit flexion (degrees) at reach end for individual rats. H, Average digit flexion MRL at reach end for individual rats. Data and code to generate this figure are contained in Extended Data 1, 2. Download Figure 4-1, TIF file. [file enu-eN-NWR-0153-21-s06.tif]

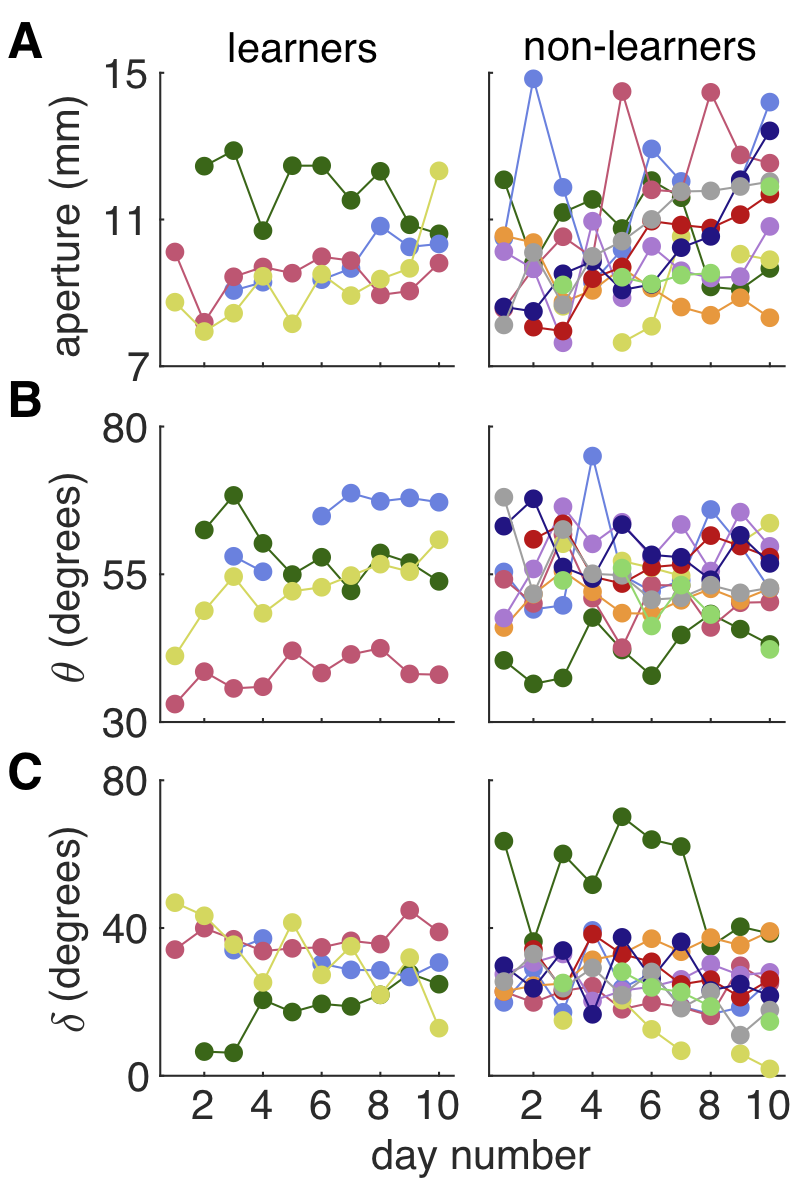

Supplement: Extended Data Figure 5-1 — Digit and forelimb coordination individual rat data. A, Average grasp aperture at the zdigit2 coordinate (+3 mm) indicated by the dashed line in Figure 5A as a function of day number for individual rats (left learners; right non-learners). B, Average hand orientation (degrees) at the zdigit2 coordinate (+3 mm) indicated by the dashed line in Figure 5C as a function of day number for individual rats. C, Average digit flexion (degrees) at the zdigit2 coordinate (+3 mm) indicated by the dashed line in Figure 5E as a function of day number for individual rats. Data and code to generate this figure are contained in Extended Data 1, 2. Download Figure 5-1, TIF file. [file enu-eN-NWR-0153-21-s07.tif]

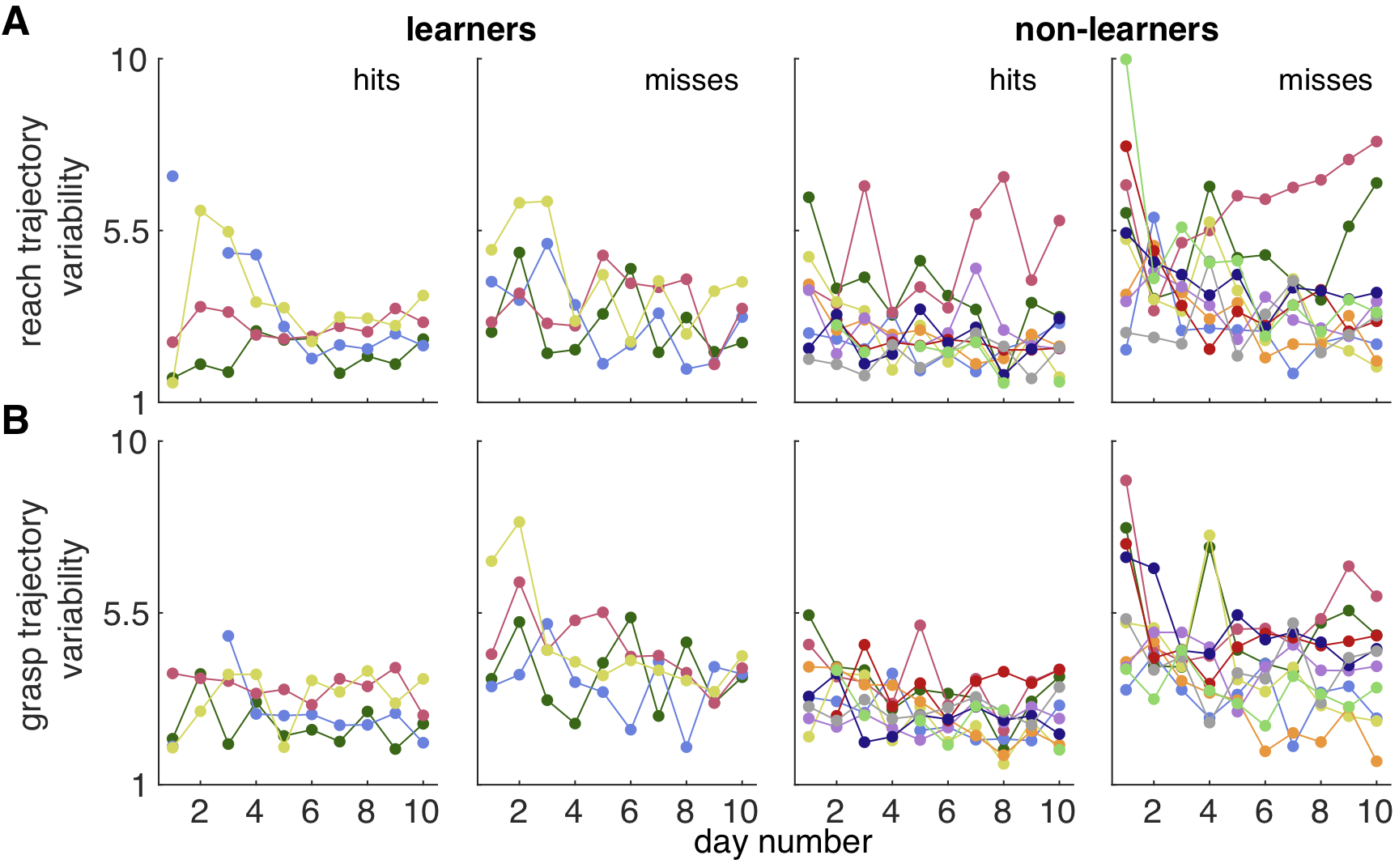

Supplement: Extended Data Figure 6-1 — Trajectory variability by outcome for individual rats. A, Average hand trajectory variability of the reach component for successful reaches (“hits,” left column) and unsuccessful reaches (“misses,” right column) for individual learner (left) and non-learner (right) rats. B, Average hand trajectory variability of the grasp component for successful reaches (“hits,” left column) and unsuccessful reaches (“misses,” right column) for individual learner (left) and non-learner (right) rats. Data and code to generate this figure are contained in Extended Data 1, 2. Download Figure 6-1, TIF file. [file enu-eN-NWR-0153-21-s08.tif]

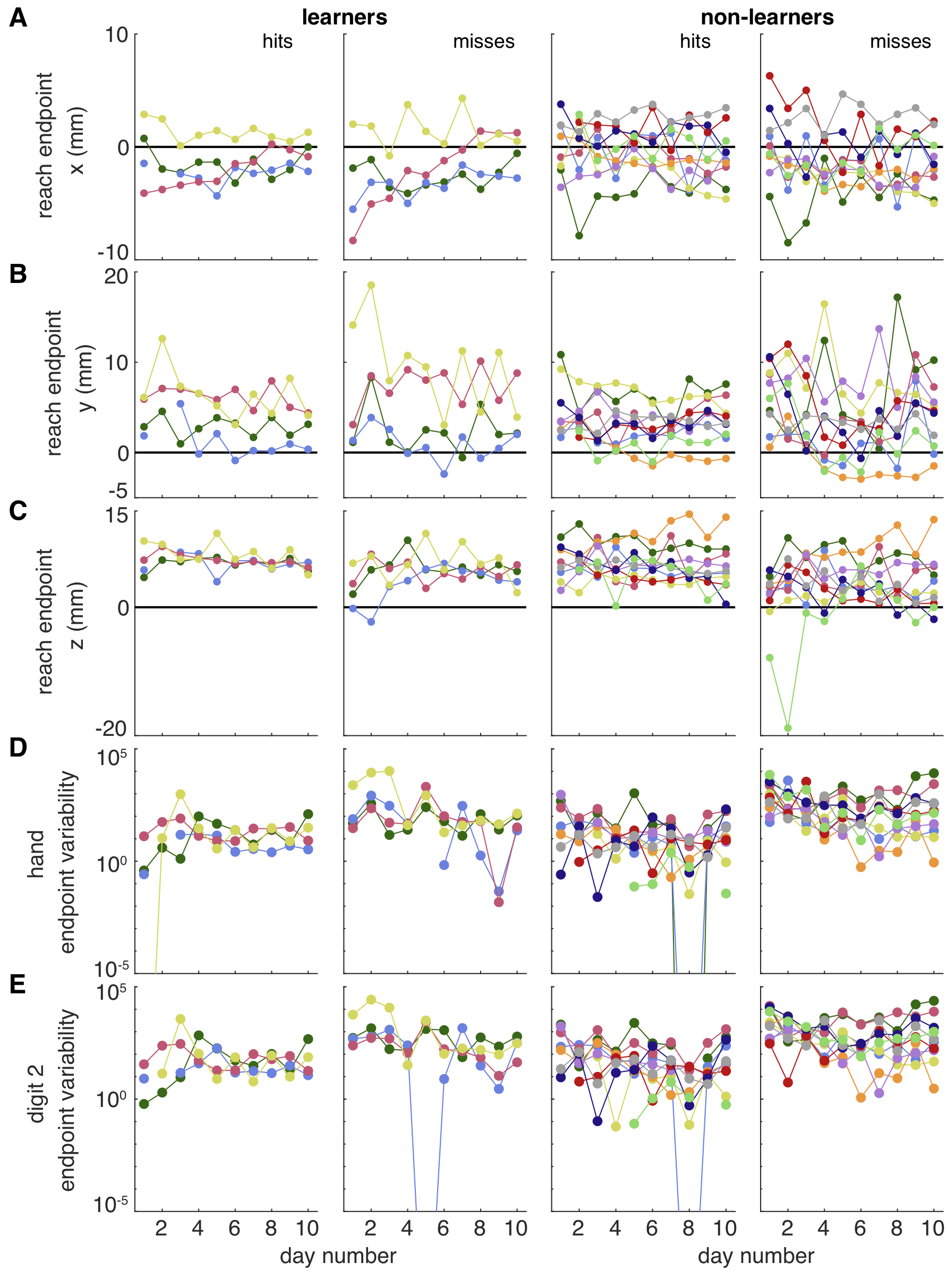

Supplement: Extended Data Figure 6-2 — Reach endpoint by outcome for individual rats. A, Average reach endpoint (X direction) for successful reaches (“hits”, left column) and unsuccessful reaches (“misses”, right column) for individual learner (left) and non-learner (right) rats. B, Average reach endpoint (Y direction) for successful reaches (“hits”, left column) and unsuccessful reaches (“misses”, right column) for individual learner (left) and non-learner (right) rats. C, Average reach endpoint (Z direction) for successful reaches (“hits”, left column) and unsuccessful reaches (“misses”, right column) for individual learner (left) and non-learner (right) rats. D, Average reach endpoint variability of the hand for successful reaches (“hits”, left column) and unsuccessful reaches (“misses”, right column) for individual learner (left) and non-learner (right) rats. E, Average reach endpoint variability of digit 2 for successful reaches (“hits,” left column) and unsuccessful reaches (“misses,” right column) for individual learner (left) and non-learner (right) rats. Data and code to generate this figure are contained in Extended Data 1, 2. Download Figure 6-2, TIF file. [file enu-eN-NWR-0153-21-s09.tif]

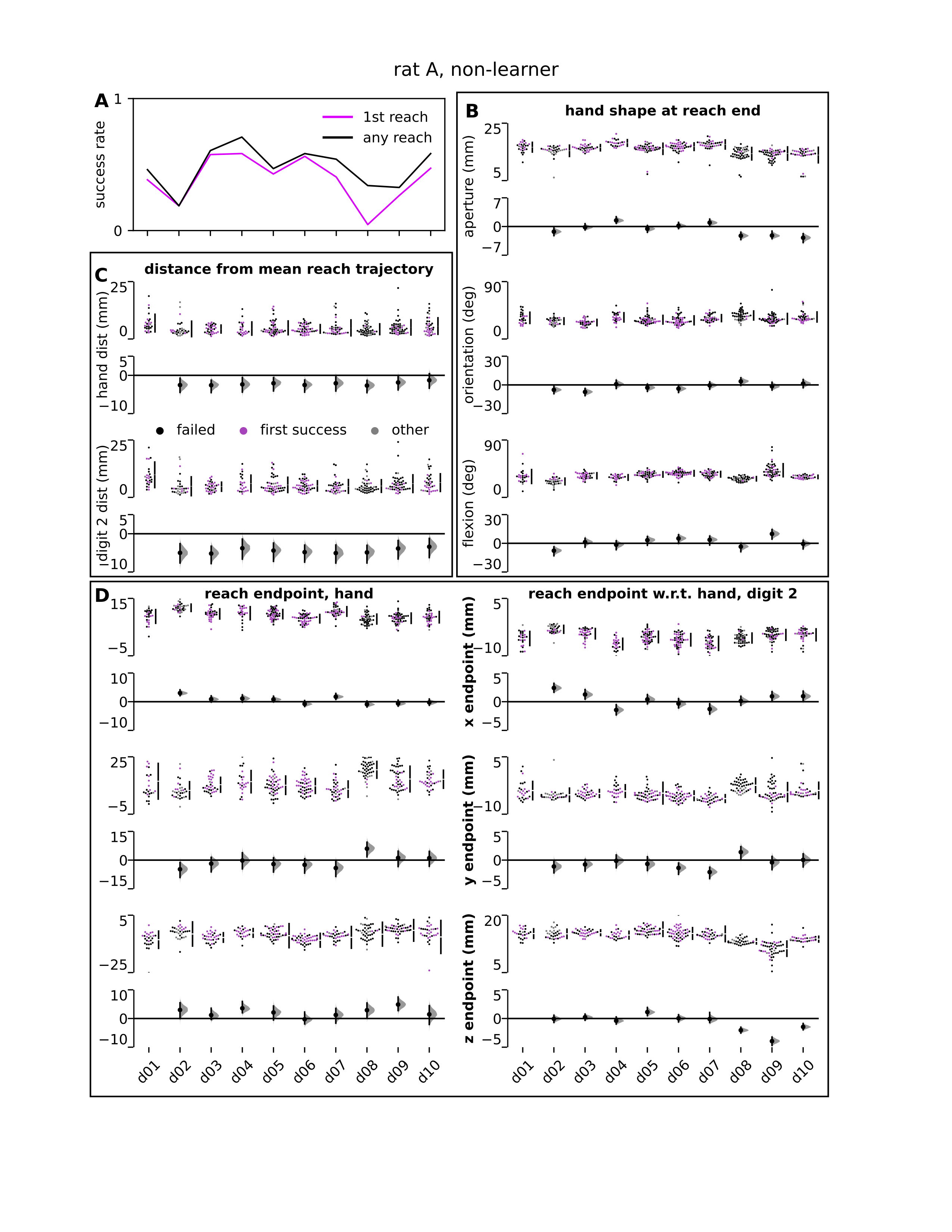

Supplement: Extended Data Figure 6-3 — Kinematics summary sheet for a nonlearner rat. A, Success rate across days. Pink lines indicate first reach success, black lines indicate success on any reach attempt for a single trial. B, Shared control plots illustrating measures of hand shaping at reach end (all data for a single rat that went into Fig. 4C–N). The top axes are swarm plots showing aperture at reach end for every trial. Pink dots indicate first-reach success trials, black dots indicate first reach failed trials (i.e., pellet remained, pellet knocked off, or multiple reach success), and gray dots indicate all other trials (e.g., no pellet delivered). Bottom plots show the difference between the mean value on each day and the mean value on day 1. Distributions show the results of a bootstrap resampling procedure with 95% confidence intervals indicated by the solid lines at the left of each distribution. C, Same as B for the mean distance from the average reach trajectory for each day (top panel shows all hand location data for a single rat that went into Fig. 2C, left panel; bottom panel shows all digit 2 location data that went into Fig. 4A, left panel). D, Same as B, C for the reach endpoint analyses. Left column shows reach endpoints in x, y, and z for the hand location (all data for a single rat that went into Fig. 3B, left panels). Right column shows reach endpoints for digit 2 with respect to the hand location (all data for a single rat that went into Fig. 3B, right panels, except here the hand location was subtracted out). Data and code to generate this figure are contained in Extended Data 1, 2, 3. Download Figure 6-3, TIF file. [file enu-eN-NWR-0153-21-s10.tif]

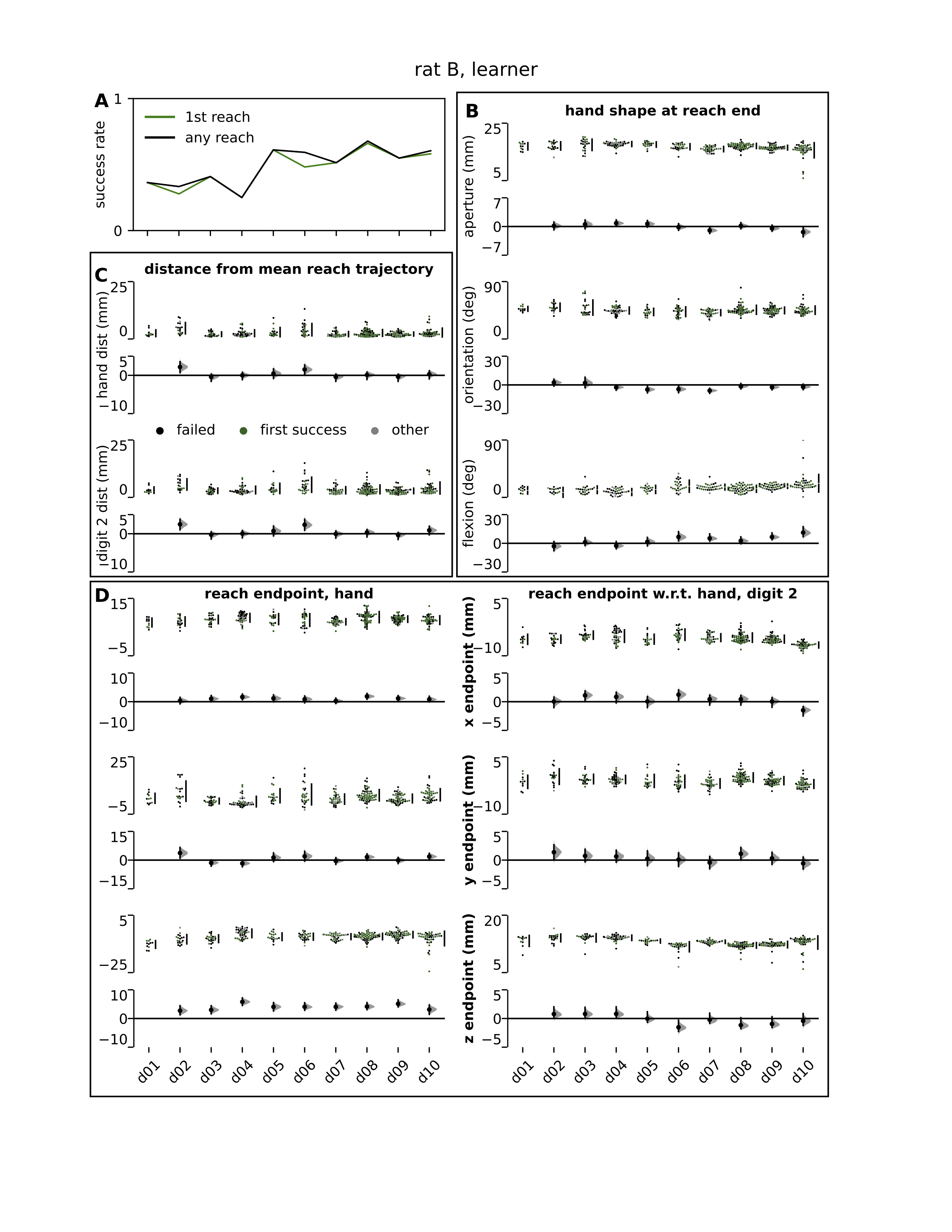

Supplement: Extended Data Figure 6-4 — Kinematics summary sheet for a learner rat. A, Success rate across days. Green lines indicate first reach success, black lines indicate success on any reach attempt for a single trial. B, Shared control plots illustrating measures of hand shaping at reach end (all data for a single rat that went into Fig. 4C–N). The top axes are swarm plots showing aperture at reach end for every trial. Green dots indicate first-reach success trials, black dots indicate first reach failed trials (i.e., pellet remained, pellet knocked off, or multiple reach success), and gray dots indicate all other trials (e.g., no pellet delivered). Bottom plots show the difference between the mean value on each day and the mean value on day 1. Distributions show the results of a bootstrap resampling procedure with 95% confidence intervals indicated by the solid lines at the left of each distribution. C, Same as B for the mean distance from the average reach trajectory for each day (top panel shows all hand location data for a single rat that went into Fig. 2C, left panel; bottom panel shows all digit 2 location data that went into Fig. 4A, left panel). D, Same as B, C for the reach endpoint analyses. Left column shows reach endpoints in x, y, and z for the hand location (all data for a single rat that went into Fig. 3B, left panels). Right column shows reach endpoints for digit 2 with respect to the hand location (all data for a single rat that went into Fig. 3B, right panels, except here the hand location was subtracted out). Data and code to generate this figure are contained in Extended Data 1, 2, 3. Download Figure 6-4, TIF file. [file enu-eN-NWR-0153-21-s11.tif]

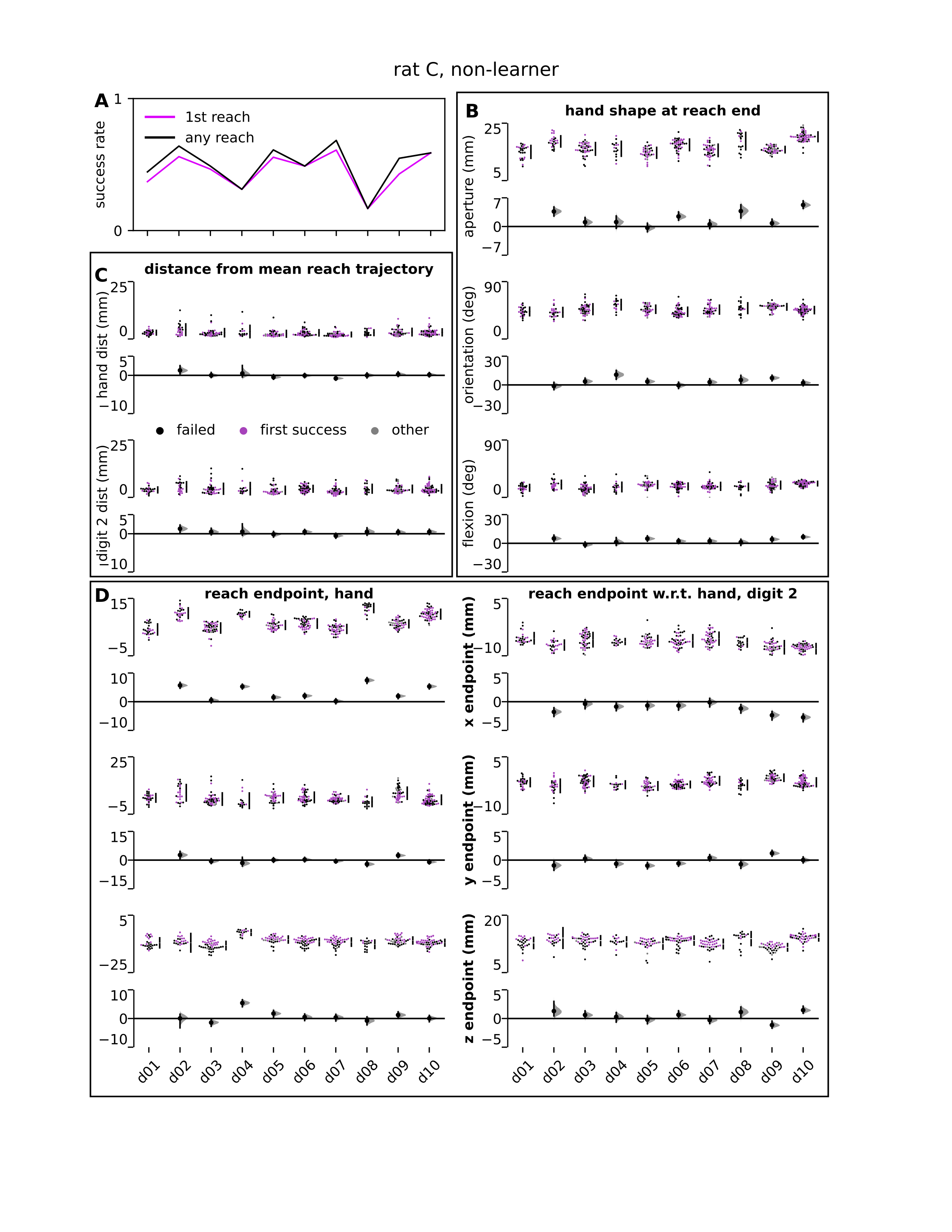

Supplement: Extended Data Figure 6-5 — Kinematics summary sheet for a nonlearner rat. A, Success rate across days. Pink lines indicate first reach success, black lines indicate success on any reach attempt for a single trial. B, Shared control plots illustrating measures of hand shaping at reach end (all data for a single rat that went into Fig. 4C–N). The top axes are swarm plots showing aperture at reach end for every trial. Pink dots indicate first-reach success trials, black dots indicate first reach failed trials (i.e., pellet remained, pellet knocked off, or multiple reach success), and gray dots indicate all other trials (e.g., no pellet delivered). Bottom plots show the difference between the mean value on each day and the mean value on day 1. Distributions show the results of a bootstrap resampling procedure with 95% confidence intervals indicated by the solid lines at the left of each distribution. C, Same as B for the mean distance from the average reach trajectory for each day (top panel shows all hand location data for a single rat that went into Fig. 2C, left panel; bottom panel shows all digit 2 location data that went into Fig. 4A, left panel). D, Same as B, C for the reach endpoint analyses. Left column shows reach endpoints in x, y, and z for the hand location (all data for a single rat that went into Fig. 3B, left panels). Right column shows reach endpoints for digit 2 with respect to the hand location (all data for a single rat that went into Fig. 3B, right panels, except here the hand location was subtracted out). Data and code to generate this figure are contained in Extended Data 1, 2, 4. Download Figure 6-5, TIF file. [file enu-eN-NWR-0153-21-s12.tif]

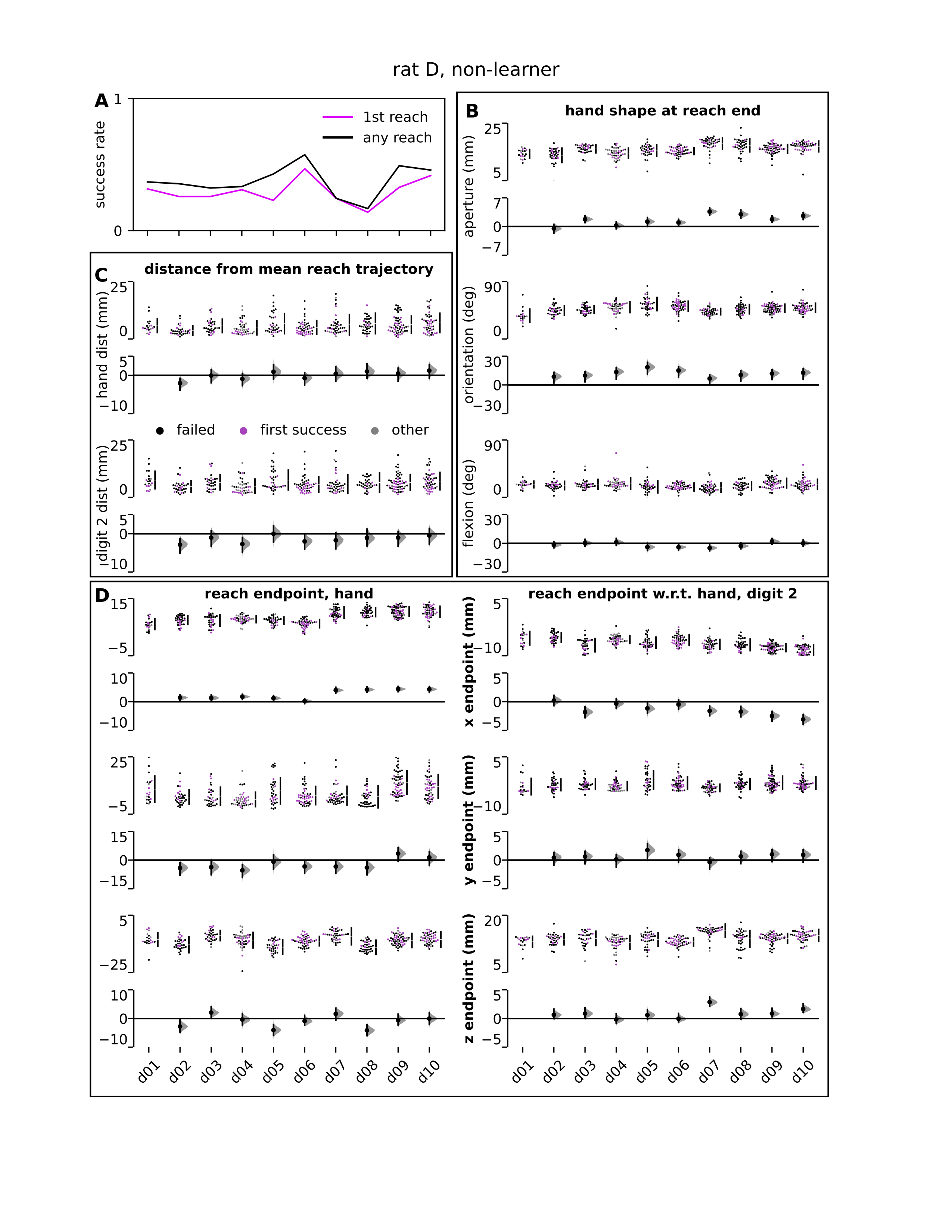

Supplement: Extended Data Figure 6-6 — Kinematics summary sheet for a nonlearner rat. A, Success rate across days. Pink lines indicate first reach success, black lines indicate success on any reach attempt for a single trial. B, Shared control plots illustrating measures of hand shaping at reach end (all data for a single rat that went into Fig. 4C–N). The top axes are swarm plots showing aperture at reach end for every trial. Pink dots indicate first-reach success trials, black dots indicate first reach failed trials (i.e., pellet remained, pellet knocked off, or multiple reach success), and gray dots indicate all other trials (e.g., no pellet delivered). Bottom plots show the difference between the mean value on each day and the mean value on day 1. Distributions show the results of a bootstrap resampling procedure with 95% confidence intervals indicated by the solid lines at the left of each distribution. C, Same as B for the mean distance from the average reach trajectory for each day (top panel shows all hand location data for a single rat that went into Fig. 2C, left panel; bottom panel shows all digit 2 location data that went into Fig. 4A, left panel). D, Same as B, C for the reach endpoint analyses. Left column shows reach endpoints in x, y, and z for the hand location (all data for a single rat that went into Fig. 3B, left panels). Right column shows reach endpoints for digit 2 with respect to the hand location (all data for a single rat that went into Fig. 3B, right panels, except here the hand location was subtracted out). Data and code to generate this figure are contained in Extended Data 1, 2, 4. Download Figure 6-6, TIF file. [file enu-eN-NWR-0153-21-s13.tif]

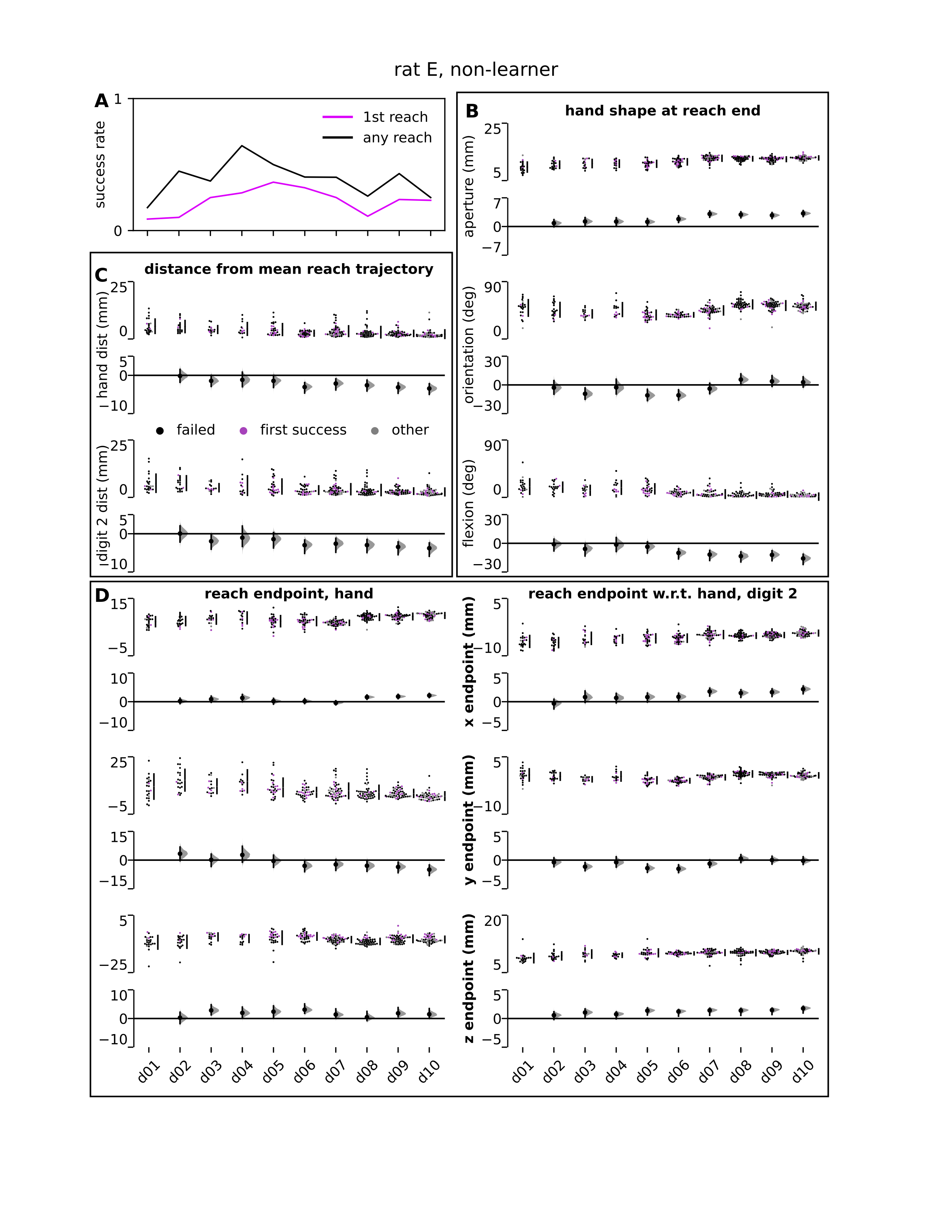

Supplement: Extended Data Figure 6-7 — Kinematics summary sheet for a nonlearner rat. A, Success rate across days. Pink lines indicate first reach success, black lines indicate success on any reach attempt for a single trial. B, Shared control plots illustrating measures of hand shaping at reach end (all data for a single rat that went into Fig. 4C–N). The top axes are swarm plots showing aperture at reach end for every trial. Pink dots indicate first-reach success trials, black dots indicate first reach failed trials (i.e., pellet remained, pellet knocked off, or multiple reach success), and gray dots indicate all other trials (e.g., no pellet delivered). Bottom plots show the difference between the mean value on each day and the mean value on day 1. Distributions show the results of a bootstrap resampling procedure with 95% confidence intervals indicated by the solid lines at the left of each distribution. C, Same as B for the mean distance from the average reach trajectory for each day (top panel shows all hand location data for a single rat that went into Fig. 2C, left panel; bottom panel shows all digit 2 location data that went into Fig. 4A, left panel). D, Same as B, C for the reach endpoint analyses. Left column shows reach endpoints in x, y, and z for the hand location (all data for a single rat that went into Fig. 3B, left panels). Right column shows reach endpoints for digit 2 with respect to the hand location (all data for a single rat that went into Fig. 3B, right panels, except here the hand location was subtracted out). Data and code to generate this figure are contained in Extended Data 1, 2, 5. Download Figure 6-7, TIF file. [file enu-eN-NWR-0153-21-s14.tif]

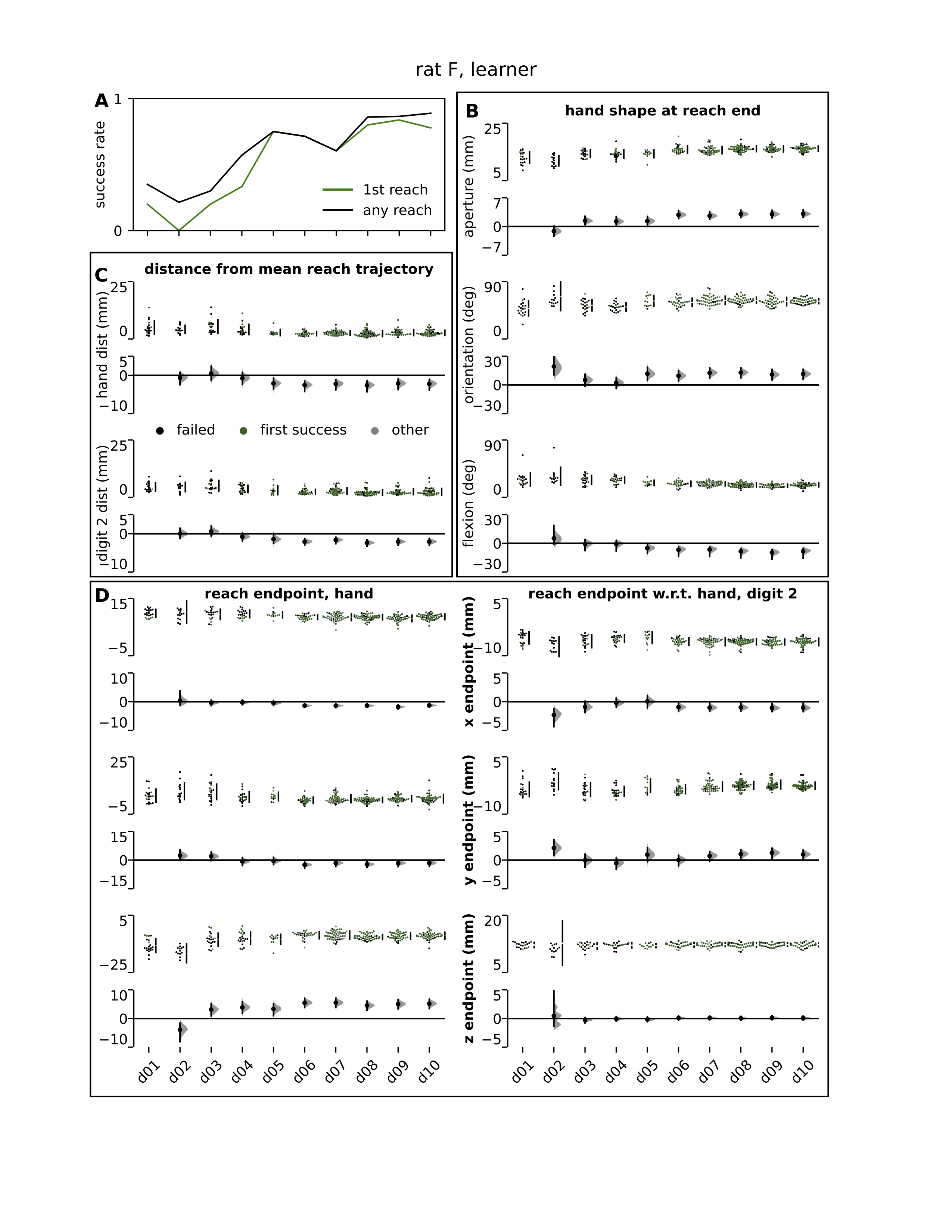

Supplement: Extended Data Figure 6-8 — Kinematics summary sheet for a learner rat. A, Success rate across days. Green lines indicate first reach success, black lines indicate success on any reach attempt for a single trial. B, Shared control plots illustrating measures of hand shaping at reach end (all data for a single rat that went into Fig. 4C–N). The top axes are swarm plots showing aperture at reach end for every trial. Green dots indicate first-reach success trials, black dots indicate first reach failed trials (i.e., pellet remained, pellet knocked off, or multiple reach success), and gray dots indicate all other trials (e.g., no pellet delivered). Bottom plots show the difference between the mean value on each day and the mean value on day 1. Distributions show the results of a bootstrap resampling procedure with 95% confidence intervals indicated by the solid lines at the left of each distribution. C, Same as B for the mean distance from the average reach trajectory for each day (top panel shows all hand location data for a single rat that went into Fig. 2C, left panel; bottom panel shows all digit 2 location data that went into Fig. 4A, left panel). D, Same as B, C for the reach endpoint analyses. Left column shows reach endpoints in x, y, and z for the hand location (all data for a single rat that went into Fig. 3B, left panels). Right column shows reach endpoints for digit 2 with respect to the hand location (all data for a single rat that went into Fig. 3B, right panels, except here the hand location was subtracted out). Data and code to generate this figure are contained in Extended Data 1, 2, 5. Download Figure 6-8, TIF file. [file enu-eN-NWR-0153-21-s15.tif]

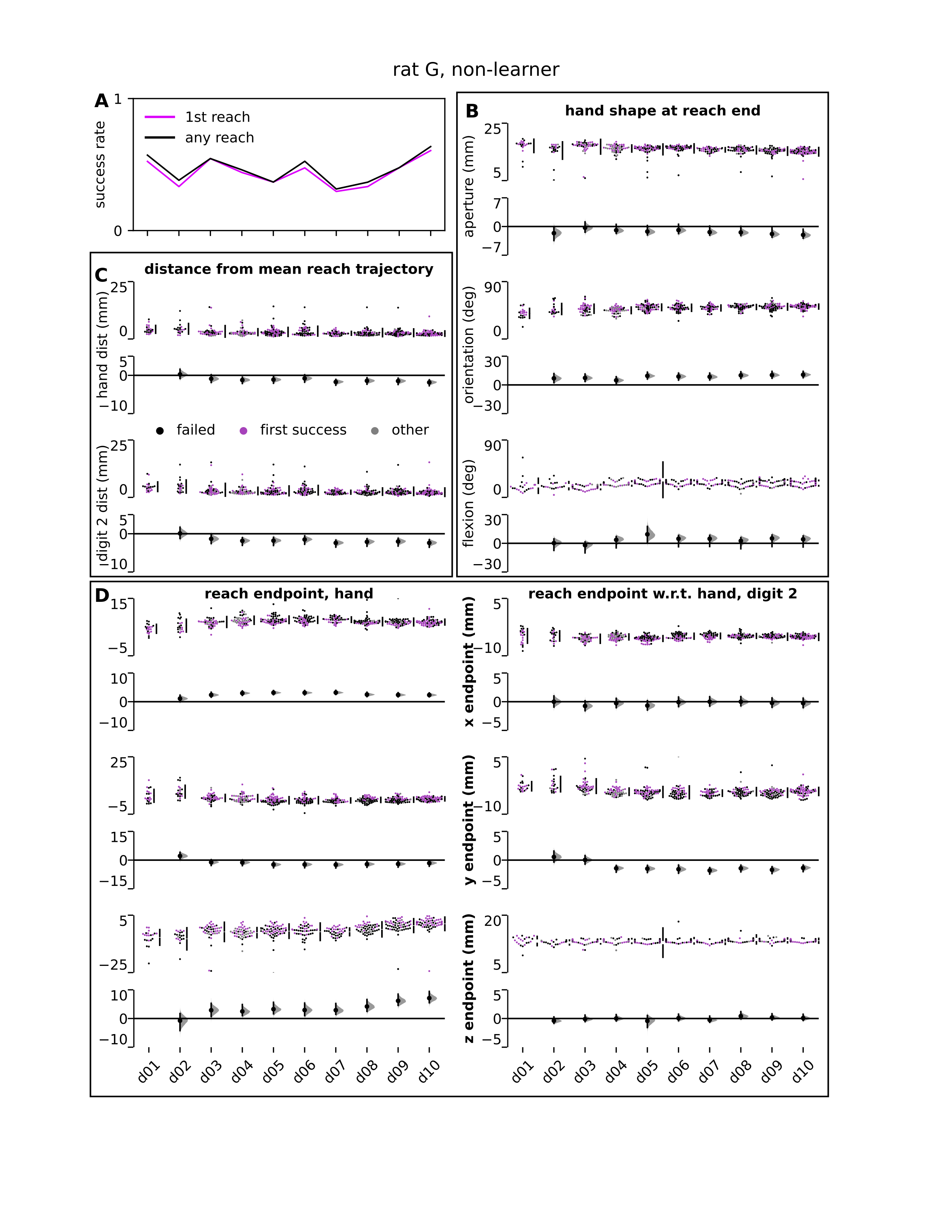

Supplement: Extended Data Figure 6-9 — Kinematics summary sheet for a nonlearner rat. A, Success rate across days. Pink lines indicate first reach success, black lines indicate success on any reach attempt for a single trial. B, Shared control plots illustrating measures of hand shaping at reach end (all data for a single rat that went into Fig. 4C–N). The top axes are swarm plots showing aperture at reach end for every trial. Pink dots indicate first-reach success trials, black dots indicate first reach failed trials (i.e., pellet remained, pellet knocked off, or multiple reach success), and gray dots indicate all other trials (e.g., no pellet delivered). Bottom plots show the difference between the mean value on each day and the mean value on day 1. Distributions show the results of a bootstrap resampling procedure with 95% confidence intervals indicated by the solid lines at the left of each distribution. C, Same as B for the mean distance from the average reach trajectory for each day (top panel shows all hand location data for a single rat that went into Fig. 2C, left panel; bottom panel shows all digit 2 location data that went into Fig. 4A, left panel). D, Same as B, C for the reach endpoint analyses. Left column shows reach endpoints in x, y, and z for the hand location (all data for a single rat that went into Fig. 3B, left panels). Right column shows reach endpoints for digit 2 with respect to the hand location (all data for a single rat that went into Fig. 3B, right panels, except here the hand location was subtracted out). Data and code to generate this figure are contained in Extended Data 1, 2, 6. Download Figure 6-9, TIF file. [file enu-eN-NWR-0153-21-s16.tif]

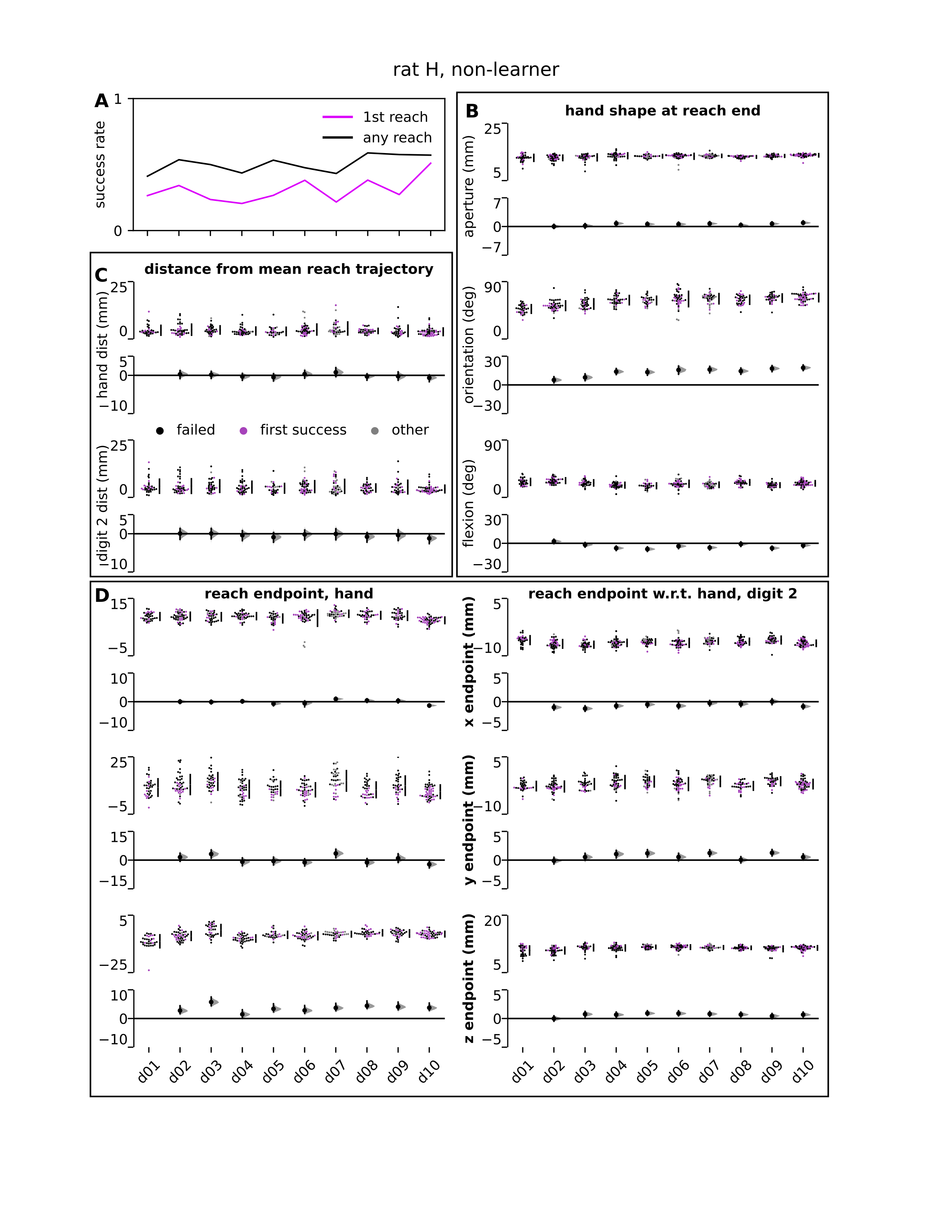

Supplement: Extended Data Figure 6-10 — Kinematics summary sheet for a nonlearner rat. A, Success rate across days. Pink lines indicate first reach success, black lines indicate success on any reach attempt for a single trial. B, Shared control plots illustrating measures of hand shaping at reach end (all data for a single rat that went into Fig. 4C–N). The top axes are swarm plots showing aperture at reach end for every trial. Pink dots indicate first-reach success trials, black dots indicate first reach failed trials (i.e., pellet remained, pellet knocked off, or multiple reach success), and gray dots indicate all other trials (e.g., no pellet delivered). Bottom plots show the difference between the mean value on each day and the mean value on day 1. Distributions show the results of a bootstrap resampling procedure with 95% confidence intervals indicated by the solid lines at the left of each distribution. C, Same as B for the mean distance from the average reach trajectory for each day (top panel shows all hand location data for a single rat that went into Fig. 2C, left panel; bottom panel shows all digit 2 location data that went into Fig. 4A, left panel). D, Same as B, C for the reach endpoint analyses. Left column shows reach endpoints in x, y, and z for the hand location (all data for a single rat that went into Fig. 3B, left panels). Right column shows reach endpoints for digit 2 with respect to the hand location (all data for a single rat that went into Fig. 3B, right panels, except here the hand location was subtracted out). Data and code to generate this figure are contained in Extended Data 1, 2, 7. Download Figure 6-10, TIF file. [file enu-eN-NWR-0153-21-s17.tif]

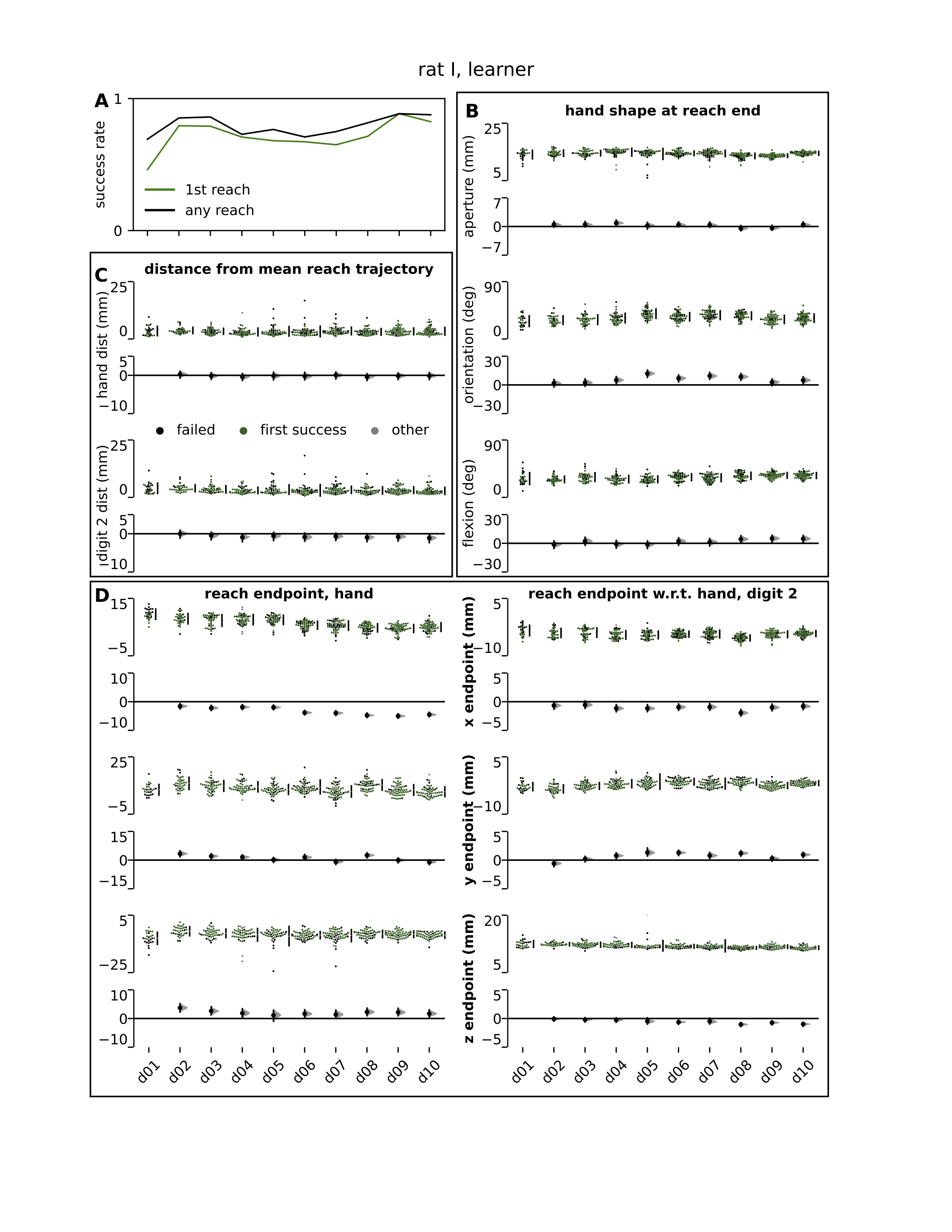

Supplement: Extended Data Figure 6-11 — Kinematics summary sheet for a learner rat. A, Success rate across days. Green lines indicate first reach success, black lines indicate success on any reach attempt for a single trial. B, Shared control plots illustrating measures of hand shaping at reach end (all data for a single rat that went into Fig. 4C–N). The top axes are swarm plots showing aperture at reach end for every trial. Green dots indicate first-reach success trials, black dots indicate first reach failed trials (i.e., pellet remained, pellet knocked off, or multiple reach success), and gray dots indicate all other trials (e.g., no pellet delivered). Bottom plots show the difference between the mean value on each day and the mean value on day 1. Distributions show the results of a bootstrap resampling procedure with 95% confidence intervals indicated by the solid lines at the left of each distribution. C, Same as B for the mean distance from the average reach trajectory for each day (top panel shows all hand location data for a single rat that went into Fig. 2C, left panel; bottom panel shows all digit 2 location data that went into Fig. 4A, left panel). D, Same as B, C for the reach endpoint analyses. Left column shows reach endpoints in x, y, and z for the hand location (all data for a single rat that went into Fig. 3B, left panels). Right column shows reach endpoints for digit 2 with respect to the hand location (all data for a single rat that went into Fig. 3B, right panels, except here the hand location was subtracted out). Data and code to generate this figure are contained in Extended Data 1, 2, 8. Download Figure 6-11, TIF file. [file enu-eN-NWR-0153-21-s18.tif]

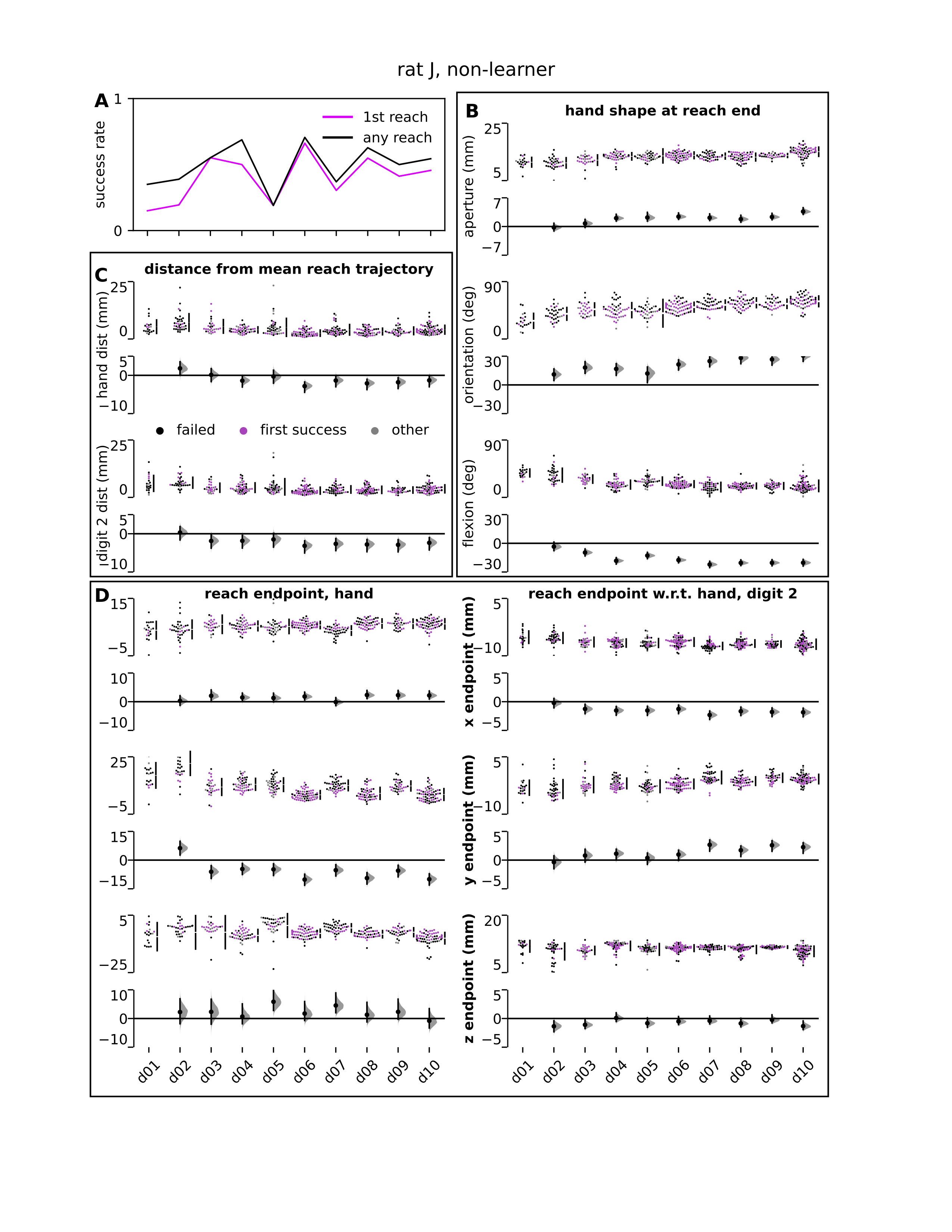

Supplement: Extended Data Figure 6-12 — Kinematics summary sheet for a nonlearner rat. A, Success rate across days. Pink lines indicate first reach success, black lines indicate success on any reach attempt for a single trial. A, Shared control plots illustrating measures of hand shaping at reach end (all data for a single rat that went into Fig. 4C–N). The top axes are swarm plots showing aperture at reach end for every trial. Pink dots indicate first-reach success trials, black dots indicate first reach failed trials (i.e., pellet remained, pellet knocked off, or multiple reach success), and gray dots indicate all other trials (e.g., no pellet delivered). Bottom plots show the difference between the mean value on each day and the mean value on day 1. Distributions show the results of a bootstrap resampling procedure with 95% confidence intervals indicated by the solid lines at the left of each distribution. C, Same as B for the mean distance from the average reach trajectory for each day (top panel shows all hand location data for a single rat that went into Fig. 2C, left panel; bottom panel shows all digit 2 location data that went into Fig. 4A, left panel). D, Same as B, C for the reach endpoint analyses. Left column shows reach endpoints in x, y, and z for the hand location (all data for a single rat that went into Fig. 3B, left panels). Right column shows reach endpoints for digit 2 with respect to the hand location (all data for a single rat that went into Fig. 3B, right panels, except here the hand location was subtracted out). Data and code to generate this figure are contained in Extended Data 1, 2, 9. Download Figure 6-12, TIF file. [file enu-eN-NWR-0153-21-s19.tif]

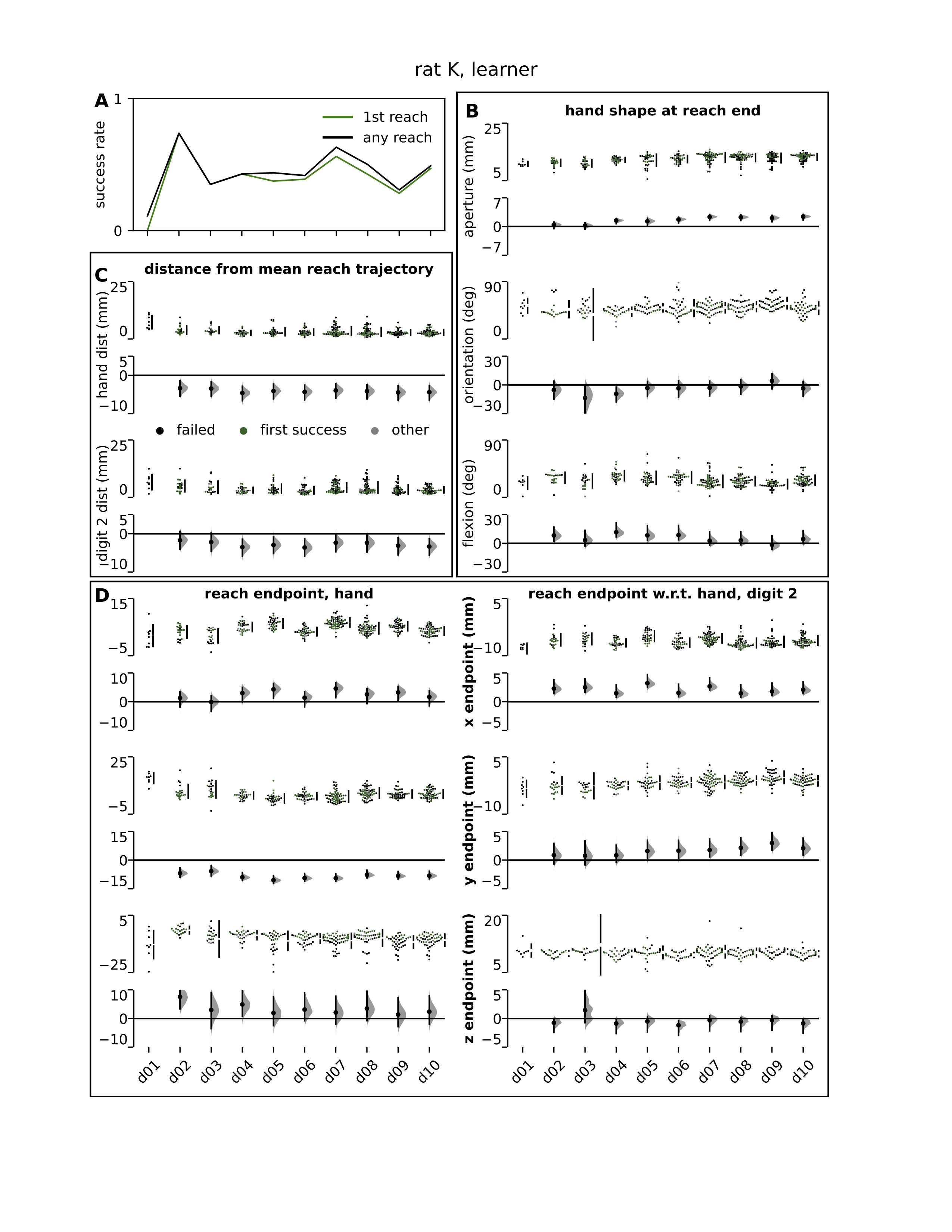

Supplement: Extended Data Figure 6-13 — Kinematics summary sheet for a learner rat. A, Success rate across days. Green lines indicate first reach success, black lines indicate success on any reach attempt for a single trial. B, Shared control plots illustrating measures of hand shaping at reach end (all data for a single rat that went into Fig. 4C–N). The top axes are swarm plots showing aperture at reach end for every trial. Green dots indicate first-reach success trials, black dots indicate first reach failed trials (i.e., pellet remained, pellet knocked off, or multiple reach success), and gray dots indicate all other trials (e.g., no pellet delivered). Bottom plots show the difference between the mean value on each day and the mean value on day 1. Distributions show the results of a bootstrap resampling procedure with 95% confidence intervals indicated by the solid lines at the left of each distribution. C, Same as B for the mean distance from the average reach trajectory for each day (top panel shows all hand location data for a single rat that went into Fig. 2C, left panel; bottom panel shows all digit 2 location data that went into Fig. 4A, left panel). D, Same as B, C for the reach endpoint analyses. Left column shows reach endpoints in x, y, and z for the hand location (all data for a single rat that went into Fig. 3B, left panels). Right column shows reach endpoints for digit 2 with respect to the hand location (all data for a single rat that went into Fig. 3B, right panels, except here the hand location was subtracted out). Data and code to generate this figure are contained in Extended Data 1, 2, 10. Download Figure 6-13, TIF file. [file enu-eN-NWR-0153-21-s20.tif]

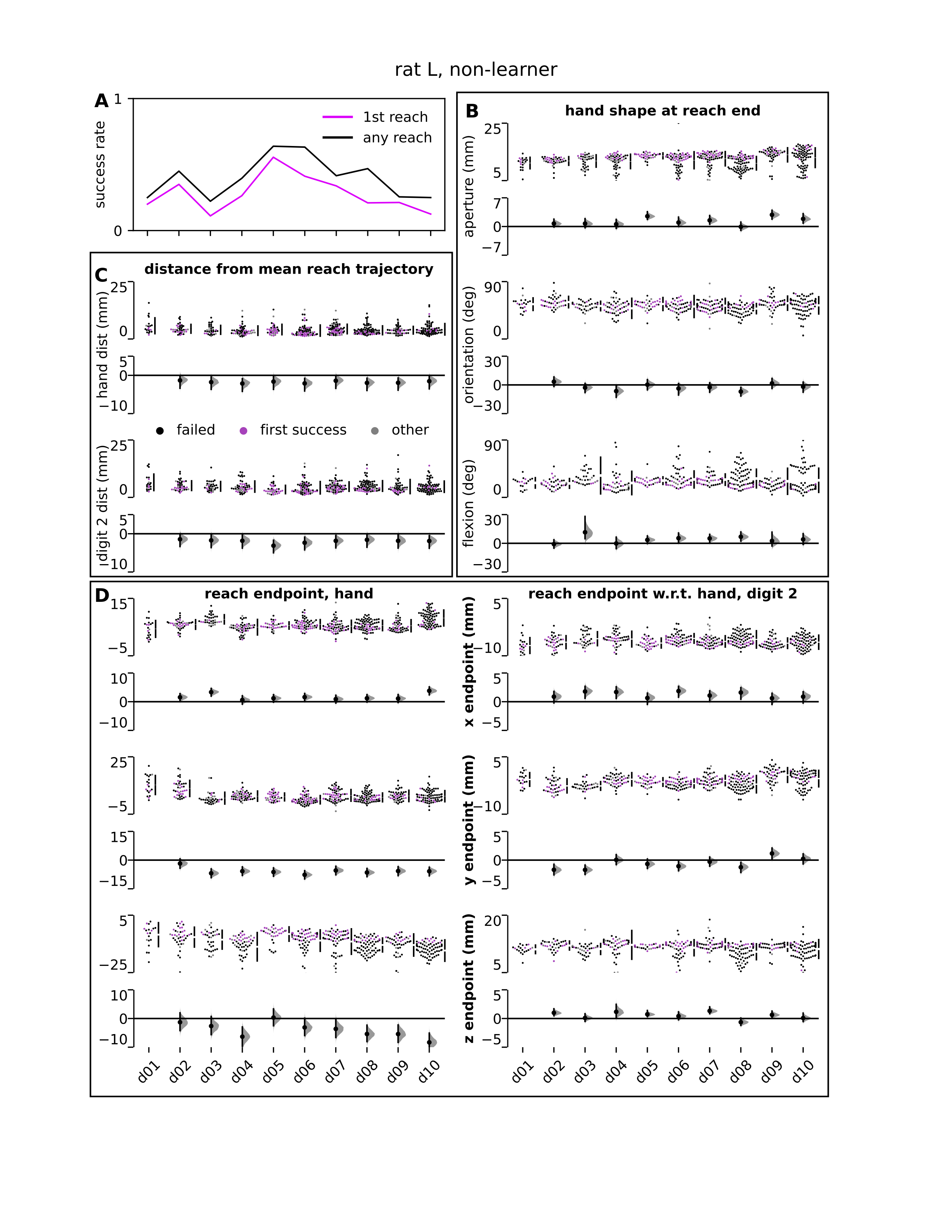

Supplement: Extended Data Figure 6-14 — Kinematics summary sheet for a nonlearner rat. A, Success rate across days. Pink lines indicate first reach success, black lines indicate success on any reach attempt for a single trial. B, Shared control plots illustrating measures of hand shaping at reach end (all data for a single rat that went into Fig. 4C–N). The top axes are swarm plots showing aperture at reach end for every trial. Pink dots indicate first-reach success trials, black dots indicate first reach failed trials (i.e., pellet remained, pellet knocked off, or multiple reach success), and gray dots indicate all other trials (e.g., no pellet delivered). Bottom plots show the difference between the mean value on each day and the mean value on day 1. Distributions show the results of a bootstrap resampling procedure with 95% confidence intervals indicated by the solid lines at the left of each distribution. C, Same as B for the mean distance from the average reach trajectory for each day (top panel shows all hand location data for a single rat that went into Fig. 2C, left panel; bottom panel shows all digit 2 location data that went into Fig. 4A, left panel). D, Same as B, C for the reach endpoint analyses. Left column shows reach endpoints in x, y, and z for the hand location (all data for a single rat that went into Fig. 3B, left panels). Right column shows reach endpoints for digit 2 with respect to the hand location (all data for a single rat that went into Fig. 3B, right panels, except here the hand location was subtracted out). Data and code to generate this figure are contained in Extended Data 1, 2, 11. Download Figure 6-14, TIF file. [file enu-eN-NWR-0153-21-s21.tif]

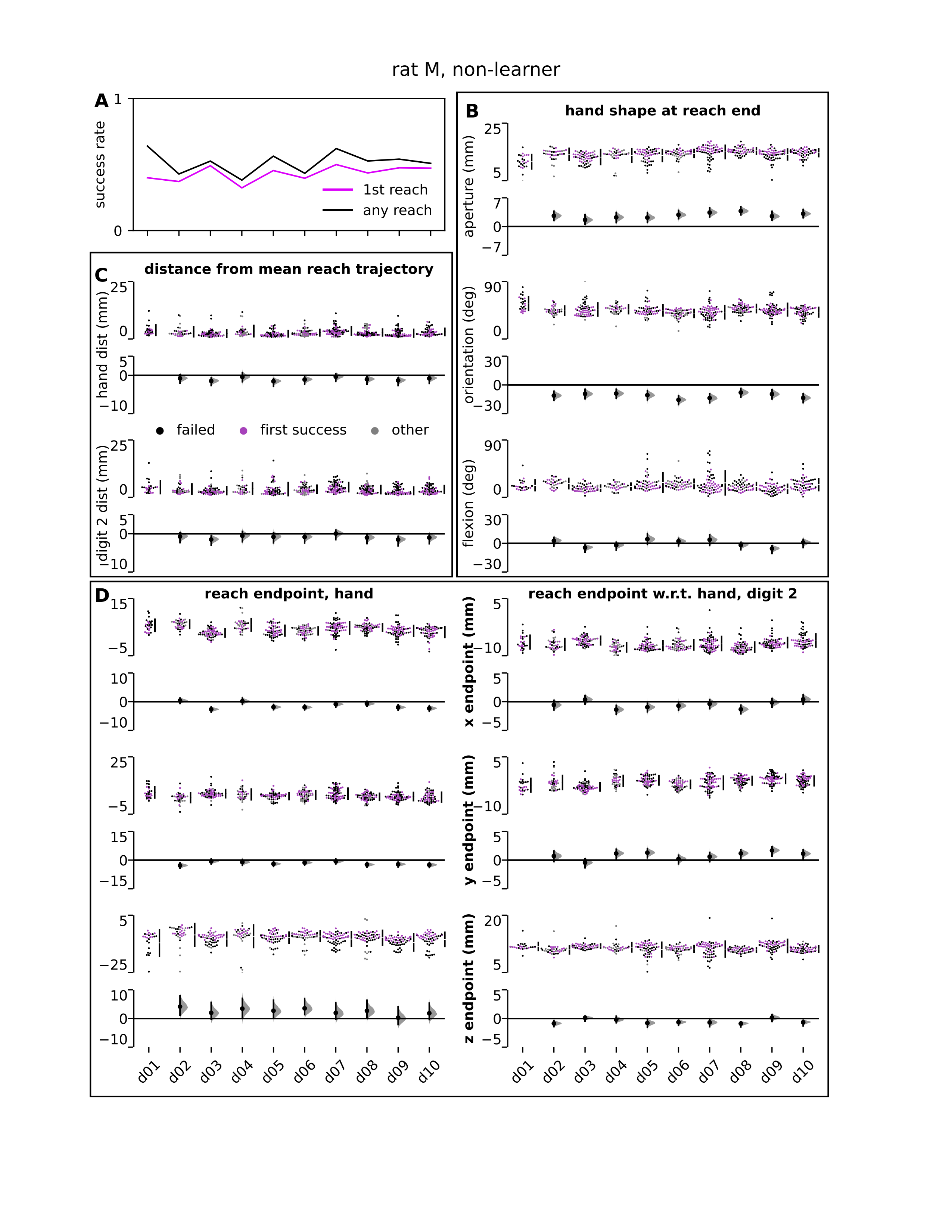

Supplement: Extended Data Figure 6-15 — Kinematics summary sheet for a nonlearner rat. A, Success rate across days. Pink lines indicate first reach success, black lines indicate success on any reach attempt for a single trial. B, Shared control plots illustrating measures of hand shaping at reach end (all data for a single rat that went into Fig. 4C–N). The top axes are swarm plots showing aperture at reach end for every trial. Pink dots indicate first-reach success trials, black dots indicate first reach failed trials (i.e., pellet remained, pellet knocked off, or multiple reach success), and gray dots indicate all other trials (e.g., no pellet delivered). Bottom plots show the difference between the mean value on each day and the mean value on day 1. Distributions show the results of a bootstrap resampling procedure with 95% confidence intervals indicated by the solid lines at the left of each distribution. C, Same as B for the mean distance from the average reach trajectory for each day (top panel shows all hand location data for a single rat that went into Fig. 2C, left panel; bottom panel shows all digit 2 location data that went into Fig. 4A, left panel). D, Same as B, C for the reach endpoint analyses. Left column shows reach endpoints in x, y, and z for the hand location (all data for a single rat that went into Fig. 3B, left panels). Right column shows reach endpoints for digit 2 with respect to the hand location (all data for a single rat that went into Fig. 3B, right panels, except here the hand location was subtracted out). Data and code to generate this figure are contained in Extended Data 1, 2, 12. Download Figure 6-15, TIF file. [file enu-eN-NWR-0153-21-s22.tif]

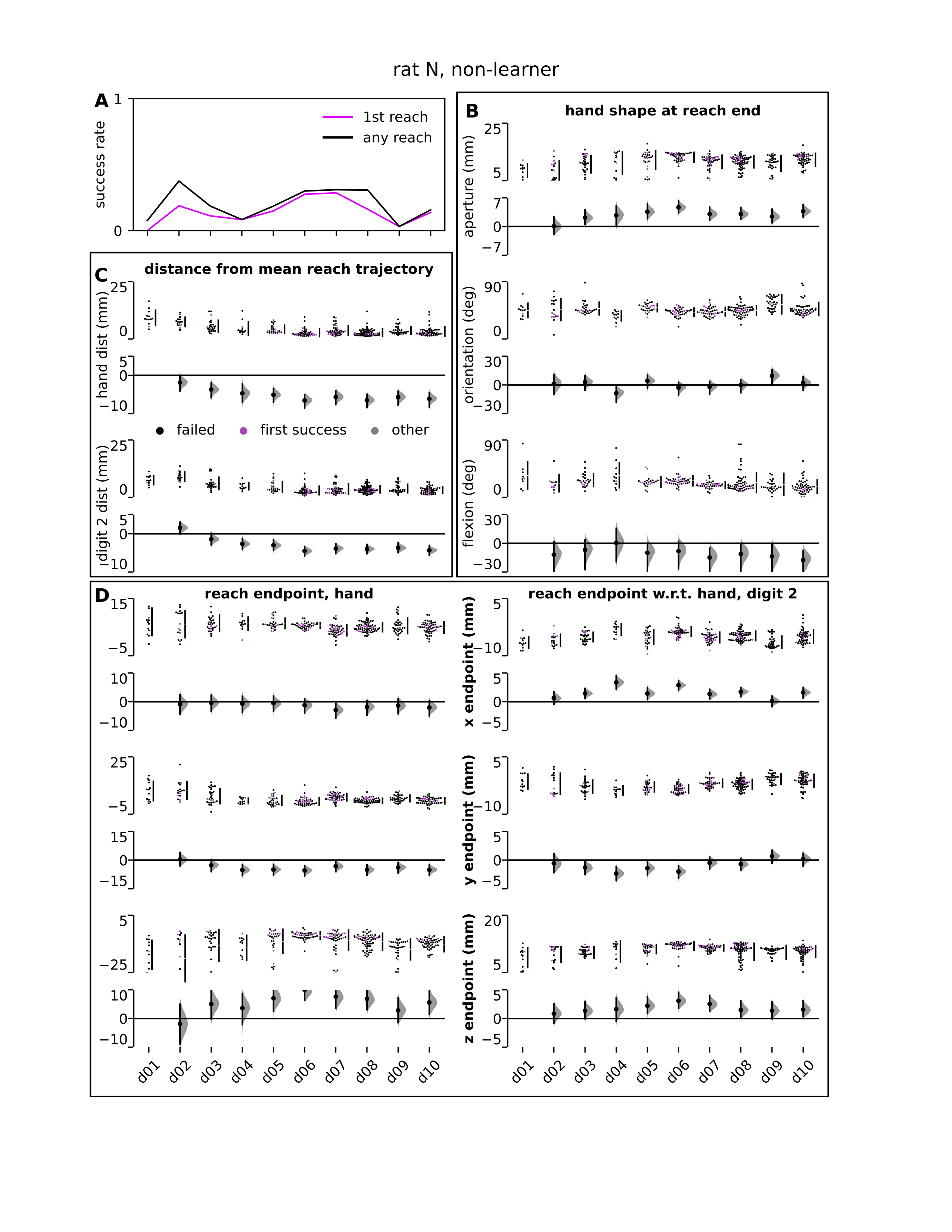

Supplement: Extended Data Figure 6-16 — Kinematics summary sheet for a nonlearner rat. A, Success rate across days. Pink lines indicate first reach success, black lines indicate success on any reach attempt for a single trial. B, Shared control plots illustrating measures of hand shaping at reach end (all data for a single rat that went into Fig. 4C–N). The top axes are swarm plots showing aperture at reach end for every trial. Pink dots indicate first-reach success trials, black dots indicate first reach failed trials (i.e., pellet remained, pellet knocked off, or multiple reach success), and gray dots indicate all other trials (e.g., no pellet delivered). Bottom plots show the difference between the mean value on each day and the mean value on day 1. Distributions show the results of a bootstrap resampling procedure with 95% confidence intervals indicated by the solid lines at the left of each distribution. C, Same as B for the mean distance from the average reach trajectory for each day (top panel shows all hand location data for a single rat that went into Fig. 2C, left panel; bottom panel shows all digit 2 location data that went into Fig. 4A, left panel). D, Same as B, C for the reach endpoint analyses. Left column shows reach endpoints in x, y, and z for the hand location (all data for a single rat that went into Fig. 3B, left panels). Right column shows reach endpoints for digit 2 with respect to the hand location (all data for a single rat that went into Fig. 3B, right panels, except here the hand location was subtracted out). Data and code to generate this figure are contained in Extended Data 1, 2, 13. Download Figure 6-16, TIF file. [file enu-eN-NWR-0153-21-s23.tif]
