# Supplementary material for: Evolution of Gross Forelimb and Fine Digit Kinematics during Skilled Reaching Acquisition in Rats
Source: eNeuro. 2021 Oct 26;8(5):ENEURO.0153-21.2021. doi: 10.1523/ENEURO.0153-21.2021 (PMC8555885; doi:10.1523/ENEURO.0153-21.2021)
Supplement: Extended Data 1 — MATLAB and Python code for kinematic analyses and figure plotting. Bova_Leventhal_code.zip contains custom MATLAB software for reconstruction of 3D reach trajectories, processing reach-to-grasp kinematics, and creating the figures in this manuscript using data in the other Extended Data files. Download Extended Data 1, ZIP file. [file enu-eN-NWR-0153-21-s24.zip › DLC_analysis_scripts/1_analysis_workflow.docx]

Skilled Reaching Analysis Workflow

Box Calibration

1. Run script_detect_calibration_points to run one month at a time. This will detect as many checkerboard corners as it can and save them to a folder YYYYMM_auto_marked. It will save an “_auto.mat” file that contains the point coordinates and a .png file with the calibration image with points marked on it.
2. Scan through the saved “marked” .png images. If any points aren’t marked or points are mismarked, load into Fiji. Mark the checkerboard points that need to be added and save as a “marked.tif”. Get the coordinates (“measure” or command-M), and save the .csv file as “GridCalibration_*YYYYMMDD*_*img#*.csv”. Save these files into a new folder YYYYMM_manually_marked.
3. Run script_add_manual_marks. This will match the .csv files with the images and determine which points match up in each view. It will save “_all.mat” files with all of the point markings and an “_all_marked.png” file. These are saved to a newly created folder YYYYMM_all_marked. Circles are automatically found points, squares are marked by the users in Fiji.
4. Run script_calibrateBoxes. This takes all the matched points and calculates transformation matrices for 3D reconstructions. It stores them in a newly created folder YYYYMM_calibration_files in a file called “SR_boxCalibration_box*##*_YYYMMDD.mat”.
5. Run script_checkDLCBoxCalibration.m to check that the calibration worked.
   1. For each date, the script will generate graphs plotting the marked points. Check that the graphs have 3 normal looking rectangular planes and then continue the script to move to the next date.
      1. If the graphs do not look normal, go back and run the previous steps.

Kinematic Processing

1. Recalibrate the boxes for each session. Run script_recalibrateBoxes. Make sure that the relevant box calibration file for that session has already been calculated; otherwise, the script will go back in time until it finds a calibration file for that box/
2. Reconstruct the 3D trajectories. Run script_reconstruct3Dtrajectories. Make sure the repeatCalculations flag is set appropriately.
3. Run script_calculateKinematics, which will move the origin to the initial pellet location and calculate a variety of kinematic features (velocity, aperture, etc.)
4. Interpolate the trajectories. Run script_interp_trajectories
5. Run script_analyze_interp_trajectories. This will identify individual reaches from each trial and calculate some summary statistics for each session (average trajectories, variances, etc.). see script comments for details
6. Run script_calculateRatSummaries.
7. Run script_collectRatSummaries_learning.
8. Run script_calculateOutcomeTrajectories
9. Run script_collectOutcomeDistributions
10. Run script_collectAverageKinematicsByOutcome
